# The transcription factor Xrp1 orchestrates both reduced translation and cell competition upon defective ribosome assembly or function

**DOI:** 10.7554/eLife.71705

**Published:** 2022-02-18

**Authors:** Marianthi Kiparaki, Chaitali Khan, Virginia Folgado-Marco, Jacky Chuen, Panagiotis Moulos, Nicholas E Baker

**Affiliations:** 1 Department of Genetics, Albert Einstein College of Medicine The Bronx United States; 2 Institute for Fundamental Biomedical Research, Biomedical Sciences Research Center "Alexander Fleming” Vari Greece; 3 Department of Developmental and Molecular Biology, Albert Einstein College of Medicine The Bronx United States; 4 Department of Opthalmology and Visual Sciences, Albert Einstein College of Medicine The Bronx United States; New York University School of Medicine United States; Columbia University United States

**Keywords:** ribosome, ribosomopathy, ribosomal protein, cell competition, xrp1 gene, translation, copia, *D. melanogaster*

## Abstract

Ribosomal Protein (*Rp*) gene haploinsufficiency affects translation rate, can lead to protein aggregation, and causes cell elimination by competition with wild type cells in mosaic tissues. We find that the modest changes in ribosomal subunit levels observed were insufficient for these effects, which all depended on the AT-hook, bZip domain protein Xrp1. Xrp1 reduced global translation through PERK-dependent phosphorylation of eIF2α. eIF2α phosphorylation was itself sufficient to enable cell competition of otherwise wild type cells, but through Xrp1 expression, not as the downstream effector of Xrp1. Unexpectedly, many other defects reducing ribosome biogenesis or function (depletion of TAF1B, eIF2, eIF4G, eIF6, eEF2, eEF1α1, or eIF5A), also increased eIF2α phosphorylation and enabled cell competition. This was also through the Xrp1 expression that was induced in these depletions. In the absence of Xrp1, translation differences between cells were not themselves sufficient to trigger cell competition. Xrp1 is shown here to be a sequence-specific transcription factor that regulates transposable elements as well as single-copy genes. Thus, Xrp1 is the master regulator that triggers multiple consequences of ribosomal stresses and is the key instigator of cell competition.

## Introduction

It would be difficult to exaggerate the importance of ribosomes. Eukaryotic ribosomes comprise 4 rRNAs and 80 proteins combined into Large and Small subunits (LSU and SSU) that, together with multiple initiation and elongation factors, constitute the translational apparatus for protein synthesis (Jackson et al., 2010; Thomson et al., 2013). Ribosome biogenesis, and the regulation of translation, are important targets of cellular regulation, and defects affecting ribosomes and translation are implicated in many diseases, from neurodegeneration to cancer (Aspesi and Ellis, 2019; Hetman and Slomnicki, 2019; Genuth and Barna, 2018; Ingolia et al., 2019; Joazeiro, 2019; Phillips and Miller, 2020). Mutations affecting rRNA synthesis, ribosomal protein genes (Rp genes), and some other ribosome biogenesis factors give rise to ribosomopathies, a family of translation-related diseases (Kampen et al., 2020). The ribosomopathy Diamond Blackfan Anemia (DBA) most commonly results from heterozygosity for mutations in Rp genes, and is characterized by early onset anemia, cancer predisposition, and sometimes diminished growth and skeletal defects (Draptchinskaia et al., 1999; Choesmel et al., 2007; Danilova and Gazda, 2015; Da Costa et al., 2018). Most ribosomal protein genes are also haploinsufficient in *Drosophila*, where their dominant ‘Minute’ phenotype was named by Bridges and Morgan on account of the small, thin cuticular bristles observed, in addition to developmental delay (Bridges and Morgan, 1923; Lambertsson, 1998; Marygold et al., 2007) .

*Rp* gene loci were recently proposed to be important indicators of aneuploidy (Ji et al., 2021). Aneuploid cells can be selectively eliminated from embryonic and developing mammalian tissues, but the mechanisms responsible have been uncertain (Bolton et al., 2016; McCoy, 2017). In *Drosophila*, cells heterozygous for mutations in *Rp* genes are selectively eliminated from mosaic imaginal discs, where they are replaced by neighboring wild-type cells (Morata and Ripoll, 1975; Simpson, 1979). This phenomenon, named ‘cell competition’, represents a process whereby cells that present differences from their neighbors can be eliminated from growing tissues, thought to enable the removal of cells that might be deleterious to the tissue (Morata and Ripoll, 1975; Lawlor et al., 2020; Baker, 2020; Vishwakarma and Piddini, 2020; Marques-Reis and Moreno, 2021; Morata, 2021). Because *Rp* gene dose is likely to be affected whenever one or more chromosomes or substantial chromosome regions are monosomic, cell competition could help eliminate aneuploid cells on the basis of altered *Rp* gene dose (McNamee and Brodsky, 2009). This mechanism indeed occurs in *Drosophila* imaginal discs (Ji et al., 2021). Such a role of cell competition is potentially significant for tumor surveillance, since tumors almost always consist of aneuploid cells, and for healthy aging, since aneuploid cells accumulate during aging (Hanahan and Weinberg, 2011; López-Otín et al., 2013). In addition to their mutation in DBA, this provides another reason why it is important to understand the cellular effects of *Rp* mutations, and how they lead to cell competition.

Unsurprisingly, *Rp* mutant heterozygosity generally leads to reduced translation (Boring et al., 1989; Oliver et al., 2004; Lee et al., 2018). It might be expected that a 50% reduction in ribosome subunit biogenesis would be responsible, but remarkably, in *Drosophila* this and many other features of *Rp* haploinsufficiency, including cell competition in the presence of wild-type cells, depend on a bZip, AT-hook putative transcription factor encoded by the *Xrp1* gene (Lee et al., 2018). *Xrp1* is responsible for >80% of the transcriptional changes that are seen in *Rp^+/-^*wing imaginal discs (Lee et al., 2018). Thus, reduced translation, which is a feature of Rp haploinsufficiency from yeast to mice and humans, may have a transcriptional basis (Lee et al., 2018). Accordingly, we could detect only modest reductions in SSU concentration in heterozygous *RpS3*, *RpS17,* or *RpS18* mutants, although *RpL27A* haploinsufficiency reduced steady state LSU numbers by ~30% (Lee et al., 2018). Some of these findings now have support from yeast studies, where deletion of single *Rp* loci present in paralogous pairs (a recent genome duplication has left yeast with many such *Rp* gene pairs) potentially mimics heterozygosity for a single copy gene in diploid organisms. The large majority of translational changes described by ribosome profiling of such yeast pseudo-heterozygotes turned out to reflect changes in mRNA abundance, implicating a predominantly transcriptional response to *Rp* mutations in yeast also (Cheng et al., 2019). Mass spectrometry and rRNA measurements of the yeast strains further suggested that ribosome numbers are little affected in most *RpL* gene deletion strains, whereas some *RpS* deletions increase LSU concentrations by up to 1.5 x (Cheng et al., 2019). There is also evidence from mice, where it is now suggested that reduced translation in *RpS6^+/-^* mouse cells depends on the transcription factor p53 (Tiu et al., 2021).

These findings raise many mechanistic questions. How does *Rp* haploinsufficiency activate *Xrp1* gene expression?How does this putative transcription factor control overall translation, if not through altered ribosome numbers? Are differences in translation rate between cells the cause of cell competition, or is cell competition due to other consequences of Xrp1 activity?

Alternative views of the *Rp* mutant phenotype have also been presented. Aside from the idea that reduced ribosome levels alter translation directly and are predominantly responsible for human DBA (Mills and Green, 2017; Khajuria et al., 2018), two recent studies propose that degradation of excess orphan Rp suppresses proteasome and autophagic flux in *Drosophila Rp* mutants, leading to protein aggregation and proteotoxic stress. They propose that proteotoxic stress suppresses translation, and renders *Rp*^+/-^ cells subject to competition with wild-type cells through a further oxidative stress response (Baumgartner et al., 2021; Recasens-Alvarez et al., 2021). In addition, in concluding that autophagy is protective for *Rp* mutant cells (Baumgartner et al., 2021; Recasens-Alvarez et al., 2021), these studies contradict previous conclusions that autophagy is only increased in *Rp* mutant cells next to wild-type cells, where it promotes cell death (Nagata et al., 2019).

Here, we further investigate the basis of the *Rp* mutant phenotype in *Drosophila*. The results reaffirm the central role of Xrp1 in multiple aspects of the *Rp* mutant phenotype. We confirm the modest effects of *Rp* haploinsufficiency on numbers of mature ribosome subunits, and show directly that ribosome precursors accumulate in *Rp* mutants. We find that translation is reduced in *Rp* mutant cells through eIF2α phosphorylation, but both this and the protein aggregation observed (which appears specific for mutations affecting SSU proteins) require Xrp1 and so are not direct post-transcriptional consequences of ribosome assembly defects, as had been suggested (Baumgartner et al., 2021; Recasens-Alvarez et al., 2021). We report that interfering with translation, whether through eIF2α phosphorylation or by multiple other routes disrupting ribosome assembly or function, can subject otherwise wild-type cells to competition with normal cells. This is not because translation differences between cells cause cell competition directly, however, but because defects in both ribosome biogenesis and function that affect translation are all found to activate Xrp1, which then mediates the cell competition engendered by these translational stresses. We then show that Xrp1 is a sequence-specific transcription factor that is required for cell competition in response to multiple triggers and is responsible for multiple aspects of the *Rp* mutant phenotype, potentially including transcription of genes that have previously been taken as reporters of oxidative stress. Altogether, these studies clarify discrepancies in previously published work, and refocus attention on transcriptional responses to ribosome and translation defects mediated by Xrp1, with implications for the mechanisms and therapy of multiple ribosomopathies, and for the surveillance of aneuploid cells.

## Results

### Ribosome Levels in *Rp**^+/-^*** Cells

Abnormal cellular levels of ribosome subunits has been proposed as the basis for reduced translation in ribosomopathies (Mills and Green, 2017). Multiple models of DBA accordingly seek to reduce steady-state Rp concentration to 50% of normal (Heijnen et al., 2014; Khajuria et al., 2018). By measuring *Drosophila* rRNA levels in northern blots, however, we had previously concluded that whereas cellular levels of ribosome subunits were affected in heterozygotes for an *RpL27A* mutant, multiple *Rp* mutations affecting SSU proteins led only to ~10% reduction in SSU levels that was not statistically significant (Lee et al., 2018). A caveat to this conclusion was the use of tubulin mRNA and actin mRNA as loading controls. While mRNA-seq shows that the proportions of actin and tubulin mRNAs are not much affected in *Rp*^+/-^ genotypes (Kucinski et al., 2017; Lee et al., 2018), it could be that total mRNA amounts are altered by *Rp* mutations, which would affect the conclusions regarding rRNA when mRNA standards are used. In bacteria, it is well-established that ribosomes protect mRNA from turnover, so that reduced ribosome numbers reduce overall mRNA levels as well (Yarchuk et al., 1992; Hui et al., 2014). The situation in eukaryotic cells may not be the same as in bacteria (Belasco, 2010). Still, we decided to measure rRNA levels again using a non-coding RNA as loading control. We chose the 7SL RNA component of Signal Recognition Particle, an abundant non-coding RNA that is expressed in all cells.

**Figure 1.**
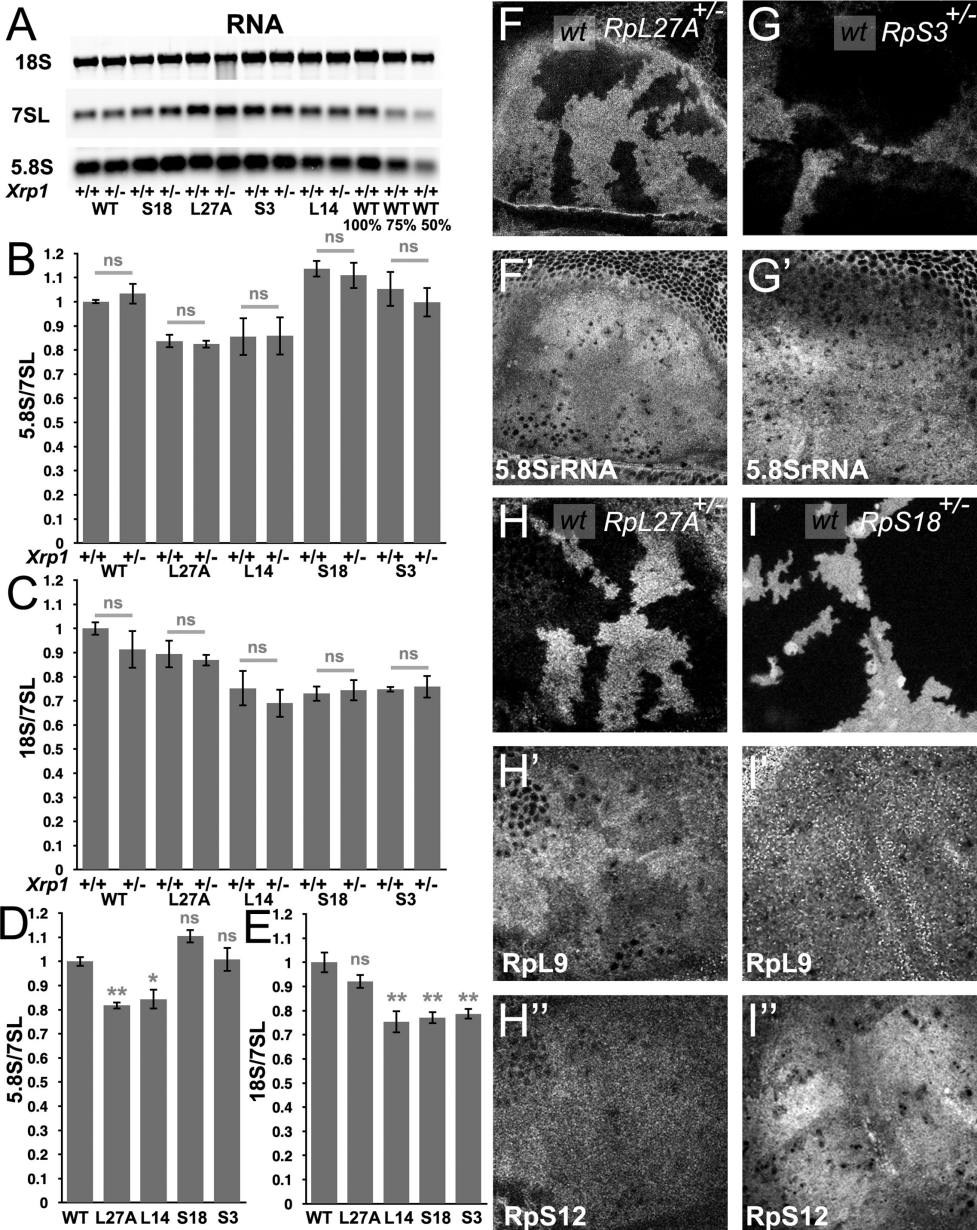
Modest changes in ribosomal subunit concentrations in *Rp* mutant wing discs. (**A**) Similar amounts of wing disc RNA from indicated genotypes separated and transferred for northern blotting with, in this case, probes specific for the 18 S rRNA of the ribosomal SSU, the 7SL non-coding RNA for the Signal Recognition Particle, and the 5.8 S rRNA of the ribosomal LSU. Right-most two lanes show serial dilutions of the wild type sample. Panels B-E show signal quantification from multiple such northerns. (**B**) *Xrp1* mutation did not affect LSU concentration in any *Rp* genotype. Significance shown only for *Xrp1^+/+^* to *Xrp1^+/-^* between otherwise similar genotypes. Padj values were one in all cases. (**C**) *Xrp1* mutation did not affect SSU concentration in any *Rp* genotype. Significance shown only for *Xrp1^+/+^* to *Xrp1^+/-^* between otherwise similar genotypes. Padj values were one in all cases. (**D**) Two *RpL* mutations reduced LSU concentrations. Significance shown only for comparisons between mutant genotypes and the wild type. Exact Padj values were: 0.00423, 0.0117, 0.0877, 0.858 respectively. (**E**) Two *RpS* mutations, as well as *RpL14*, reduced SSU concentrations. Significance shown only for comparisons between mutant genotypes and the wild type. Exact Padj values were: 0.135, 0.000218, 0.000395, 0.000602 respectively. WT genotype: p{hs:FLP}/w118; p{arm:LacZ} FRT80B/+, Xrp1^+/-^ genotype: p{hs:FLP}/w118; FRT82B *Xrp1^M2^*^–73^/+, *L27A*^+/-^ genotype: p{hs:FLP}/ p{hs:FLP}; *RpL27A*- p{arm:LacZ}FRT40/+; FRT80B/+, *L27A*^+/-^; *Xrp1*^+/-^ genotype: p{hs:FLP}/ p{hs:FLP}; *RpL27A*- p{arm:LacZ}FRT40/+; FRT82B *Xrp1^M2^*^–73^/+, *L14*^+/-^ genotype: p{hs:FLP}/ p{hs:FLP}; FRT42/+; *RpL14*^1^ /+, *L14*^+/-^; *Xrp1*^+/-^ genotype: p{hs:FLP}/ p{hs:FLP}; FRT42/+; *RpL14^1^*/ FRT82 B *Xrp1^M2^*^–73^, S3^+/-^ genotype: p{hs:FLP}/ p{hs:FLP}; FRT42/+; FRT82 *RpS3* p{arm:LacZ}/+, S3^+/-^; Xrp1^+/-^ genotype: p{hs:FLP}/ p{hs:FLP}; FRT82 *RpS3* p{arm:LacZ}/FRT82B *Xrp1^M2^*^–73^, *S18*^+/-^ genotype: p{hs:FLP}/ p{hs:FLP}; FRT42 *RpS18* p{ubi:GFP} /+; FRT80B/+, *S18*^+/-^; Xrp1^+/-^ genotype: p{hs:FLP}/ p{hs:FLP}; FRT42 *RpS18* p{ubi:GFP} /+; FRT82B *Xrp1^M2^*^–73^/+ Panels F-I show comparisons between antibody labelings of 5.8 S rRNA, anti-RpL9, or anti-RpS12 between wild type and *Rp^+/-^* cells in mosaic wing imaginal discs. (**F,F’**) *RpL27A* mutation reduced levels of 5.8SrRNA. (**G,G’**) *RpS3* mutation had negligible effect on 5.8 S rRNA levels. (**H,H’,H”**) *RpL27A* mutation reduced levels of the LSU component RpL9 but a small effect on the SSU component RpS12. (**I,I’,I”**) *RpS18* mutation reduced levels of the SSU component RpS12 but not of the LSU component RpL9. Statistics:One-way Anova with Bonferroni-Holm multiple comparison correction was performed for panels B-E, which were each based on three biological replicates. ns - p ≥ 0.05.* - p < 0.05.** - p < 0.01. Genotypes: F, H: p{hs:FLP}/ p{hs:FLP}; RpL27A^-^ p{arm:LacZ} FRT40/FRT40, G: p{hs:FLP}/ p{hs:FLP}; FRT82 *RpS3* p{arm:LacZ} /FRT82B, I: p{hs:FLP}/ p{hs:FLP}; FRT42 *RpS18* p{Ubi:GFP}/FRT42. Figure 1—source data 1.Full and unedited blots corresponding to panel A. Figure 1—source data 2.Northern data underlying panels B-E.

Changes in LSU and SSU levels inferred from 5.8 S and 18 S rRNA abundance, normalized to 7SL RNA levels, are shown in Figure 1, and a representative northern blot in Figure 1A. Similar to what was observed previously, Xrp1 mutations had no effect on apparent LSU or SSU levels in the wild type or in heterozygotes for any of four mutant loci, *RpS18*, *RpS3*, *RpL27A*, and *RpL14*, reaffirming that Xrp1 is unlikely to affect translation rate through an effect on ribosome subunit concentrations (Figure 1B and C). Accordingly, *Xrp1^+/+^* and *Xrp1^+/-^* data were combined together to compare the effects of *Rp* mutations. We confirmed that LSU numbers were reduced in the *RpL27A* mutant, and extended this observation to mutation in a second RpL gene, *RpL14* (Figure 1D). Unlike our previous study, SSU levels were reduced 20%–30% in *RpS18*, *RpS3,* and *RpL14* mutants when normalized to the non-coding 7SL RNA, and these reductions were significantly different from the control (Figure 1E). By contrast, *RpL27A* did not change SSU numbers (Figure 1E).

**Figure 1—figure supplement 1.**
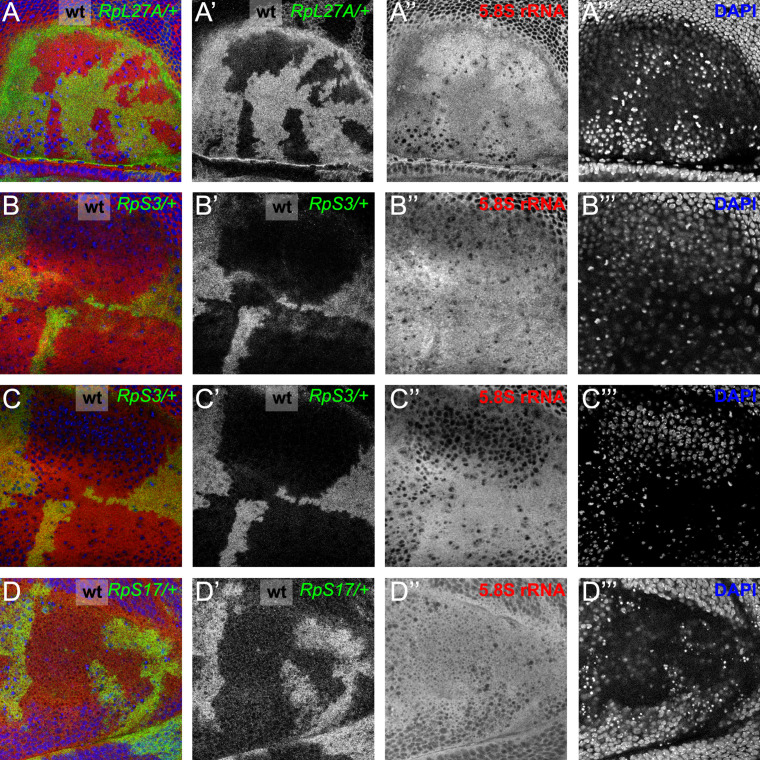
Cytoplasmic location of ribosome components. Specimens including those from Figure 1F and G showing the nuclear channel that was recorded simultaneously in many experiments. (**A**) This very apical confocal plane passes through only a few nuclei, particularly in wild type cells, verifying that the anti-5.8S rRNA signal is predominantly cytoplasmic, consistent with mature LSU. (**B**) This very apical confocal plane largely excludes nuclei, verifying that the anti-5.8S rRNA signal is predominantly cytoplasmic, consistent with mature LSU. (**C**) This slightly less apical focal plane predominantly grazes nuclei only of wild type cells, verifying that little anti-5.8S rRNA signal is nuclear. (**D**) This very basal confocal plane, which largely excludes nuclei for wild type regions, shows no discernible difference in cytoplasmic anti-5.8S rRNA signal between wild type and *RpS17*/+ cells. Genotypes: A: p{hs:FLP}/ p{hs:FLP}; RpL27A^-^ p{arm:LacZ} FRT40/FRT40, B,C: p{hs:FLP}/ p{hs:FLP}; FRT82 *RpS3* p{arm:LacZ} /FRT82B, D: p{hs:FLP}/+; *RpS17* p{arm:LacZ} FRT80B/FRT80B.

**Figure 1—figure supplement 2.**
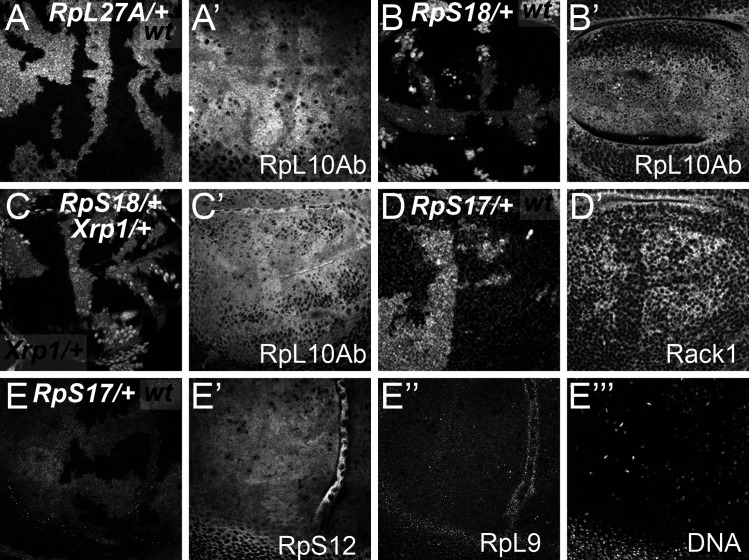
More examples of ribosome levels in *Rp* mutant genotypes. Panels (**A–E**) show mosaic wing discs containing *Rp^+/+^* cells (black) and cells of indicated *Rp^+/-^* genotypes (white). Panels (**A’-E’ and E’’**) show these same discs labelled with the antibodies against the indicated Rp. Panel E’’’ indicates DNA, in these cases indicating an apical confocal plane above most nuclei. (**A**) *RpL27A^+/-^* cells contained less of the LSU component RpL10Ab. (**B**) *RpS18^+/-^* cell contained more of the LSU component RpL10Ab. (**C**) *RpS18^+/-^ Xrp1^+/-^* cells contained more of the LSU component RpL10Ab than *RpS18^+/+^Xrp1^+/-^* cells. (**D**) *RpS17^+/-^* cell contained less of the SSU component Rack1. (**E**) *RpS17^+/-^* cell contained less of the SSU component RpS12 but levels of the LSU component RpL9 were indistinguishable from *RpS17^+/+^* cells. Genotypes: A: p{hs:FLP}/ p{hs:FLP}; RpL27A^-^ p{arm:LacZ} FRT40/FRT40, B: p{hs:FLP}/ p{hs:FLP}; FRT42 *RpS18* p{Ubi:GFP}/FRT42, C: p{hs:FLP}/ p{hs:FLP}; FRT42 *RpS18* p{Ubi:GFP}/FRT42; FRT82B Xrp1^M2-73^/+,D-E: p{hs:FLP}/+; *RpS17* p{arm:LacZ} FRT80B/FRT80B.

To confirm these findings using an independent method, we performed tissue staining with a monoclonal antibody, mAbY10B, that recognizes rRNA, and particularly a structure in the 5.8 S rRNA that is part of the LSU (Lerner et al., 1981). Consistent with Northern analysis, immunostaining of mosaic wing imaginal discs confirmed lower 5.8 S rRNA levels in *Rp27A*^+/-^ cells compared to *Rp27A*^+/+^ cells in the same wing discs (Figure 1F, Figure 1—figure supplement 1A). By contrast, no reduction in mAbY10B staining was observed in cell mutated for either of two SSU components, *RpS3* or *RpS17*, consistent with the northern blot measurements of 5.8 S rRNA levels (Figure 1G, Figure 1—figure supplement 1B-D).

To gain further support for these findings, we compared Rp protein levels by immunostaining mutant and control cells in the same imaginal discs. We used antibodies against RpL10Ab as markers for LSU, and against RpS12 and RACK1 as markers for SSU. *RpL27A* mutant cells contained lower levels of LSU protein, and slightly lower levels of SSU protein (Figure 1H, Figure 1—figure supplement 2A). *RpS17*, and *RpS18* mutant cells contained lower levels of the SSU protein, and *RpS18* slightly higher levels of the LSU protein RpL10Ab, even in the Xrp1 mutant background (Figure 1I, Figure 1—figure supplement 2B-E). These tissue staining experiments qualitatively support the conclusion that levels of SSU components are generally reduced in *RpS^+/-^* cells, whereas LSU levels were only reduced in *RpL^+/-^* cells (*RpL27A^+/-^),* in comparison to wild type cells within the same preparation, and that these changes are modest and unaffected by Xrp1, even though *Xrp1* mutation restores normal global translation rate (Lee et al., 2018).

### Ribosome Precursors Accumulate in *Rp^+/-^* Cells

**Figure 2.**
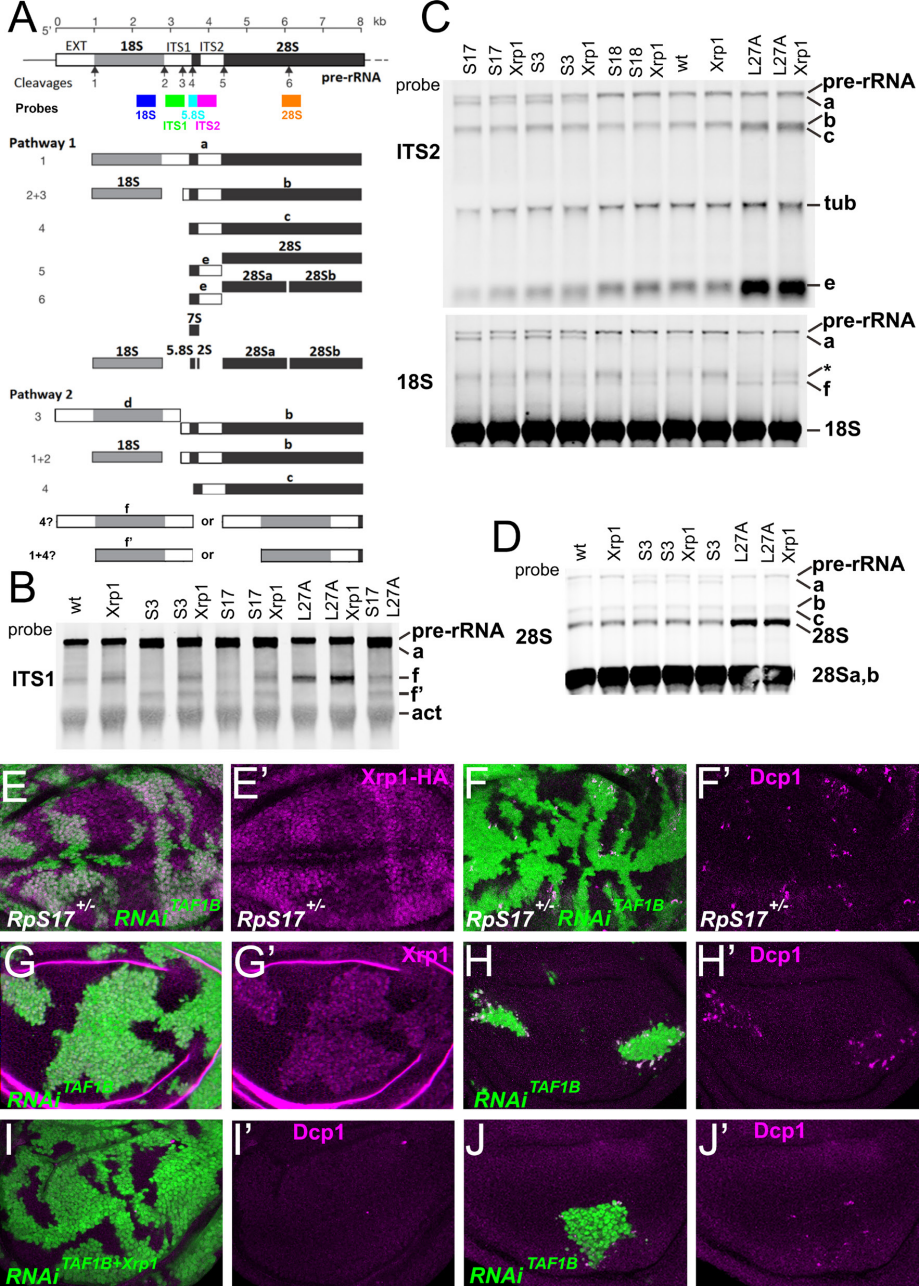
Ribosome biogenesis defects and their consequences. (**A**) Two pathways of rRNA processing and the intermediates that result were characterized in *D. melanogaster* embryos by Long and Dawid. Mature 18 S, 5.8 S and 28Sa,b rRNAs are processed from the pre-RNA, along with the removal of two interval sequences ITS1 and ITS2. The cleavages sites were described by Long and Dawid. Colored boxes indicate the probes used in the present study. The 5.8 S probe overlaps with 147 bases at 3’of the ITS1 region, excluding cleavage site 3. Additional intermediates f and **f’** were observed in the wing imaginal disc samples. These were recognized by ITS1, 5.8 S (Figure 2—figure supplement 1) and 18 S probe and therefore extended beyond the cleavage site 3, although whether beyond site four was uncertain. (**B–D**) Northern blots of total RNA purified from wild-type and Rp^+/-^ wing discs, probed as indicated. (**B**) Reprobed with ITS1 after an initial actin probe. (**C**) Reprobed with ITS2 and then 18 S probes after an initial tubulin probe. Intermediates b, f and the 28 S rRNA (which in *Drosophila* is a precursor to the mature 28Sa and 28 Sb rRNAs) were detected in wild type and *Xrp1^+/-^* wing discs, other intermediates only in *Rp^+/-^* genotypes. *RpS3^+/-^* and *RpS17^+/-^* had lower levels of pre-RNA and intermediate (**f**) but accumulate intermediates (**a**) and (**f**’), which might indicate delays in cleavages 2 and 3. *RpS18^+/-^* had increased levels of pre-RNA and intermediate (**f**). *RpL27^+/-^* accumulated bands (**b**, **c**, **e**, and **f**) and 28 S. The effect on (**f**) suggests crosstalk between RpL27A and SSU processing. (**E–I**) show single confocal planes from mosaic third instar wing imaginal discs. (**E**) TAF1B depletion (green) increased Xrp1-HA levels in *RpS17*^+/-^ discs (magenta, see also E’). (**F**) TAF1B depletion (green) increased in *RpS17*^+/-^ discs led to cell death at the boundaries with undepleted cells (active Dcp1 staining in magenta, see also F’). (**G**) TAF1B depletion (green) also increased Xrp1 protein levels in *RpS17*^+/+^ discs (magenta, see also G’). (**H**) TAF1B depletion (green) led to cell death at the boundaries with undepleted cells (active Dcp1 staining in magenta, see also H’). (**I**) Co-depletion of Xrp1 with TAF1B (green) largely abolished cell death at the clone interfaces (active Dcp1 staining in magenta, see also I’). (**J**) Clones of cells depleted for TAF1B in parallel with panel I, showing reduced clones size and number (green), and competitive cells death at boundaries magenta, see also J’. Additional data related to this Figure is presented in Figure 2—figure supplement 1. Genotypes: Northerns: similar to Figure 1 and additionally: S17^+/-^ genotype: p{hs:FLP}/ p{hs:FLP}; FRT42/+; FRT80 *RpS17* p{arm:LacZ} /+, S17^+/-^; Xrp1^+/-^ genotype: p{hs:FLP}/ p{hs:FLP}; FRT80 *RpS17* p{arm:LacZ} /FRT82B *Xrp1^M2^*^–73^, S17^+/-^, L27A^+/-^ genotype: p{hs:FLP}/ p{hs:FLP}; RpL27A^-^ p{arm:LacZ} FRT40/+; FRT80 *RpS17* p{arm:LacZ} /+, E, F: p{hs:FLP}/+; UAS- RNAi^TAF1B^ /+;*RpS17*, act> CD2> Gal4, UAS-GFP /+ (line: v105873), G, H: p{hs:FLP}/+; UAS- RNAi^TAF1B^ /+;act> CD2> Gal4, UAS- GFP /+ (line: Bl 61957), I: p{hs:FLP}/+; UAS- RNAi^TAF1B^ /UAS-RNAi^Xrp1^;act> CD2> Gal4, UAS- GFP /+ (line: Bl 61957), J: p{hs:FLP}/+; UAS- RNAi^TAF1B^ /TRE-dsRed;act> CD2> Gal4, UAS- GFP /+ (line: Bl 61957) (processed in parallel with 2I). Figure 2—source data 1.Full and unedited blots corresponding to panel B. Figure 2—source data 2.Full and unedited blots corresponding to panel C. Figure 2—source data 3.Full and unedited blots corresponding to panel D.

An additional, or alternative, potential effect of *Rp* mutations is the accumulation of unused ribosome precursors and assembly intermediates. In yeast, depleting almost any Rp arrests ribosome biogenesis at some stage, reflecting individual requirements for ribosome assembly (Ferreira-Cerca et al., 2005; Ferreira-Cerca et al., 2007; Pöll et al., 2009; Woolford and Baserga, 2013; Henras et al., 2015). *Rp* haploinsufficiency might delay biogenesis at these same steps, perhaps leading to accumulation of particular precursor states. To assess ribosome biogenesis in *Rp^+/-^* mutants, intermediates were quantified by Northern blotting using probes specific for sequences that are excised from the rRNA as the ribosomes assemble and mature. In *Drosophila*, two parallel pathways A and B excise ITS1, ITS2, and the N-terminal EXT sequences, and process the resulting rRNAs, until the mature 28 S (processed into 28Sa and 28 Sb in *Drosophila*), 18 S and 5.8 S rRNAs are produced by the end of ribosome biogenesis (Figure 2A; Long and Dawid, 1980). We used specific probes to identify rRNA intermediates on northern blots (Figure 2A–D; Figure 2—figure supplement 1). As predicted, intermediates accumulated in each of the *Rp^+/-^* genotypes (see Figure 2 legend for details). These findings support the idea that *Rp* gene haploinsufficiency leads to ribosome biogenesis delays, and corresponding accumulation of assembly intermediates. In no case did *Xrp1* mutation eliminate the accumulation of intermediates in *Rp* mutant genotypes (Figure 2B–D; Figure 2—figure supplement 1). There were some changes noted in the intermediates that accumulated, however. For example, in *RpS17^+/-^* and *RpS13^+/-^* it seems that more band f accumulates when *Xrp1* is mutated, and less band a (Figure 2C).

**Figure 2—figure supplement 1.**
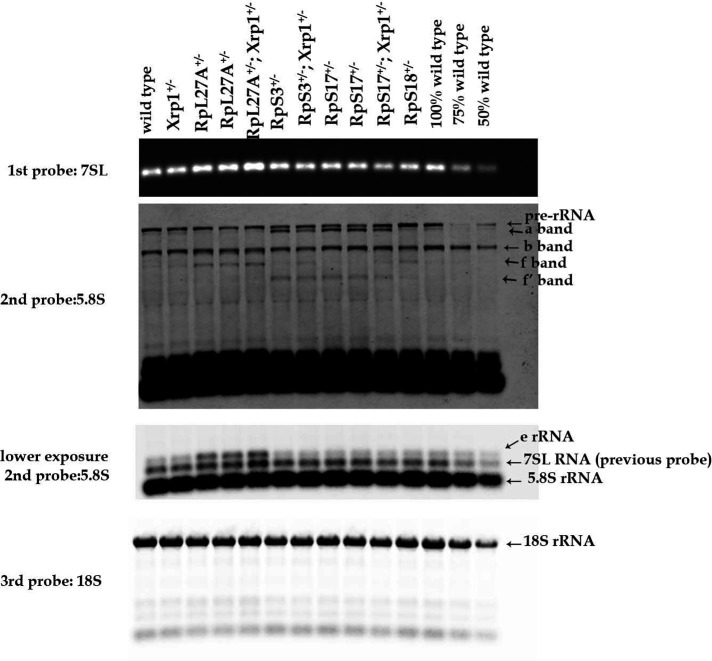
Additional northern blots detecting rRNA intermediates. Northern blots of total RNA purified from wild-type and Rp^+/-^wing discs, reprobed with 5.8 S probe and then 18 S probe after an initial 7SL probe. Figure 2—figure supplement 1—source data 1.Full and unedited blots corresponding to Figure 2—figure supplement 1.

**Figure 2—figure supplement 2.**
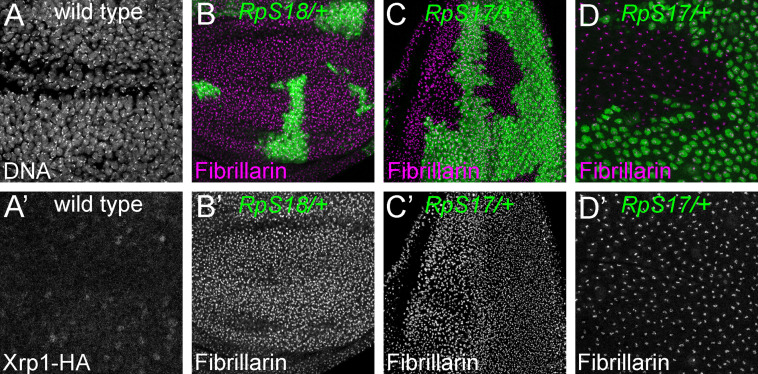
Nucleoli in wild type and *Rp* mutant cells. (**A**) Confocal section of Xrp1-HA wing disc showing many nuclei labelled for DNA. (**A’**) HA labeling reveals minimal Xrp1 expression in this otherwise wild type wing disc. Xrp1-HA is not obvious in nucleoli. (**B**) Mosaic wing disc containing *RpS18*^+/-^ cells (green). Anti-fibrillarin labeling of nucleoli reveals no obvious differences between *RpS18*^+/-^ and *RpS18*^+/+^ cells (projected in magenta; see also B’). (**C**) Mosaic eye disc containing *RpS17*^+/-^ cells (green). Anti-fibrillarin labeling of nucleoli reveals no obvious differences between *RpS17*^+/-^ and *RpS17*^+/+^ cells (projected in magenta; see also (**C’**)). (**D**) Peripodial membrane from mosaic eye eye disc containing *RpS17*^+/-^ cells (green). Anti-fibrillarin labeling of nucleoli reveals no obvious differences between *RpS17*^+/-^ and *RpS17*^+/+^ cells (projected in magenta; see also (**D’**)). Genotypes::A: p{hs:FLP}/ p{hs:FLP}; FRT42/FRT42; Xrp1-HA/Xrp1-HA B: p{hs:FLP}/ p{hs:FLP}; FRT42 *RpS18* p{Ubi:GFP}/FRT42, C,D: p{hs:FLP}/+; *RpS17* p{Ubi:GFP} FRT80B/FRT80B.

In mammalian cells with *Rp* haploinsufficiency, unincorporated 5 S RNP, comprising RpL5, RpL11 and the 5 S rRNA, activates the transcription factor and tumor suppressor p53 by inhibiting the p53 ubiquitin ligase MDM2 (Pelava et al., 2016). P53 is responsible for at least some consequences of *Rp* haploinsufficiency in mice, perhaps even including the reduction in translation (Tiu et al., 2021). P53 is also implicated in cell competition in mammals, although not in *Drosophila*, where Xrp1 may acquire some of its functions (Kale et al., 2015; Baker et al., 2019). In *Drosophila* it seems that RpS12 is particularly critical for activating Xrp1, through an unknown mechanism (Kale et al., 2018; Lee et al., 2018; Boulan et al., 2019; Ji et al., 2019). If a ribosome biogenesis intermediate, which might include RpS12, induced Xrp1 expression, then we predicted that its accumulation and signaling could be prevented by restricting rRNA biogenesis. To test this model, we reduced rRNA synthesis by knockdown of TAF1B, an accessory factor for RNA polymerase I (Knutson and Hahn, 2011). We predicted that Xrp1 expression would be reduced when TAF1B was knocked down in an *Rp*^+/-^ background, and that the knockdown cells would be more competitive than their *Rp*^+/-^ neighbors. Contrary to these predictions, Xrp1 expression was actually higher in *RpS17*^+/-^dsRNA^TAF1B^ cells than *RpS17*^+/-^cells (Figure 2E), and *RpS17*^+/-^ dsRNA^TAF1B^ cells underwent cell death at boundaries with *RpS17*^+/-^ territories, suggesting they were less competitive, not more so (Figure 2F). To understand this result, the effect of TAF1B knockdown in otherwise wild-type cells was examined, and found to resemble that of *RpS17*^+/-^dsRNA^TAF1B^ cells. That is, dsRNA^TAF1B^ cells strongly activated Xrp1 expression, and underwent apoptosis at interfaces with wild type cells (Figure 2G, H and J). This boundary cell death was Xrp1-dependent (Figure 2I and J). Thus, far from rRNA being required for Xrp1 expression and cell competition, as expected if an RNP containing RpS12 activates Xrp1, rRNA depletion appeared to have similar effects to Rp depletion.

It has been suggested that Xrp1 might normally be sequestered in nucleoli, only to be released by nucleolar disruption in *Rp^+/-^* cells (Baillon et al., 2018). We were unable to detect Xrp1 protein sequestered either in nucleoli or elsewhere in wild-type cells, and nucleoli appeared grossly normal in Rp^+/-^ cells by anti-fibrillarin staining, revealing no sign of nucleolar stress (Figure 2—figure supplement 2A-D). It is important to compare *Rp^+/-^* cells wild-type cells at a level where nuclei are present in both, since in mosaic wing discs *Rp^+/-^* nuclei can be displaced basally compared to wild-type cells (eg Figure 1—figure supplement 1C, D).

### Reduced protein synthesis is due to PERK-dependent eIF2α phosphorylation in *Rp^+/-^* cells

**Figure 3.**
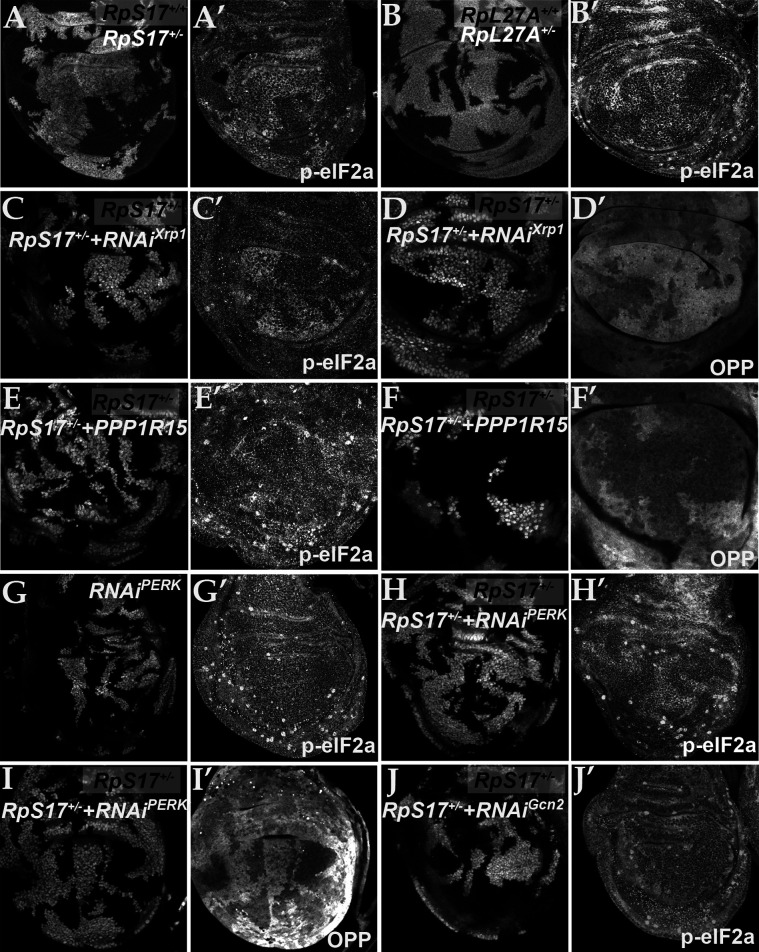
eIF2α is phosphorylated in ribosomal protein mutants via Xrp1 and PERK. Panels A-J show single confocal planes from third instar wing imaginal discs. (**A**) Mosaic of *RpS17*^+/-^ and *RpS17*^+/+^ cells. p-eIF2α levels were increased in *RpS17*^+/-^ cells (see A’). (**B**) Mosaic of *RpL27A*^+/-^ and *RpL27A*^+/+^ cells. p-eIF2α levels were increased in *RpL27A*^+/-^ cells (see B’). (**C**) Clones of cells expressing Xrp1-RNAi in a *RpS17*^+/-^ wing disc in white p-eIF2α levels were reduced by Xrp1 depletion (see C’). (**D**) Clones of cells expressing Xrp1-RNAi in a *RpS17*^+/-^ wing disc in white. Translation rate was restored by Xrp1 depletion (see D’). (**E**) Clones of cells over-expressing PPP1R15 in a *RpS17*^+/-^ wing disc in white. p-eIF2α levels were reduced by PPP1R15 over-expression (see E’). (**F**) Clones of cells over-expressing PPP1R15 in a *RpS17*^+/-^ wing disc in white. Translation rate was restored by PPP1R15 over-expression (see F’). (**G**) Clones of cells expressing PERK-RNAi in an otherwise wild type wing disc in white. p-eIF2α levels were unaffected (see G’). Note that in this and some other panels mitotic cells are visible near the apical epithelial surface. Mitotic figures, which lack OPP incorporation, are labeled by the anti-p- eIF2α antibody from Thermofisher, but not by the anti-p- eIF2α antibody from Cell Signaling Technologies. (**H**) Clones of cells expressing PERK-RNAi in a *RpS17*^+/-^ wing disc in whiite. p-eIF2α levels were reduced by PERK knockdown (see H’). (**I**) Clones of cells expressing PERK-RNAi in a *RpS17*^+/-^ wing disc in white. Translation rate was restored by PERK knockdown (see I’). (**J**) Clones of cells expressing Gcn2-RNAi in a *RpS17*^+/-^ wing disc in white. p-eIF2α levels were not reduced by Gcn2 knockdown (see J’). Further data relevant to this Figure are shown in Figure 3—figure supplement 1. Genotypes: A: p{hs:FLP}/+; *RpS17* p{arm:LacZ} FRT80B/FRT80B, B: p{hs:FLP}/ p{hs:FLP}; RpL27A^-^ p{arm:LacZ} FRT40/FRT40, C, D: p{hs:FLP}/+; *RpS17*, act> CD2> Gal4, UAS-GFP /UAS- RNAi^Xrp1^, E,F: p{hs:FLP}/+; UAS-*PPP1R15*/+; *RpS17*, act> CD2> Gal4, UAS-GFP /+, G: p{hs:FLP}/+; UAS- RNAi^PERK^ /+;act> CD2> Gal4, UAS-GFP /+, H, I: p{hs:FLP}/+; UAS- RNAiPERK /+; RpS17, act> CD2> Gal4, UAS-GFP /+, J: p{hs:FLP}/+; UAS- RNAi^Gcn2^/+; *RpS17*, act> CD2> Gal4, UAS-GFP /+.

*Rp* mutations may lead to surplus unused Rp. In yeast, aggregation of unused Rp rapidly affects specific transcription factors, leading to a transcriptional stress response (Albert et al., 2019; Tye et al., 2019). To explore how Xrp1 reduces translation, if not through reduced ribosome levels, we investigated the phosphorylation of eIF2α, a key mechanism of global regulation of CAP-dependent translation that responds to proteotoxic stress, among other influences (Hinnebusch and Lorsch, 2012). Strikingly, phosphorylation of eIF2α was increased in a cell-autonomous manner in *Rp^+/-^* cells compared to *Rp^+/+^* cells (RpS3, RpS17, RpS18, and RpL27A were examined) (Figure 3A and B; Figure 3—figure supplement 1A, B). Control clones lacking Rp mutations did not affect p-eIF2α levels or global translation rate (Figure 3—figure supplement 1M and N). Normal p-eIF2α levels were restored in *Rp^+/-^* cells, when even one copy of the *Xrp1* gene was mutated, as expected for the Xrp1-dependent process that reduces translation in *Rp^+/-^* cells (Figure 3—figure supplement 1C-E). To verify that eIF2α regulation by Xrp1 was cell-autonomous, we used clonal knockdown with an *Xrp1* dsRNA previously shown to restore normal growth to *Rp^+/-^* cells (Blanco et al., 2020). As predicted, *Xrp1* knockdown decreased eIF2α phosphorylation and increased translation rate in a cell-autonomous way (Figure 3C and D), as did knocking-down the gene encoding the Xrp1 heterodimer partner, Irbp18 (Francis et al., 2016; Blanco et al., 2020; Figure 3—figure supplement 1F, G).

**Figure 3—figure supplement 1.**
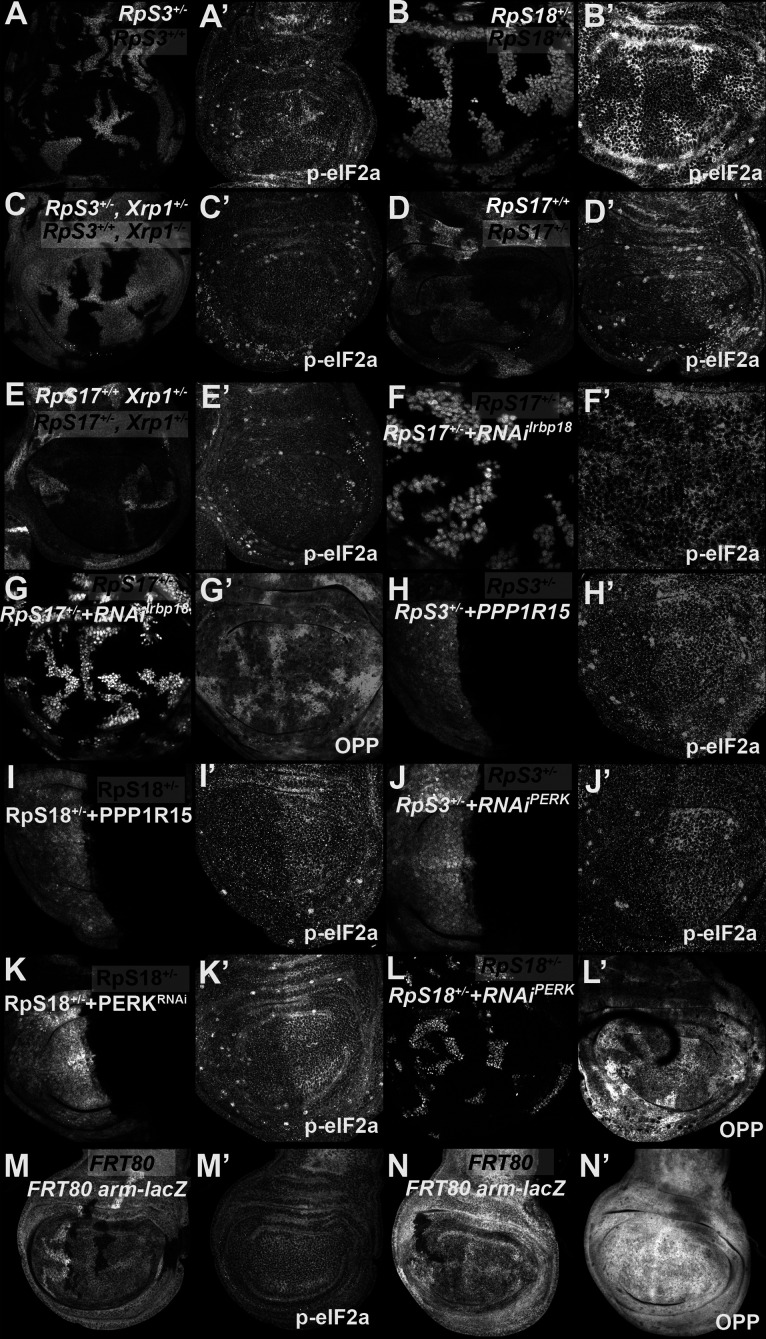
eIF2α phosphorylation in *Rp^+/-^* cell depends on Xrp1 and Irbp18. Panels A-L show single confocal planes from third instar wing imaginal discs. (**A**) Mosaic of *RpS3*^+/-^ and *RpS3*^+/+^ cells. p-eIF2α levels were increased in *RpS3*^+/-^ cells (see A’). (**B**) Mosaic of *RpS18*^+/-^ and *RpS18*^+/+^ cells. p-eIF2α levels were increased in *RpS18*^+/-^ cells (see B’). (**C**) Mosaic of *RpS3*^+/-^*Xrp1*+/-and *RpS3*^+/+^*Xrp^-/-^* cells. p-eIF2α levels were unaffected in *RpS3*^+/-^ cells when *Xrp1* was mutated (see C’). (**D**) Mosaic of *RpS17*^+/-^ and *RpS17*^+/+^ cells (the latter brighter white, having two copies of β-gal transgene). p-eIF2α levels were increased in *RpS17*^+/-^ cells (see D’). (**E**) Mosaic of *RpS17*^+/-^ and *RpS17*^+/+^ cells in an *Xrp1^+/-^* disc, (*RpS17*^+/+^ brighter white, having two copies of β-gal transgene). p-eIF2α levels were unaffected in *RpS17*^+/-^ cells (see E’). (**F**) Clones of cells expressing Irbp18 RNAi in a *RpS17*^+/-^ wing disc in white. p-eIF2α levels were reduced by Irbp18 knock-down (see F’). (**G**) Clones of cells expressing Irbp18 RNAi in a *RpS17*^+/-^ wing disc in white. Translation rate was restored by Irbp18 knock-down (see G’). (**H**) Cells over-expressing PPP1R15 in the posterior compartment of a *RpS3*^+/-^ wing disc in white. p-eIF2α levels were reduced by PPP1R15 over-expression (see H’). (**I**). Cells over-expressing PPP1R15 in the posterior compartment of a *RpS18*^+/-^ wing disc in white. p-eIF2α levels were reduced by PPP1R15 over-expression (see I’). (**J**) Cells expressing PERK RNAi in the posterior compartment of a *RpS3*^+/-^ wing disc in white. p-eIF2α levels were reduced by PERK knock-down (see J’). (**K**). Cells expressing PERK RNAi in the posterior compartment of a *RpS18*^+/-^ wing disc in white. p-eIF2α levels were reduced by PERK knock-down (see K’). (**L**) Clones of cells expressing PERK RNAi in a *RpS18*^+/-^ wing disc in white. Translation rate was restored by PERK knock-down (see L’). (**M**) Neutral clones expressing or lacking LacZ expression did not affect p-eIF2α levels (see M’). (**N**) Neutral clones expressing or lacking LacZ expression did not affect OPP incorporation (see N’). Genotypes: A: p{hs:FLP}/ p{hs:FLP}; FRT82 *RpS3* p{arm:LacZ} /FRT82B, B: p{hs:FLP}/ p{hs:FLP}; FRT42 *RpS18* p{Ubi:GFP}/FRT42, C: p{hs:FLP}/ p{hs:FLP}; FRT82 *RpS3* p{arm:LacZ} /FRT82B Xrp1^M2-73^, D: p{hs:FLP}/ p{hs:FLP}; *RpS17* FRT80B/p{arm:LacZ} FRT80B, E: p{hs:FLP}/ p{hs:FLP}; *RpS17* FRT80B/p{arm:LacZ} FRT80B *Xrp1^M2^*^–73^, F-G: p{hs:FLP}/+; *RpS17*, act> CD2> Gal4, UAS-GFP /UAS- RNAi*^Irbp18^,* H: en-GAL4, UAS-GFP /UAS-PPP1R15; FRT82 *RpS3*/+, I: *RpS18*^-^,en-GAL4, UAS-GFP /UAS-PPP1R15, J: en-GAL4, UAS-GFP / UAS- RNAi^PERK^; FRT82 *RpS3*/+, K: *RpS18*^-^,en-GAL4, UAS-GFP /UAS- RNAi^PERK^, L: p{hs:FLP}/+; UAS- RNAi^PERK^ / *RpS18*^-^; act> CD2> Gal4, UAS-His-RFP/+, M, N: p{hs:FLP}/ p{hs:FLP}; FRT80B/p{arm:LacZ} FRT80B.

If eIF2α phosphorylation is how Xrp1 reduces translation in *Rp^+/-^* cells, we expected translation to be restored by overexpressed PPP1R15, the *Drosophila* protein homologous to the mammalian p-eIF2α phosphatases, Gadd34 (PPP1R15a) and CReP (PPP1R15b) (Harding et al., 2009; Malzer et al., 2013). Indeed, PPP1R15 cell-autonomously reduced p-eIF2α levels and cell-autonomously restored overall translation levels in multiple *Rp* genotypes, as measured using the Click reagent *o*-propargyl puromycin (OPP) (Figure 3E and F; Figure 3—figure supplement 1H, I). These data indicate that it is eIF2α phosphorylation that suppresses translation in *Rp^+/-^* cells.

*Drosophila* contains two potential eIF2α kinases that are thought to respond to particular stresses and not to be activated in unstressed epithelial wing disc cells. When protein kinase R-like endoplasmic reticulum (ER) kinase (PERK), a kinase that phosphorylates eIF2α during the unfolded protein response (Shi et al., 1998; Harding et al., 1999; Harding et al., 2000; Pakos-Zebrucka et al., 2016), was depleted using RNAi, p-eIF2α levels were unaffected in wild type wing discs (Figure 3G). By contrast, in the *Rp^+/-^* genotypes the levels of p-eIF2α were reduced by PERK depletion (Figure 3H; Figure 3—figure supplement 1J, K). Thus, PERK activity was higher in *Rp^+/-^* cells and responsible for eIF2α phosphorylation there. Consistently, PERK knock-down cell-autonomously restored normal translation levels in multiple *Rp^+/-^* genotypes (Figure 3I; Figure 3—figure supplement 1L). Depletion of the other eIF2α kinase known in *Drosophila*, Gcn2, did not decrease p-eIF2α levels in *Rp^+/-^* wing disc cells (Figure 3J).

### Xrp1 increases protein aggregation and modifies UPR gene expression in *RpS^+/-^* cells

**Figure 4.**
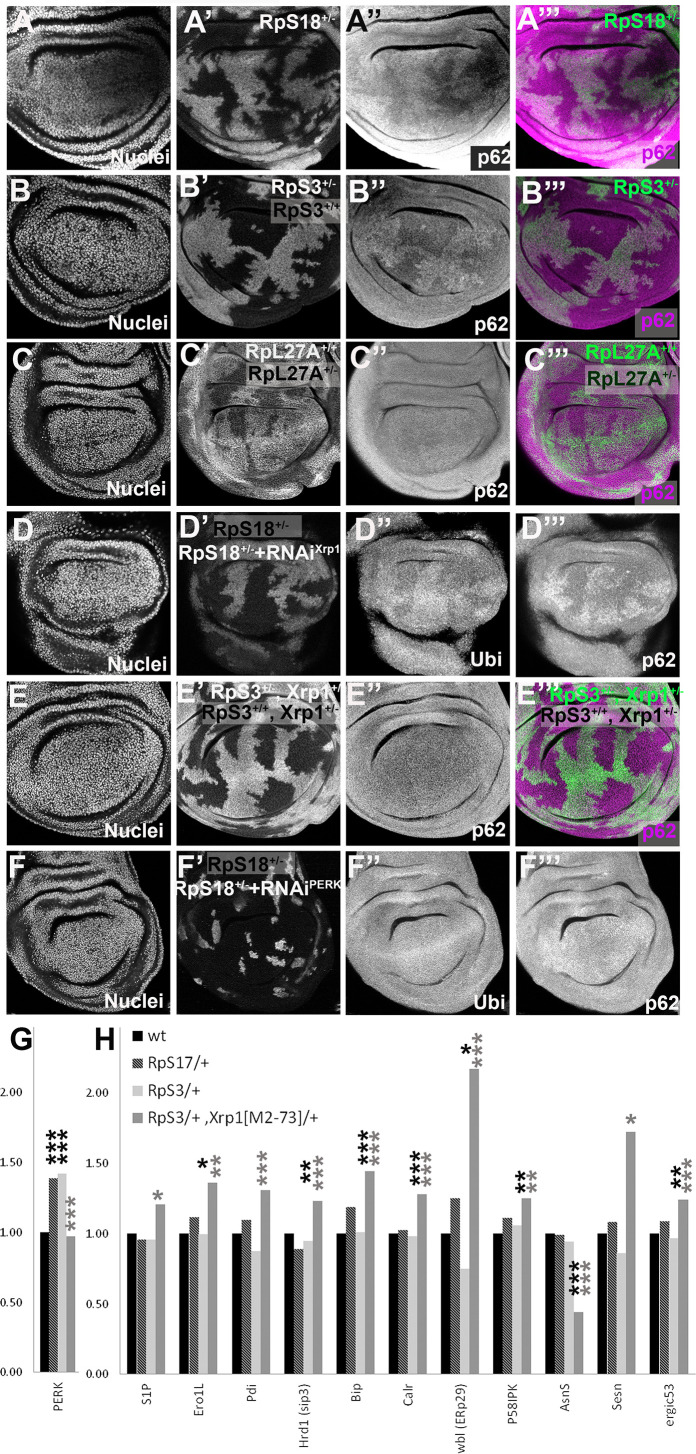
Xrp1-dependent aggregates and gene expression changes in *RpS*^+/-^ cells. Panels A-E show single confocal planes from third instar wing imaginal discs, mosaic for the genotypes indicated. In all cases, the plane passes through the central nuclei-containing disk portion for the genotypes shown. (**A**) p62 was higher in *RpS18^+/-^* cells than *RpS18^+/+^* cells. (**B**) p62 was higher in *RpS3^+/-^* cells than *RpS3^+/-^* cells. (**C**) p62 was comparable in *RpL27A^+/- ^*cells and *RpL27A^+/+^* cells. (**D**) Clones of cells expressing Xrp1-RNAi in a *RpS18*^+/-^ wing disc in white. Levels of both p62 and ubiquitinylated proteins were reduced by Xrp1 knock-down. (**E**) Mosaic of *RpS3*^+/-^ and *RpS3*^+/+^ cells in *Xrp1^+/-^* wing disc. No increase in p62 was seen in *RpS3*^+/-^ cells (compare panel B). (**F**) Clones of cells expressing PERK-RNAi in a *RpS18*^+/-^ wing disc in white. Levels of both p62 and ubiquitinylated proteins remained unaffected by PERK knock-down (**G**). PERK mRNA levels (fold changes in mRNA-seq replicates relative to the wild-type controls according to Deseq2) for the indicated genotypes. PERK mRNA was increased in both *RpS17*^+/-^ and *RpS3*^+/-^ wing discs but not *RpS3*^+/-^, *Xrp1*^M2-73/+^ cells. (**H**) mRNA levels for 11 genes participating in the Unfolded Protein Response. All were significantly affected only in the *RpS3^+/-^ Xrp1 ^M2-73/+^* genotype. Statistics: Asterisks indicate statistical significance determined by Deseq2 (*: p_adj_ <0.05, **: p_adj_ <0.005, ***: p_adj_ <0.0005) compared to wild type control (black asterisks) or to *RpS3*^+/-^ genotype (grey asterisks). Comparisons not indicated were not significant ie p_adj_ ≥0.05 eg *PERK* mRNA in *RpS3^+/-^ Xrp1 ^M2-73/+^* compared to wild type control. Further data relevant to this Figure are shown in Figure 4—figure supplement 1. Data are based on mRNA-sequencing of 3 biological replicates for each genotype. Genotypes: A: p{hs:FLP}/ p{hs:FLP}; FRT42 *RpS18* p{Ubi:GFP}/FRT42, B: p{hs:FLP}/ p{hs:FLP}; FRT82 *RpS3* p{arm:LacZ} /FRT82B, C: p{hs:FLP}/ p{hs:FLP}; *RpL27A^-^* p{arm:LacZ} FRT40/FRT40, D: p{hs:FLP}/+; UAS- RNAi^Xrp1^/ GstD lacZ, *RpS18*^-^; act> CD2> Gal4, UAS-GFP /+, E: p{hs:FLP}/ p{hs:FLP}; FRT82 RpS3 p{arm:LacZ} /FRT82B Xrp1^M2-73^, F: p{hs:FLP}/+; UAS- RNAi^PERK^ / GstD-lacZ, *RpS18*^-^; act> CD2> Gal4, UAS-GFP /+,G-H: wt: w ^11-18^ /+; FRT82B/+, RpS17/+: w^11-18^ /y w p{hs:FLP}; *RpS17* p{ubi:GFP} FRT80B/+, RpS3/+: w^11-18^ /y w p{hs:FLP}; FRT82 *RpS3* p{arm:LacZ/+, *RpS3*/+, *Xrp1*^[M2-73]^/+: w^11-18^ /y w p{hs:FLP}; FRT82 *RpS3* p{arm:LacZ}/ FRT82B *Xrp1^M2^*^–73^.

Recently, protein aggregates have been detected in the cytoplasm of wing disc cells heterozygous for *RpS3*, *RpS23*, and *RpS26* mutants, as foci of ubiquitin or p62 accumulation, reflecting decreased proteasome activity and autophagy (Baumgartner et al., 2021; Recasens-Alvarez et al., 2021). We confirmed the greater accumulation of aggregates in *RpS3^+/-^*and *RpS18^+/-^*cells compared to wild type cells but did not see this for *RpL27A^+/-^* cells (Figure 4A–C). Significantly, another study saw no general increase in autophagy in *RpL14^+/-^* wing discs (Nagata et al., 2019). It would be interesting to examine more mutants affecting the LSU, to see whether autophagy is generally unaffected by *RpL* mutations. Importantly, aggregates in *RpS3^+/-^*and *RpS18^+/-^* wing discs were Xrp1-dependent, placing them downstream of Xrp1 activation (Figure 4D–E).

**Figure 4—figure supplement 1.**
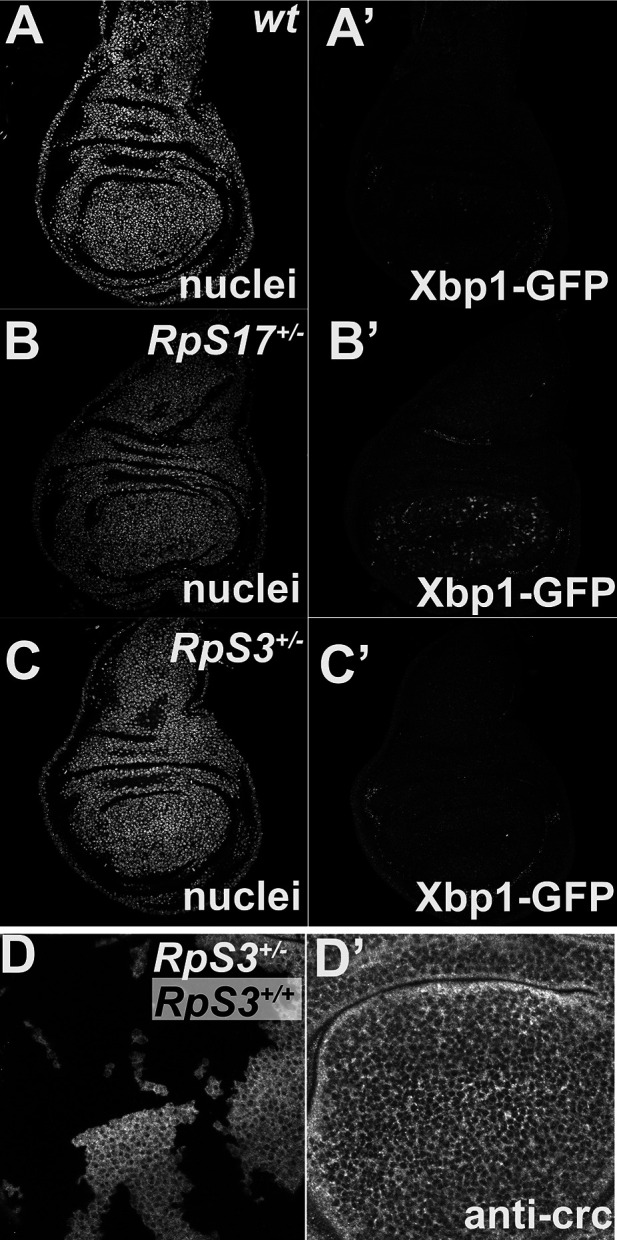
Little UPR detected in *Rp^+/-^* wing discs. Panels show single confocal planes from third instar wing imaginal discs expressing the UPR reporter UAS-Xbp1-GFP in the wing pouch under *nub-Gal4* control. GFP expression indicates an unfolded protein response. (**A**) Little evidence for UPR in wild type wing discs. (**B**) Elevated UPR in *RpS17^+/-^* wing discs. (**C**) Little evidence for UPR in *RpS3^+/-^* wing discs. (**D**) No upregulation of Crc/ATF4 in *RpS3^+/-^* cells. Genotypes: A: Xbp1-EGFP/nubGal4; +/+, B: Xbp1-EGFP/nubGal4; *RpS17* p{arm:LacZ} FRT80B /+, C: Xbp1-EGFP/nubGal4; FRT82 *RpS3* p{arm:LacZ} /+ D: p{hs:FLP}/ p{hs:FLP}; FRT82 *RpS3* p{arm:LacZ} /FRT82B.

PERK is a transmembrane protein with a cytoplasmic kinase domain that is a sensor of unfolded proteins within the ER, not within the cytoplasm or nucleolus ( Bertolotti et al., 2000; Harding et al., 2000; Ron and Walter, 2007; Walter and Ron, 2011). Cytoplasmic aggregates can cause unfolded protein accumulation within the ER by competing for proteasomes, however. ER stress also activates Ire-1 and Atf6 in parallel to PERK (Bertolotti et al., 2000; Ron and Walter, 2007; Walter and Ron, 2011; Hetz, 2012; Mitra and Ryoo, 2019). Xbp1-GFP (Sone et al., 2013; Mitra and Ryoo, 2019), a reporter for Ire-1 activity, was only inconsistently activated in *Rp^+/-^* wing discs (Figure 4—figure supplement 1A, C), in agreement with the absence of any transcriptional signature of Atf6 or Xbp1 activation in *Rp*^+/-^ wing disc mRNA-seq data (Lee et al., 2018). Crc/Atf4 protein was not upregulated in RpS3^+/-^ cells, which would be expected in the classic PERK/ATF4 branch activation of UPR (Figure 4—figure supplement 1D). PERK mRNA levels were elevated by 1.4 x in both *RpS3^+/-^* and *RpS17^+/-^* wing discs, however (Figure 4G). This increase was statistically very significant, replicated in another group’s data, and entirely dependent on *Xrp1* (Figure 4G; Kucinski et al., 2017; Lee et al., 2018). *BiP* and 10 other UPR genes were affected differently. Although none were significantly altered in *RpS17^+/-^* or *RpS3^+/-^* discs, all these genes were affected in *RpS3^+/-^ Xrp1^+/- ^*wing discs, suggesting that Xrp1 prevents their elevation in *RpS17^+/-^* or *RpS3^+/-^* discs (Figure 4H). Since these genes help restore ER proteostasis (Walter and Ron, 2011), we speculate that Xrp1 might sensitize *Rp^+/-^* cells to PERK activation relative to Atf6 or Xbp1 branches of the UPR (Lin et al., 2007), by elevating the expression of PERK while blunting the usual proteostatic response. Testing this notion would require manipulating multiple genes in vivo simultaneously.

### eIF2α phosphorylation is sufficient to induce competitive apoptosis, but through Xrp1

**Figure 5.**
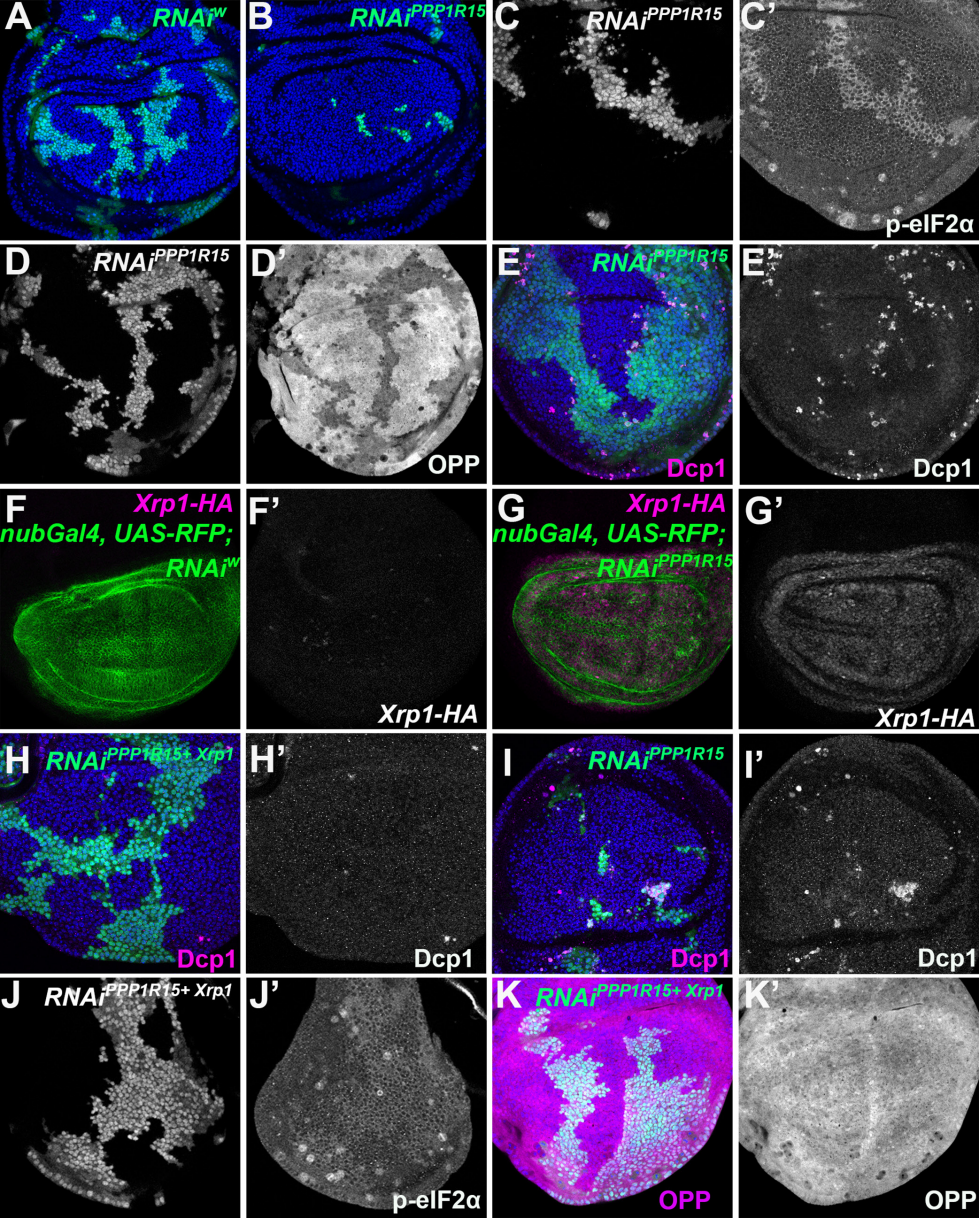
eIF2α phosphorylation can induce Xrp1 expression and cell competition. All panels show single confocal planes from third instar wing imaginal discs, mosaic for the genotypes indicated. All the sections pass through the central region of the disc proper containing nuclei in all genotypes, as indicated by the DNA stain in blue in some panels. (**A**) Clones expressing *white* RNAi (green). Clones induced by 7 min heat shock. (**B**) Clones expressing *PPP1R15* RNAi (green)were fewer and smaller than the control (compare panel A). Clones induced by 7 min heat shock. (**C**) Clones expressing *PPP1R15* RNAi (white) contain phosphorylated eIF2α (see C’). (**D**) Clones induced by 25 ± 5 min heat shock, which results in larger clone areas (white). Labelled clones expressing *PPP1R15* RNAi reduced translation rate (see D’). (**E**) Labelled clones expressing *PPP1R15* RNAi (green) underwent competitive apoptosis at interfaces with wild type cells (activated caspase Dcp1 in magenta; see also E’). (**F**) *Nub-Gal4* drives gene expression in the wing pouch, shown in green for RFP, with little expression of Xrp1-HA (magenta; see also F’). (**G**) *PPP1R15* RNAi induces Xrp1-HA expression in the wing pouch (magenta; see also G’). (**H**) Clones co-expressing *PPP1R15* RNAi and *Xrp1* RNAi (green) lacked competitive apoptosis (activated caspase Dcp1 in magenta; see also H’). (**I**) Clones expressing *PPP1R15* RNAi (green). Experiment performed in parallel to panel H. Note competitive apoptosis at interfaces with wild type cells (activated caspase Dcp1 in magenta; see also I’), and smaller clone size. Cell death at the basal surface of the same disc shown in Figure 5—figure supplement 1F. (**J**) Clones co-expressing *PPP1R15* RNAi and *Xrp1* RNAi (white) showed less eIF2α phosphorylation than for *PPP1R15* RNAi alone (compare panel C). Sample prepared in parallel to panel C (in the same tube from fixation to staining). (**K**) *Xrp1* knock-down restored normal translation rate to cell clones expressing *PPP1R15* RNAi (green; see also K’). Sample prepared in parallel to panel D (in the same tube from fixation to staining). Additional data relevant to this Figure is shown in Figure 5—figure supplement 1. Genotypes: A: {hs:FLP}/+; act> CD2> Gal4, UAS-GFP / UAS – RNAi^w^, B: {hs:FLP}/+; act> CD2> Gal4, UAS-GFP / UAS – RNAi^PPP1R15^ (line: BL 33011) (samples were processed on the same day, not on the same tube), C: {hs:FLP}/+; UAS – RNAi^PPP1R15^ /TRE-dsRed; act> CD2> Gal4, UAS-GFP /+(line: v107545) (processed in parallel with 5 J), D: {hs:FLP}/+; act> CD2> Gal4, UAS-GFP / UAS – RNAi^PPP1R15^ (line: BL 33011), E: {hs:FLP}/+; UAS – RNAi^PPP1R15^ /+; act> CD2> Gal4, UAS-GFP /+ (line: v107545),F: nubGal4, UAS-RFP/+; Xrp1-HA/RNAi^w^, G: nubGal4, UAS-RFP/ UAS – RNAi^PPP1R15^; Xrp1-HA/+ (line: v107545), H, J, K: {hs:FLP}/+; UAS – RNAi^PPP1R15^ / UAS-RNAi^Xrp1^; act> CD2> Gal4, UAS-GFP /+ (line: v107545) (5 H processed in parallel with 5I. Also, 5 K processed in parallel with Figure 5—figure supplement 1B) (line RNAi^PPP1R15^: v107545 and line RNAi^Xrp1^: v107860), I: {hs:FLP}/+; UAS – RNAi^PPP1R15^ /TRE-dsRed; act> CD2> Gal4, UAS-GFP /+ (line RNAi^PPP1R15^: v107545).

We determined whether manipulating p-eIF2α levels alone was sufficient to cause competition of otherwise wild-type cells. Consistent with this notion, clones of cells depleted for PPP1R15 were rapidly lost from wing imaginal discs and could rarely be recovered (Figure 5A and B). Under some conditions (longer heat shock) where clones of cells depleted for PPP1R15 survived temporarily, we verified that p-eIF2α was increased and translation reduced compared to nearby wild-type cells (Figure 5C and D; Figure 5—figure supplement 1A, B). Such surviving clones were characterized by apoptosis of PPP1R15-depleted cells predominantly at the interface with wild-type cells, a sign of cell competition (Figure 5E; Figure 5—figure supplement 1C).

**Figure 5—figure supplement 1.**
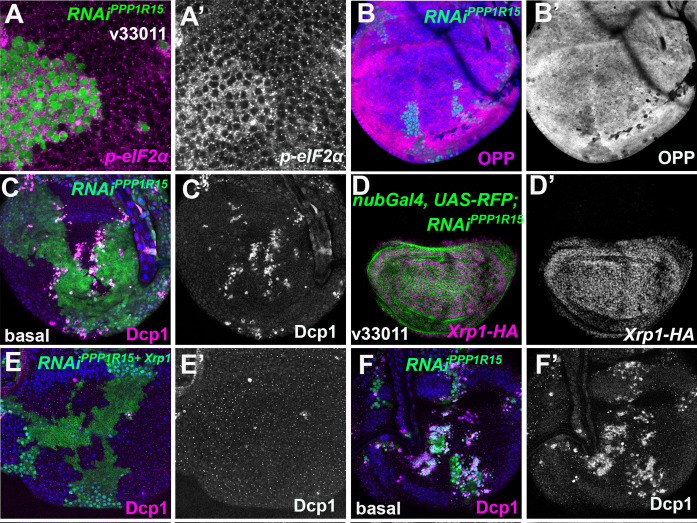
eIF2α phosphorylation induces Xrp1 expression and cell competition. Single confocal planes from third instar wing imaginal discs. (**A**) Clones expressing PPP1R15-RNAi had increased p-eIF2α levels (**A’**). (**B**) Clones expressing PPP1R15-RNAi had reduced translation (OPP) (**B’**). (**C**) Basal section of the same disc shown in Figure 5E. More dying PPP1R15-RNAi cells labeled for active caspase (magenta, see also C’) accumulate basally at the boundaries with the wild-type cells. (**D**) *nub-Gal4* driving *PPP1R15* RNAi in the wing pouch (green) led to Xrp1-HA expression (magenta; see also D’). (**E**) Basal confocal section of the wing disc also shown in Figure 5H, mosaic for cells expressing *PPP1R15* RNAi and Xrp1 RNAi (green). Even at these basal levels, apoptosis was almost completely rescued by Xrp1 knockdown (magenta, see also E’). (**F**) Basal confocal section of wing disc mosaic for wild-type cells and cells expressing *PPP1R15* RNAi also shown in Figure 5I and a parallel control to panel E. Note extensive cell death basally (magenta, see also F’), as well as smaller clone size (green). Genotypes: A: {hs:FLP}/+; act> CD2> Gal4, UAS-GFP / UAS – RNAi^PPP1R15^ (line: BL 33011), B: {hs:FLP}/+; UAS – RNAi^PPP1R15^ /+; act> CD2> Gal4, UAS-GFP /+ (line: v107545) (processed in parallel with Figure 5K), C: {hs:FLP}/+; UAS – RNAi^PPP1R15^ /+; act> CD2> Gal4, UAS-GFP /+ (line: v107545) (basal side of the same disc in Figure 5E), D: nubGal4, UAS-RFP/ UAS – RNAi^PPP1R15^; Xrp1-HA/+ (line: Bl 33011), E: {hs:FLP}/+; UAS – RNAi^PPP1R15^ /+; act> CD2> Gal4, UAS-GFP /+ (line: v107545) (basal side of the same disc in Figure 5H), F: {hs:FLP}/+; UAS – RNAi^PPP1R15^ /UAS-RNAi^Xrp1^; act> CD2> Gal4, UAS-GFP /+ (line RNAi^PPP1R15^: v107545 and line RNAi^Xrp1^: v107860) (basal side of the same disc in Figure 5I).

**Figure 5—figure supplement 2.**
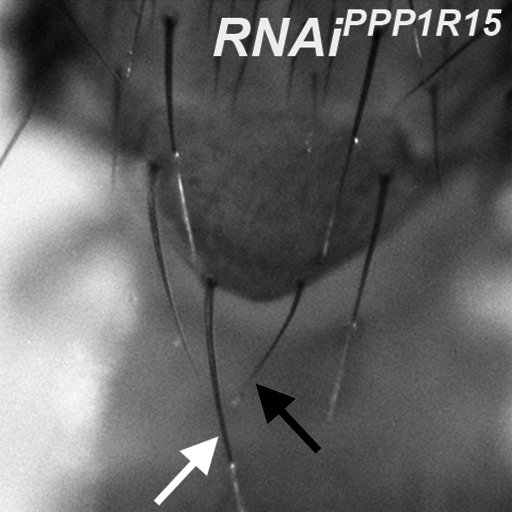
eIF2α phosphorylation reduces bristle size. Minute-like short, thin bristles occur on adults containing clones depleted for PPP1R15, for example compare highlighted posterior scutellar bristle (black arrow) with normal contralateral bristle (white arrow). Genotype: p{hs:FLP}/+; UAS- RNAi ^PPP1R15^/+;act> CD2> Gal4, UAS- GFP /+ (line: Bl 33011).

**Figure 5—figure supplement 3.**
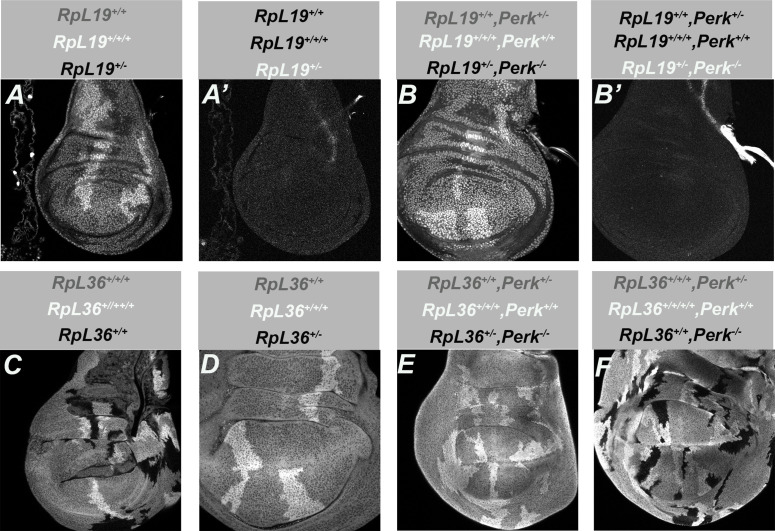
eIF2α phosphorylation is not required for cell competition. All panels show single confocal planes from third instar wing imaginal discs, mosaic for the genotypes indicated. (**A**) When mitotic recombination generated twin clones of *RpL19^+/+/+^* cells (brightly labeled) and *RpL19^+/-^* ± (black) in the *RpL19^+/+^* background (gray), the *RpL19^+/-^* ± did not survive. This was also shown by the absence of mCherry expression, which would have been labelled white in panel A’. (**B**) Clones of *perk^-/-^ RpL19^+/-^* ± (black) were also not seen after recombination in the *perk^+/-^ RpL19^+/+^* background (gray) led to twin clones of *perk^+/+^ RpL19^+/+/+^* cells (brightly labeled). Any clones of *perk^-/-^ RpL19^+/-^* ± would also have been detected through mCherry expression, which would have been labelled white in panel B’. (**C**) Clones of both *RpL36^+/+/+/+^* cells (brightly labeled) and *RpL36^+/+^* cells (black) survived in the *RpL36^+/+/+^* background (gray). (**D**) No clones of *RpL36^+/-^* ± (black) were seen after recombination in the *RpL36^+/+^* background (gray) generated twin clones of *RpL36^+/+/+^* cells (brightly labeled). (**E**) No clones of *perk^-/-^ RpL36^+/-^* ± (black) were seen after recombination in the *perk^+-/-^ RpL36^+/+^* background (gray) generated twin clones of *perk^+/+^ RpL36^+/+/+^* cells (brightly labeled). (**F**) Clones of *perk^-/-^ RpL36^+/+^* cells (black) were readily obtained after recombination in the *perk^+-/-^ RpL36^+/+/+^* background (gray) generated twin clones of *perk^+/+^ RpL36^+/+/+/+^* cells (brightly labeled). Genotypes: A,A’: y w, hs-FLP; Act5C > GAL4 w, UAS-mCherry-CAAX, Df(2 R)M60E; FRT82B, ubi-GFP-nls, tubP-GAL80, M{RpL19 genomic}ZH-86Fb/FRT82B. B,B’: y w, hs-FLP; Act5C > GAL4 w, UAS-mCherry-CAAX, Df(2 R)M60E; FRT82B, ubi-GFP-nls, tubP-GAL80, M{RpL19 genomic}ZH-86Fb/FRT82B Perk^null^ C: FM7/hs-FLP; FRT82B p{RpL36^+^} p{arm:LacZ}/FRT82B. D: Df(1)R194/hs-FLP; FRT82B p{RpL36^+^} p{arm:LacZ}/FRT82B. E: Df(1)R194/hs-FLP; FRT82B p{RpL36^+^} p{arm:LacZ}/FRT82B PERK^null^ F: FM7/hs-FLP; FRT82B p{RpL36^+^} p{arm:LacZ}/FRT82B PERK^null^ The FM7 and Df(1)R194 genotypes used in panels C-F were unmarked in wing discs. The experiment with FRT82B led to the two classes of discs with and without clones shown in panels C and D, and the experiment with FRT82B *perk^-^* led to the two classes of discs with and without clones shown in panels E and F. A parallel experiment with FRT82B *Xrp1^-^*, which rescues *RpL36^+/-^* ± (Lee et al., 2018) led only to wing discs with clones equal in size and frequency to reciprocal twin clones.

If eIF2α phosphorylation was the downstream effector of Xrp1 that triggers cell competition in *Rp*^+/-^ cells then PPP1R15 depletion should eliminate cells independently of Xrp1. Like *Rp^+/-^* cells; however, PPP1R15-depleted cells showed strong upregulation of Xrp1 protein (Figure 5F and G; Figure 5—figure supplement 1D). When Xrp1 was knocked-down in PPP1R15-depleted cells, competitive cell death was completely blocked, and clone survival improved (Figure H-I; Figure 5—figure supplement 1E-F). Even the p-eIF2α levels in the PPP1R15 depleted clones partially depended on Xrp1 (compare Figure 5C with Figure 5J), and translation rates were similar to wild-type levels in PPP1R15 clones lacking Xrp1 (Figure 5K). Interestingly, PPP1R15 knock-down reduced bristle size, another similarity with *Rp* mutants (Figure 5—figure supplement 2).

**Figure 6.**
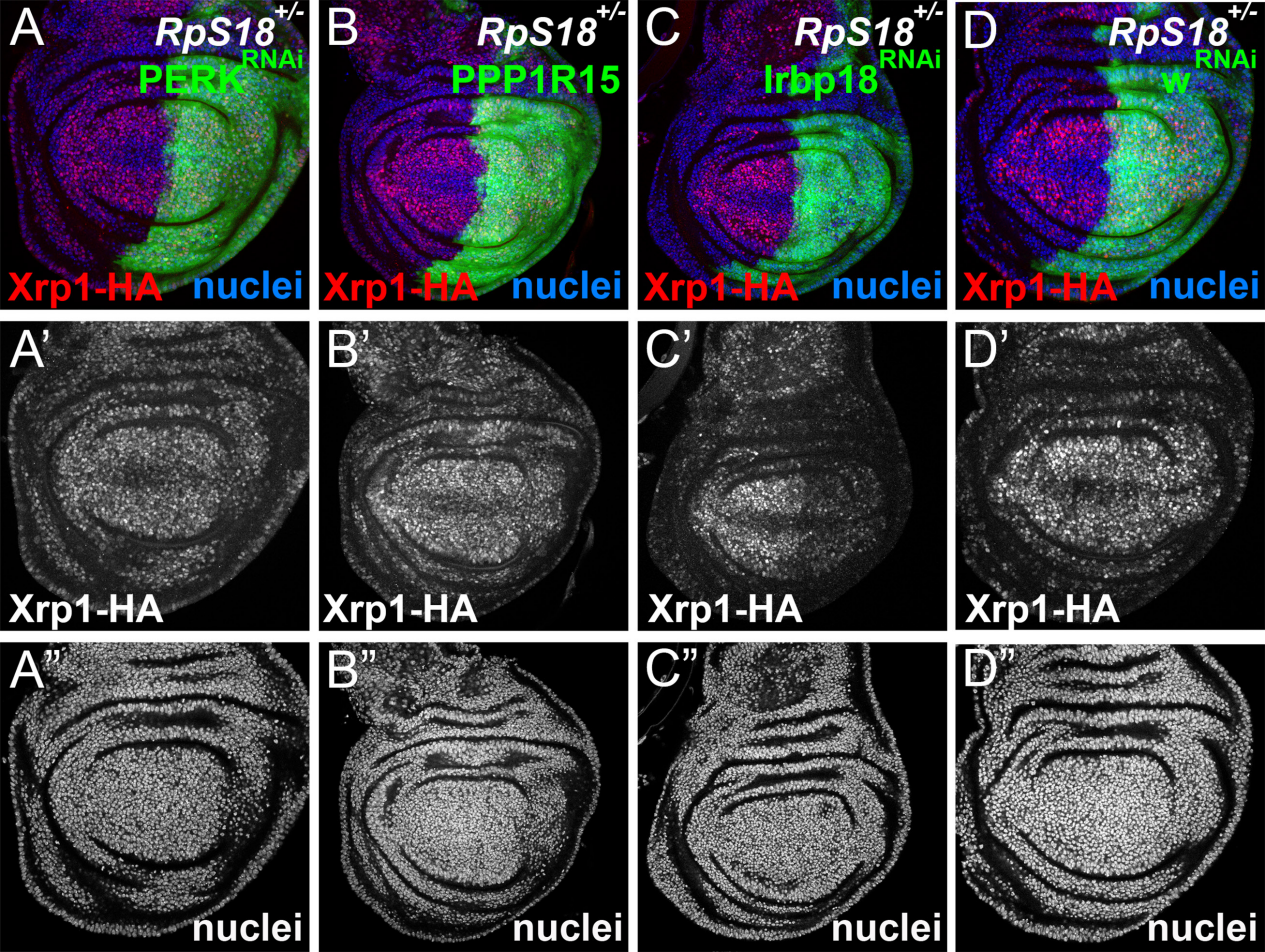
eIF2α phosphorylation is dispensable for Xrp1 expression in Minutes. All panels show single confocal planes from *RpS18^+/-^* third instar wing imaginal discs, co-expressing GFP and the indicated constructs in the posterior compartments. All the sections pass through the central region of the disc proper containing nuclei, as indicated by the DNA stain in blue. (**A**) Perk knock-down had no effect on Xrp1 expression in *RpS18^+/-^*. (**B**) PPP1R15 over-expression had no effect on Xrp1 expression in *RpS18^+/-^*. (**C**) Irbp18 knock-down strongly reduced Xrp1 expression in *RpS18^+/-^*. (**D**) Knock-down for the *w* gene had no effect on Xrp1 expression in *RpS18^+/-^*. Genotypes: A: *RpS18*^-^, en-GAL4, UAS-GFP / UAS- RNAi^PERK^; Xrp1-HA /+, B: *RpS18*^-^,en-GAL4, UAS-GFP / UAS-PPP1R15; Xrp1-HA /+, C: *RpS18*^-^,en-GAL4, UAS-GFP /+; Xrp1-HA /UAS- RNAi*^Irbp18^*, D:*RpS18*^-^,en-GAL4, UAS-GFP /+; Xrp1-HA /UAS- RNAi*^w^*.

These data raised the possibility of positive feedback between Xrp1 expression and eIF2α phosphorylation. To assess this, we compared Xrp1 expression in *RpS18^+/-^* cells with or without PERK RNAi or PPP1R15 over-expression (Figure 6A–B), each of which reduces eIF2α phosphorylation to or below baseline levels (Figure 3—figure supplement 1H-K). Because Xrp1 protein levels were unaffected, we concluded that while eIF2α phosphorylation was sufficient to promote Xrp1 expression in otherwise wild-type cells, it was not necessary for the Xrp1 protein expression seen in *RpS18^+/-^* cells (Figure 6). This continued Xrp1 expression was functional, because none of Xrp1-dependent JnK activity in *RpS17^+/-^* cells, Xrp1-dependent GstD-LacZ reporter activity in *RpS18^+/-^* cells, or Xrp1-dependent ubiquitin and p62 foci in *RpS18^+/-^* cells were affected by Perk knock-down (Figure 4, Figure 6—figure supplements 1 and 2). Xrp1 protein levels were reduced by knockdown of its heterodimer partner, Irbp18, in *Rp^+/-^* cells (Figure 6C), however. These findings indicate that *Rp^+/-^* cells activate Xrp1 expression independently of eIF2α phosphorylation. Positive feedback between Xrp1 expression and eIF2α phosphorylation might still be important under some circumstances, for example when PPP1R15 is knocked-down, where the effects on global translation and on cell competition depended on Xrp1 (Figure 5C–E and H–K).

**Figure 6—figure supplement 1.**
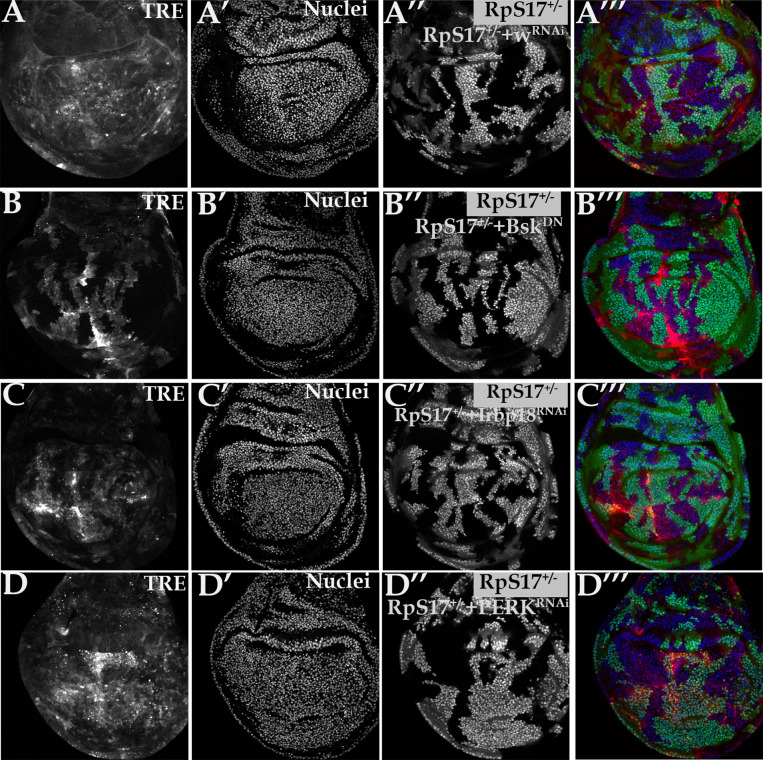
PERK is dispensable for Xrp1-dependent JnK activity in Minute cells. All panels show single confocal planes from *RpS17^+/-^* third instar wing imaginal discs, co-expressing GFP and the indicated constructs in the green-labeled clones. All the sections pass through the central region of the disc proper containing nuclei, as indicated by the DNA stain. (**A**) Clonal knockdown of the *w* gene did not affect TRE-dsRed, a reporter for JNK activity; (**B**) Bsk^DN^ expression extinguished TRE-dsRed from cell clones, confirming JNK-dependence of this reporter; (**C**) Irbp18 knock-down extinguished TRE-dsRed from cell clones, confirming Xrp1/Irbp18-dependence of this reporter, as previously concluded from non-clonal experiments (Lee et al., 2018); (**D**) PERK knock-down had little effect on TRE-dsRed, suggested that JNK is activated by Xrp1/Irbp18 independently of eIF2α phosphorylation. Genotypes: A: p{hs:FLP}/+; TRE-dsRed/+; *RpS17*, act> CD2> Gal4, UAS-GFP /UAS- RNAi*^w^,* B: p{hs:FLP}/+; TRE-dsRed/+; *RpS17*, act> CD2> Gal4, UAS-GFP /UAS- Bsk^DN^, C: p{hs:FLP}/+; TRE-dsRed/+; *RpS17*, act> CD2> Gal4, UAS-GFP /UAS- RNAi*^Irbp18^,* D: p{hs:FLP}/+; TRE-dsRed/ UAS- RNAi*^PERK^; RpS17*, act> CD2> Gal4, UAS-GFP /+*.*

**Figure 6—figure supplement 2.**
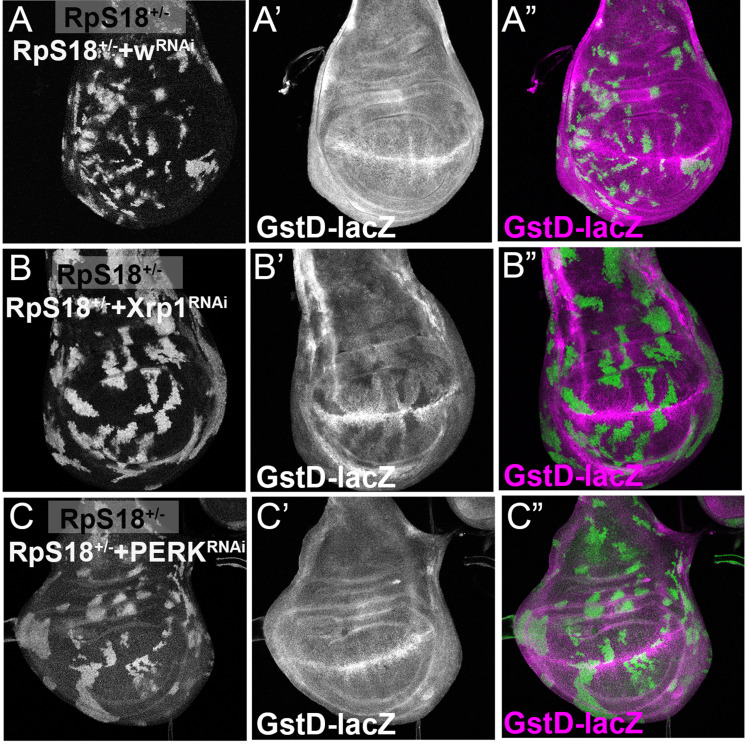
PERK is dispensable for Xrp1-dependent GstD1 activity in Minute cells. All panels show single confocal planes from *RpS18^+/-^* third instar wing imaginal discs, co-expressing GFP and the indicated constructs in the green-labeled clones. (**A**) Clonal knockdown of the *w* gene did not affect GstD1-LacZ, a reporter for oxidative stress response; (**B**) Xrp1 knock-down extinguished GstD1-LacZ from cell clones, confirming Xrp1-dependence of this reporter, as seen in non-clonal experiments (Lee et al., 2018); (**C**) PERK knock-down had no effect on GstD1-LacZ, suggested that Xrp1 regulates GstD1-LacZ independently of eIF2α phosphorylation. Genotypes: A: p{hs:FLP}/+;GstD1-lacZ, *RpS18*^-^/+; act> CD2> Gal4, UAS-GFP / UAS-RNAi^w^, B: p{hs:FLP}/+; UAS- RNAi^Xrp1^/ GstD1 lacZ, *RpS18*^-^; act> CD2> Gal4, UAS-GFP/+, C: p{hs:FLP}/+; UAS- RNAi^PERK^ / GstD1-lacZ, *RpS18*^-^; act> CD2> Gal4, UAS-GFP/+.

We also tested whether increased eIF2α phosphorylation was necessary for cell competition (Figure 5—figure supplement 3). We used assays where mitotic recombination generates clones of *RpL19^+/-^* cells or clones of *RpL36^+/-^*, both subject to competition by surrounding cells (Figure 5—figure supplement 3A, D; Tyler et al., 2007; Baillon et al., 2018). Since PERK was responsible for increasing eIF2α phosphorylation, we expected that if this was required for cell competition, then a *perk* null mutation should rescue the elimination *RpL19^+/-^* or *RpL36^+/-^* clones. Since no *RpL19^+/-^ perk^-/-^* or *RpL36^+/-^ Perk^-/-^* clones were recovered (Figure 5—figure supplement 3B, E), although *RpL36^+/+^ perk^-/-^*clones survived normally (Figure 5—figure supplement 3C, F), we concluded that PERK-dependent eIF2α phosphorylation was not required for cell competition.

These data show that eIF2α phosphorylation was sufficient to reduce cell competitiveness in otherwise wild type cells, but only in the presence of Xrp1. It was the mechanism whereby Xrp1 reduced global translation rate in *Rp^+/-^* mutant cells, but apparently not the downstream effector of Xrp1 for cell competition.

### Interrupting the translation cycle activates Xrp1-dependent cell competition, independently of diminished translation

Phosphorylation of eIF2α inhibits CAP-dependent initiation. To explore further whether reduced translation was sufficient to cause cell competition, we also reduced translation by clonal depletion of translation factors acting at a variety of steps in the translation cycle, not only at initiation but also the 40 S-60S subunit joining and elongation steps (Jackson et al., 2010). Specifically, we depleted eIF4G, eIF5A, eIF6, eEF1α1, and eEF2, none of which is encoded by a haploinsufficient gene (Marygold et al., 2007). eIF4G is part of the eIF4 complex which binds the mRNA 5’cap and recruits SSU to enable translation initiation (Jackson et al., 2010). It is now accepted that eIF5A functions in translation elongation and termination (Saini et al., 2009; Schuller et al., 2017). eEF1α1 delivers aminoacyl-tRNAs to the ribosome and eEF2 also promotes ribosome translocation (Dever and Green, 2012). eIF6 has a role during LSU biogenesis and also in translation initiation (Brina et al., 2015).

**Figure 7.**
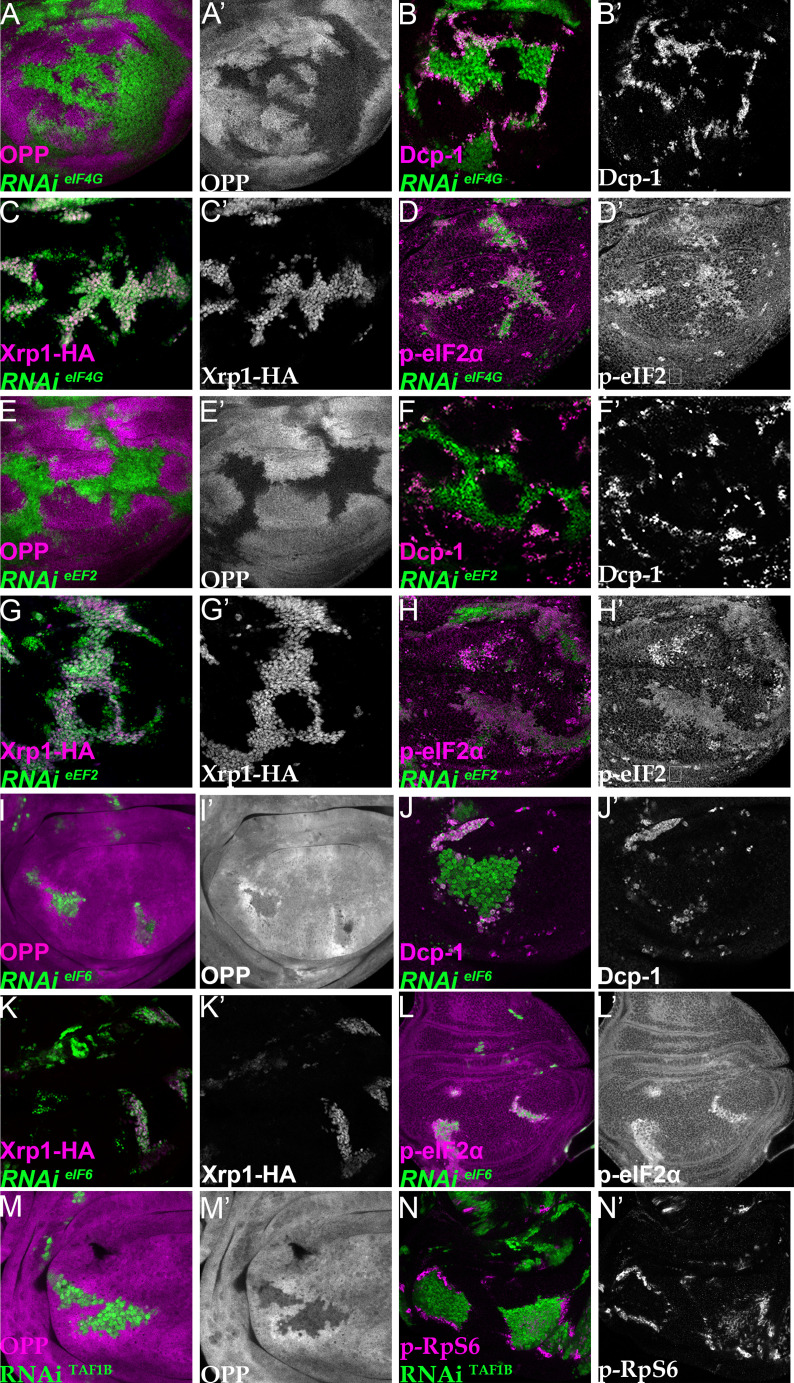
Depletion of translation factors induces Xrp1 expression, eIF2α phosphorylation, reduced translation, and cell competition. Clones of cells depleted for translation factors are labelled in green. In each case, translation factor depletion reduced translation rate, resulted in competitive cell death at interfaces with wild type cells, induced Xrp1-HA expression, and led to eIF2α phosphorylation. Translation rate, dying cells (activated caspase Dcp1), Xrp1-HA and p-eIF2α are indicated in magenta and in separate channels as labelled. To clarify cell-autonomy, cell death is also shown in higher magnification in Figure 7—figure supplement 2. (**A–D**) Clones expressing RNAi for eIF4G. (**E–H**) Clones expressing RNAi for eEF2. (**I–L**) Clones expressing RNAi for eIF6. In all cases (panels A,E,I), wild-type cells near to cells depleted for translation factors show higher translation rate than other wild type cells. (**M**) Clones of cells depleted for TAF1B (green) also showed a cell-autonomous reduction in translation rate and non-autonomous increase in nearby wild-type cells (translation rate in magenta, see also M’). (**N**) Clones of cells depleted for TAF1B (green) showed a non-autonomous increase in RpS6 phosphorylation in nearby cells (magenta, see also N’). Additional data relevant to this Figure is shown in Figure 7—figure supplement 1 and Figure 7—figure supplement 2. Genotypes: A, B, D: {hs:FLP}/+; UAS – RNAi^eIF4G^ /+; act> CD2> Gal4, UAS-GFP /+ (line: v17002), C:{hs:FLP}/+; UAS – RNAi^eIF4G^ /+; act> CD2> Gal4, UAS-GFP /Xrp1-HA (line: v17002), E, F, H: {hs:FLP}/+; UAS – RNAi^eEF2^ /+; act> CD2> Gal4, UAS-GFP /+ (line: v107268), G: {hs:FLP}/+; UAS – RNAi^eEF2^ /+; act> CD2> Gal4, UAS-GFP / Xrp1-HA (line: v107268), I, J, L:{hs:FLP}/+; UAS – RNAi ^eIF6^ /+; act> CD2> Gal4, UAS-GFP / + (line: v108094), K: {hs:FLP}/+; UAS – RNAi^eIF6^ /+; act> CD2> Gal4, UAS-GFP / Xrp1-HA(line: v108094), M, N: p{hs:FLP}/+; UAS-RNAi^TAF1B^/+;act> CD2> Gal4, UAS- GFP /+ (line: Bl 61957).

All these depletions exhibited severe reduction in translation rate in the third instar larvae, as did TAF1B depletion (Figure 7A, E, I and M; Figure 7—figure supplement 1A, E; the fact that clones of cells expressing these dsRNAs could be recovered with such low translation suggests that translation factor depletion probably exacerbates over time, initially being insufficient to prevent translation and growth, but eventually becoming severe). Importantly, all these translation factor depletions resulted in more dramatic induction of apoptosis in depleted cells that were close to wild-type cells than within the clones, suggesting that differences in translation rate might be sufficient to initiate cell competition (Figure 7B, F and J; Figure 7—figure supplement 1B, F; Figure 7—figure supplement 2). Interestingly, in all these cases translation increased in wild-type cells near to the affected clones, something that was rare adjacent to *Rp*^+/-^ cells and not seen adjacent to cells depleted for PPP1R15, although it was observed near to TAF1B depleted cells (Figure 7A, E, I and M; Figure 7—figure supplement 1A, E). Phosphorylated RpS6 accumulated in wild-type cells adjacent to TAF1B depleted cells, suggesting that a non-autonomous activation of Tor accounts for the increased translation in cells nearby those with translation deficits (Figure 7N; Laplante and Sabatini, 2012; Romero-Pozuelo et al., 2017).

**Figure 7—figure supplement 1.**
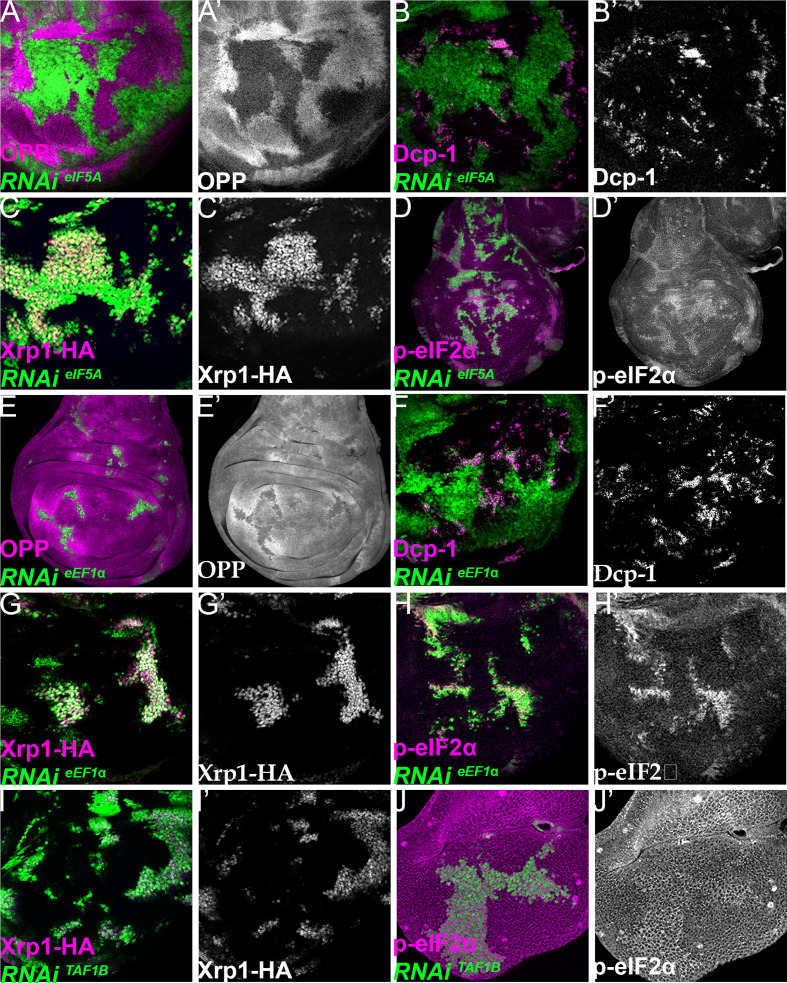
Xrp1 expression, eIF2α phosphorylation, reduced translation, and cell competition after depletion of additional translation factors. Clones of cells depleted for translation factors are shown in green. In each case, translation factor depletion reduced translation rate, resulted in competitive cell death at interfaces with wild-type cells, induced Xrp1-HA expression, and led to eIF2α phosphorylation. Translation rate, dying cells (activated caspase Dcp1), Xrp1-HA and p-eIF2α are indicated in magenta and in separate channels as labelled. To clarify cell-autonomy, cell death is also shown in higher magnification in Figure 7—figure supplement 2. (**A–D**) Clones expressing RNAi for eIF5A. (**E–H**) Clones expressing RNAi for eEF1α. (**I,J**) Clones expressing RNAi for TAF1B. (**I,J**) Clones expressing RNAi for TAF1B. Genotypes: A, B, D: {hs:FLP}/+; UAS – RNAi^eIF5A^ /+; act> CD2> Gal4, UAS-GFP / + (line: v101513), C: {hs:FLP}/+; UAS – RNAi ^eIF5A^ /+; act> CD2> Gal4, UAS-GFP / Xrp1-HA (line: v101513), E, F, H: {hs:FLP}/+; UAS – RNAi^eEF1α1^ /+; act> CD2> Gal4, UAS-GFP /+ (line: v104502), G: {hs:FLP}/+; UAS – RNAi^eEF1α1^ /+; act> CD2> Gal4, UAS-GFP / Xrp1-HA (line: v104502), I: p{hs:FLP}/+; UAS- RNAi^TAF1B^ /+;act> CD2> Gal4, UAS- GFP / Xrp1-HA (line: v105873), J: p{hs:FLP}/+; UAS- RNAi^TAF1B^ /+;act> CD2> Gal4, UAS- GFP /+ (line: Bl 61957).

**Figure 7—figure supplement 2.**
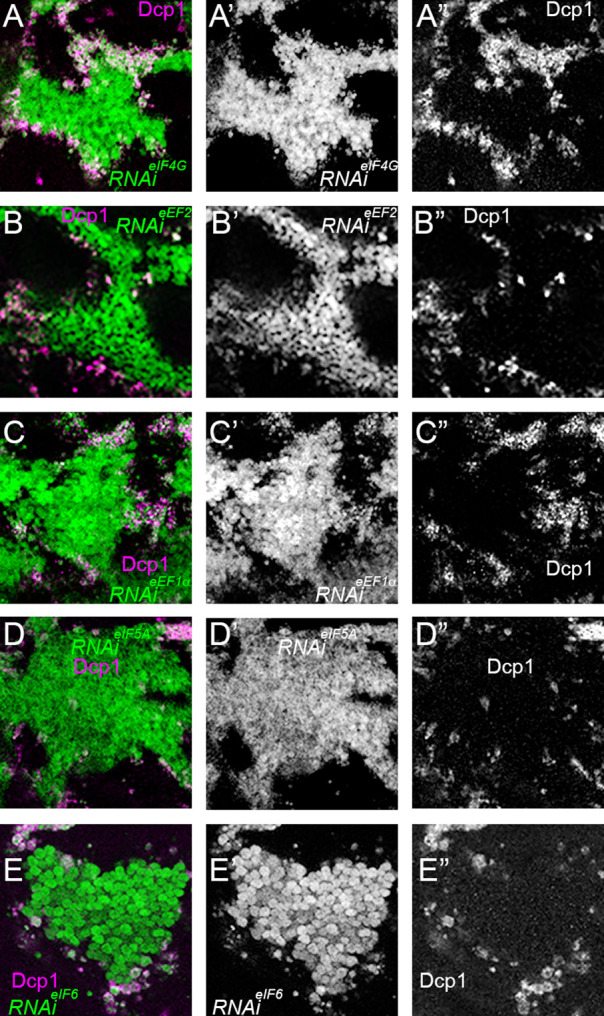
Cell-autonomy of cell death after translation factor depletion. All panels show cell death, indicated by Dcp1 labeling in magenta in panels A-E and in white in panels A”-E”, associated with clones of cells depleted for the indicated translation factors (green in panels A-E and white in panels A’-E’). The images shown are enlargements of portions of Figure 7B, F and J and Figure 7—figure supplement 1 panels B, F. In all cases, the large majority of the dying cells are cells depleted for translation factors close to the wild-type cells (black in panels A-E and A’-E’), with only a few depleted cells dying away from the clone boundary, and only a few dying wild-type cells. (**A-A”**) RNAi for eIF4G; (**B**) RNAi for eEF2; (**C**) RNAi for eEF1α(**D**) RNAi for eIF5A; (**E**) RNAi for eIF6.

**Figure 7—figure supplement 3.**
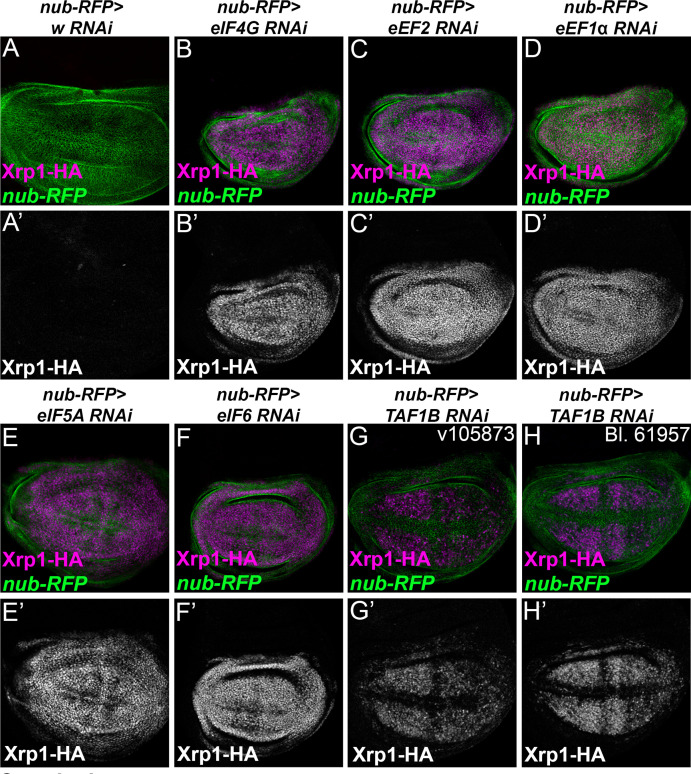
Translation factor knock-down induces Xrp1 expression. Knock down of translation factors using nub-Gal4 to drive RNAi in the wing pouch (green) induced Xrp1-HA expression (magenta, and separate channels as indicated). (**A**) In the control of nub-Gal4 expressing RFP along with w-RNAi, negligible Xrp1-HA was detected (see A’). (**B**) eIF4G-RNAi. (**C**) eEF2- RNAi. (**D**) eEF1α1-RNAi. (**E**) eIF5A-RNAi. (**F**) eIF6-RNAi. (**G**) TAF1B-RNAi (**H**) TAF1B-RNAi. Genotypes: A: nubGal4, UAS-RFP/+; Xrp1-HA/RNAi^w^, B: nubGal4, UAS-RFP/ UAS – RNAi^eIF4G^; Xrp1-HA/+, C: nubGal4, UAS-RFP/ UAS –RNAi^eEF2^; Xrp1-HA/+, D: nubGal4, UAS-RFP/ UAS – RNAi^eEF1α1^; Xrp1-HA/+, E: nubGal4, UAS-RFP/ UAS – RNAi ^eIF5A^; Xrp1-HA/+, F: nubGal4, UAS-RFP/ UAS – RNAi ^eIF6^; Xrp1-HA/+, G: nubGal4, UAS-RFP/ RNAi^TAF1B^; Xrp1-HA/+ (v105873), H: nubGal4, UAS-RFP/ RNAi^TAF1B^; Xrp1-HA/+ (line: BL 61957).

**Figure 8.**
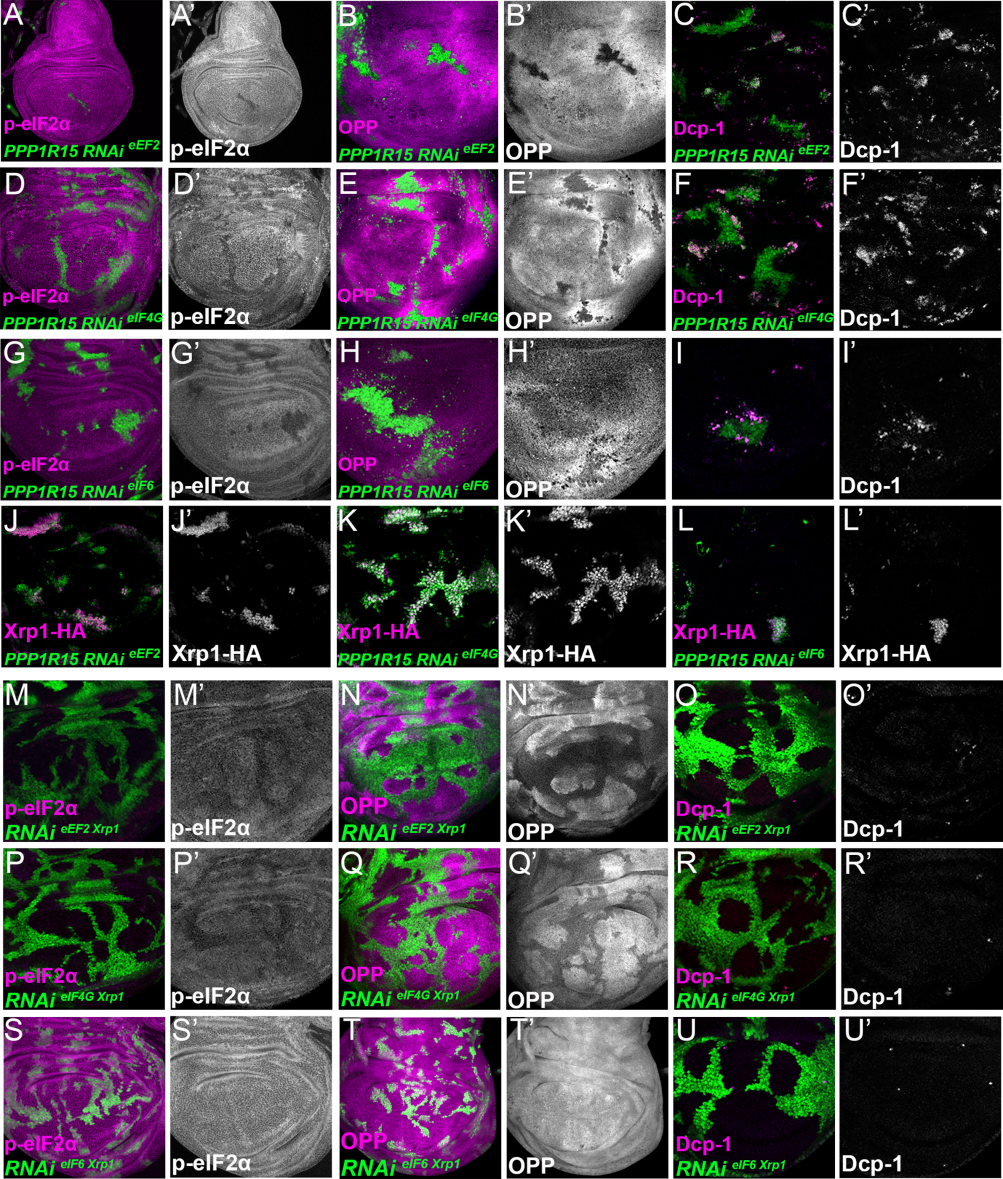
Interrupting the translation cycle activates Xrp1-dependent cell competition, independently of diminished translation. Single confocal planes from third instar wing imaginal discs. p-eIF2α levels, translation rate (ortho-propargyl puromycin), dying cells (activated caspase Dcp1) and Xrp1-HA are indicated in magenta and in separate channels as labelled. (**A–L**) Clones of cells depleted for translation factors which also overexpress PPP1R15 are shown in green. In each case, PPP1R15 overexpression was sufficient to reduce eIF2α phosphorylation to near control levels (or even lower), but it did not restore normal translation rates, did not affect Xrp1-HA levels and did not reduce competitive cell death. (**A–C**) Clones co-expressing PPP1R15 and RNAi for eEF2. (**D–F**) Clones co-expressing PPP1R15 and RNAi eIF4G. (**G-I**) Clones co-expressing PPP1R15 and RNAi for eIF6. (**J–K**) Xrp1-HA expression (magenta) in clones co-expressing PPP1R15 and RNAi for eEF2 (**J**), eIF4G (**K**), or eIF6 (**L**). (**M–U**) Clones of cells depleted for translation factors which also express Xrp1-RNAi are shown in green. (**M–O**) Clones depleted for Xrp1 as well as eEF2 expressed phospho-eIF2α at near to control levels, only partially restored overall translation rate, but lacked competitive cell death. (**P–R**) Clones depleted for Xrp1 as well as eIF4G expressed phospho-eIF2α at near to control levels, only partially restored overall translation rate, but lacked competitive cell death. (**S–U**) Clones depleted for Xrp1 as well as eIF6 expressed phospho-eIF2α at near to control levels, restored overall translation rate and lacked competitive cell death. Genotypes: A-C: {hs:FLP}/+; UAS – RNAi^eEF2^/ UAS-PPP1R15; act> CD2> Gal4, UAS-GFP / +, D-F: {hs:FLP}/+; UAS – RNAi^eIF4G^ /UAS-PPP1R15; act> CD2> Gal4, UAS-GFP / +, G-I: {hs:FLP}/+; UAS – RNAi^eIF6^/ UAS-PPP1R15; act> CD2> Gal4, UAS-GFP / +, J: {hs:FLP}/+; UAS – RNAi^eEF2^/ UAS-PPP1R15; act> CD2> Gal4, UAS-GFP / Xrp1-HA, K: {hs:FLP}/+; UAS – RNAi^eIF4G^ /UAS-PPP1R15; act> CD2> Gal4, UAS-GFP / Xrp1-HA, L: {hs:FLP}/+; UAS – RNAi^eIF6^/ UAS-PPP1R15; act> CD2> Gal4, UAS-GFP / Xrp1-HA, M-O: {hs:FLP}/+; UAS – RNAi^eEF2^/ UAS- RNAi^Xrp1^; act> CD2> Gal4, UAS-GFP /+, P-R: {hs:FLP}/+; UAS – RNAi^eIF4G^ /UAS- RNAi^Xrp1^; act> CD2> Gal4, UAS-GFP / +, S-U: {hs:FLP}/+; UAS – RNAi^eIF6^/ UAS- RNAi^Xrp1^; act> CD2> Gal4, UAS-GFP / +.

To confirm that translation factor depletion affected translation directly, and downstream of Xrp1 and PERK, Xrp1 expression and eIF2α phosphorylation were examined. Unexpectedly, depletion for translation factors was associated with both cell-autonomous induction of Xrp1 expression and eIF2α phosphorylation (Figure 7C, D, G, H, K and L; Figure 7—figure supplement 1C,D,G,H; Figure 7—figure supplement 3). The levels were at least comparable to those of TAF1B-depleted cells (Figure 7—figure supplement 1I, J). When Xrp1 was knocked-down, PPP1R15 overexpressed, or PERK depleted simultaneously with translation factor depletion, the translation factor depletions behaved similarly to one another, and also similarly to TAF1B knock-down. PPP1R15 overexpression reduces eIF2α phosphorylation even in wild type cells, without increasing their global translation rate or affecting survival (Figure 8—figure supplement 1A-F). In translation-factor-depleted cells, PPP1R15 overexpression also reduced eIF2α phosphorylation to or even below control levels (Figure 8A, D and G, Figure 8—figure supplement 1G, J and M, Figure 8—figure supplement 4), but this did not restore normal translation rates (Figure 8B, E and H, Figure 8—figure supplement 1H, K and N). There was no rescue of competitive cell death (Figure 8C, F and I; Figure 8—figure supplement 1I, L and O) or Xrp1 expression (Figure 8J–L; Figure 8—figure supplement 1P, Q and R). PERK knock-down similarly did not affect Xrp1 expression or rescue competitive cell death in translation-factor knock-downs or TAF1B knock-down (Figure 8—figure supplement 2). Knockdown of Xrp1 reduced levels of eIF2α phosphorylation in some cases (Figure 8M, P and S Figure 8—figure supplement 3J), although for eIF5A and eEF1α1 the reduction was only partial so that both the eIF5A Xrp1 depleted and eEF1α1 Xrp1-depleted cells retained more eIF2α phosphorylation than wild-type cells (Figure 8—figure supplement 3D, G). For all the translation factors, however, Xrp1 depletion eliminated or strongly reduced cell death at the competing cell boundaries, irrespective of whether eIF2α phosphorylation remained (Figure 8O, R and U; Figure 8—figure supplement 3F, I). We also found that overall translation rate, as estimated by OPP incorporation, was only partially restored by simultaneous Xrp1 depletion from most translation factor knock-down cells, and remained lower than wild type cells (Figure 8N and Q; Figure 8—figure supplement 3H). Remarkably, simultaneous knock-down of Xrp1 along with eIF6 resulted in translation rates similar to or higher than in wild type cells (Figure 8T). We have also seen this with eEF1α Xrp1 double knockdown (Figure 8—figure supplement 3E), but interpretation is difficult because some clones depleted only for eEF1α also had higher OPP labeling. Reduced translation upon TAF1B knock-down was also Xrp1-dependent (Figure 8—figure supplement 3K, L), although Xrp1 depletion had no effect on eIF2α phosphorylation, global translation, or cell survival of otherwise wild-type cells (Figure 8—figure supplement 3A-C).

**Figure 8—figure supplement 1.**
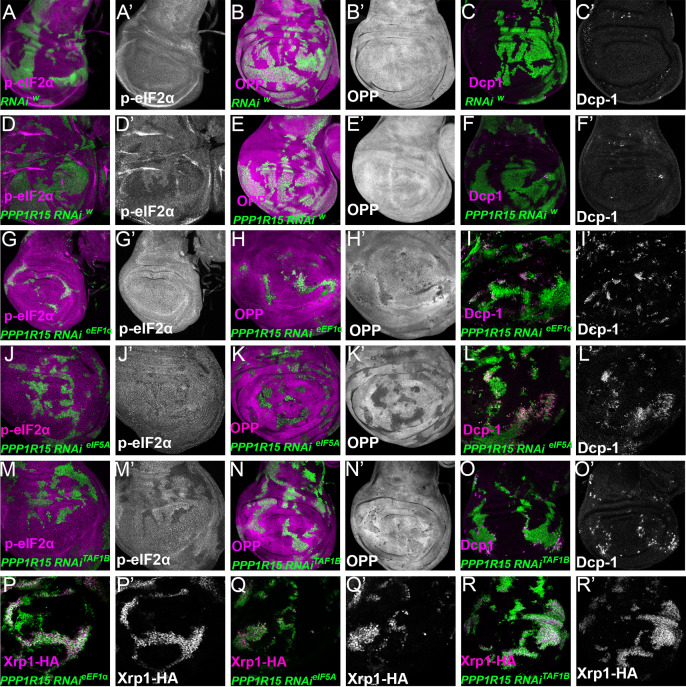
Translation and transcription factor knock down affect global translation, cell competition, and Xrp1 expression, independently of PPP1R15 over-expression. Single confocal planes from third instar wing imaginal discs. p-eIF2α levels, translation rate (ortho-propargyl puromycin), dying cells (activated caspase Dcp1) and Xrp1-HA are indicated in magenta and in separate channels as labelled. (**A–C**) Control clones overexpressing RNAi against white showed no effect in p-eIF2a levels(A), translational rates (**B**) or cell death (**C**). (**D–F**) Clones of cells depleted for white gene which also overexpress PPP1R15 (shown in green) present lower levels of eIF2α phosphorylation, but had no effect on translational levels or cell death. (**G–R**) Clones of cells depleted for translation factors which also overexpress PPP1R15 are shown in green. PPP1R15 overexpression reduced eIF2α phosphorylation in the cells depleted for eEF1α (**G**), eIF5A (**J**) or TAF1B (**M**), but did not restore translation levels back to normal (**H, K,N**). PPP1R15 overexpression did not reduce Xrp1-HA levels or rescue competitive cell death in the cells depleted for eEF1α (**I, P**), eIF5A (**L, Q**) or TAF1B (**O, R**). Genotypes: A-C: {hs:FLP}/+; act> CD2> Gal4, UAS-GFP / UAS – RNAi^w^, D-F: {hs:FLP}/+; UAS-PPP1R15/+; act> CD2> Gal4, UAS-GFP / UAS – RNAi^w^,G-I: {hs:FLP}/+; UAS – RNAi^eEF1α1^ /UAS-PPP1R15; act> CD2> Gal4, UAS-GFP /+ (line: v104502), J-L: {hs:FLP}/+; UAS – RNAi^eIF5A^ /UAS-PPP1R15; act> CD2> Gal4, UAS-GFP / + (line: v101513), M-O: p{hs:FLP}/+; UAS- RNAi^TAF1B^ /UAS-PPP1R15;act> CD2> Gal4, UAS- GFP /+ (line: v105873), P: {hs:FLP}/+; UAS – RNAi^eEF1α1^ /UAS-PPP1R15; act> CD2> Gal4, UAS-GFP / Xrp1-HA (line: v104502), Q: {hs:FLP}/+; UAS – RNAi^eIF5A^ / UAS-PPP1R15; act> CD2> Gal4, UAS-GFP / Xrp1-HA (line: v101513), R: p{hs:FLP}/+; UAS- RNAi^TAF1B^ / UAS-PPP1R15;act> CD2> Gal4, UAS- GFP / Xrp1-HA (line: v105873).

**Figure 8—figure supplement 2.**
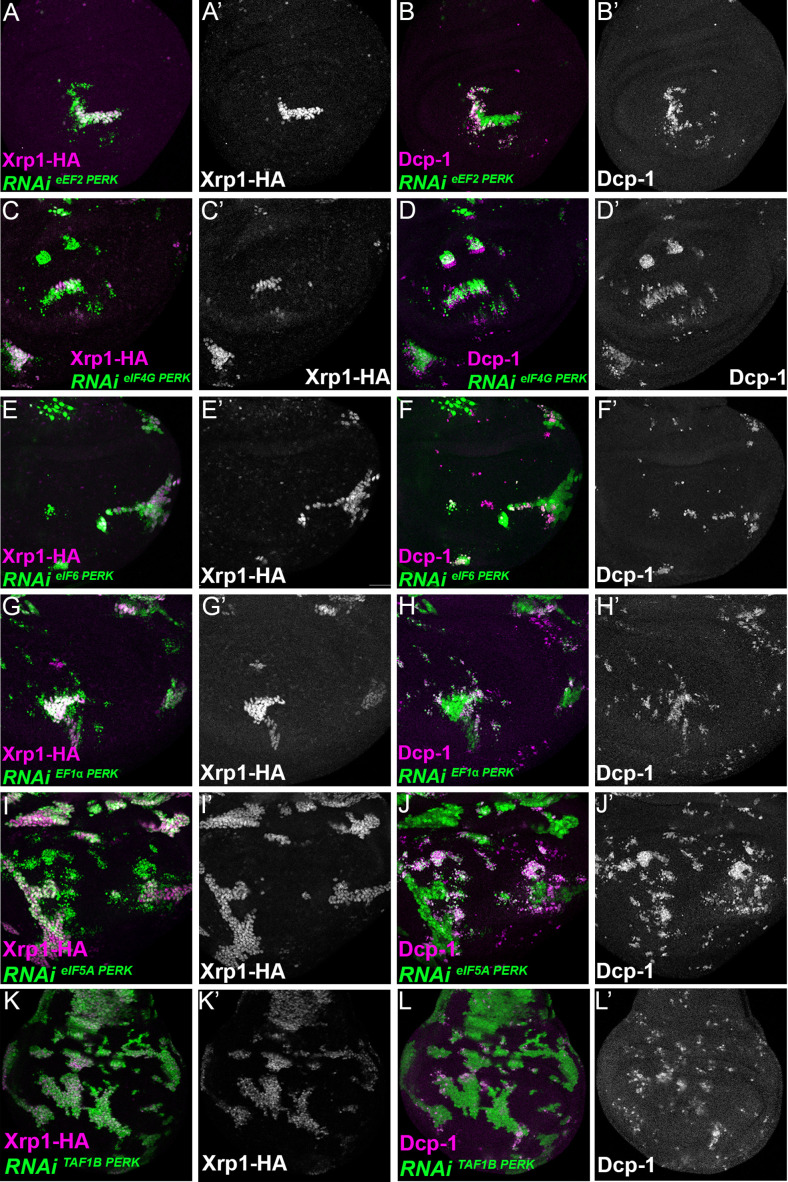
Translation and transcription factor knock down affect Xrp1 expression, and cell survival, independently of PERK knock-down. Single confocal planes from third instar wing imaginal discs. Dying cells (activated caspase Dcp1) and Xrp1-HA are indicated in magenta and in separate channels as labelled. Clones of cells depleted for translation factors which also overexpress PERK-RNAi (v110278)are shown in green. In no case did PERK depletion affect Xrp1-HA induction or suppress competitive cell death. (**A,B**) Clones expressing RNAi for both eEF2 and PERK. (**C,D**) Clones expressing RNAi for both eIF4G and PERK. (**E,F**) Clones expressing RNAi for both eIF6 and PERK. (**G,H**) Clones expressing RNAi for both eEF1α1 and PERK. (**I,J**). Clones expressing RNAi for both eIF5A and PERK. (**K,L**). Clones expressing RNAi for both TAF1B and PERK. Genotypes: A: {hs:FLP}/+; UAS – RNAi^eEF2^/ UAS-RNAi^PERK^; act> CD2> Gal4, UAS-GFP / Xrp1-HA, B: {hs:FLP}/+; UAS – RNAi^eEF2^/ UAS-RNAi^PERK^; act> CD2> Gal4, UAS-GFP /+, C: {hs:FLP}/+; UAS – RNAi^eIF4G^ /UAS-RNAi^PERK^; act> CD2> Gal4, UAS-GFP / Xrp1-HA, D: {hs:FLP}/+; UAS – RNAi^eIF4G^ /UAS-RNAi^PERK^; act> CD2> Gal4, UAS-GFP /+, E: {hs:FLP}/+; UAS – RNAi^eIF6^/UAS-RNAi^PERK^; act> CD2> Gal4, UAS-GFP / Xrp1-HA, F: {hs:FLP}/+; UAS – RNAi^eIF6^/ UAS-RNAi^PERK^; act> CD2> Gal4, UAS-GFP /+, G: {hs:FLP}/+; UAS – RNAi ^eEF1α1^/UAS-RNAi^PERK^; act> CD2> Gal4, UAS-GFP / Xrp1-HA, H: {hs:FLP}/+; UAS – RNAi^eEF1α1^ /UAS-RNAi^PERK^; act> CD2> Gal4, UAS-GFP /+, I: {hs:FLP}/+; UAS – RNAi^eIF5A^/UAS-RNAi^PERK^; act> CD2> Gal4, UAS-GFP / Xrp1-HA, J: {hs:FLP}/+; UAS – RNAi^eIF5A^ /UAS-RNAi^PERK^; act> CD2> Gal4, UAS-GFP /+, K: {hs:FLP}/+; UAS – RNAi^TAF1B^/UAS-RNAi^PERK^; act> CD2> Gal4, UAS-GFP / Xrp1-HA, L: {hs:FLP}/+; UAS – RNAi^TAF1B^ /UAS-RNAi^PERK^; act> CD2> Gal4, UAS-GFP /+.

**Figure 8—figure supplement 3.**
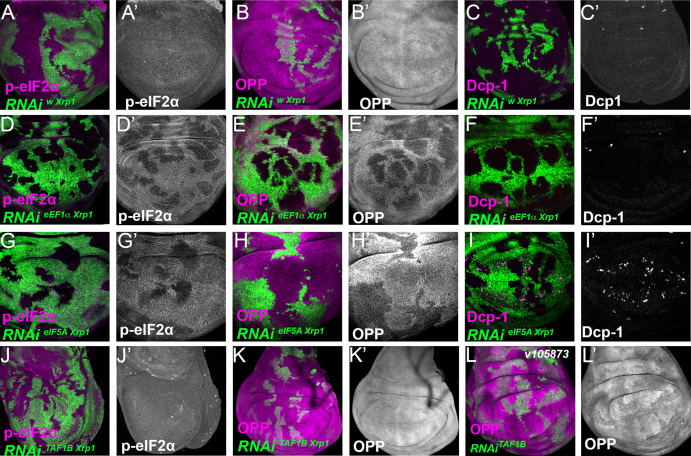
Role of Xrp1 in eIF2α phosphorylation and global translation after knockdown of translation or transcription factors. Single confocal planes from third instar wing imaginal discs. p-eIF2α levels, translation rate (ortho-propargyl puromycin), dying cells (activated caspase Dcp1) and Xrp1-HA are indicated in magenta and in separate channels as labelled. (**A–C**) Control clones overexpressing RNAis against both white and Xrp1 showed no effect in p-eIF2a levels(A), translational rates (**B**) or cell death (**C**). (**D–F**) Clones depleted for Xrp1 as well as eEF1α retained high levels of eIF2α phosphorylation but actually showed a global translation rate higher than wild type cells. They lacked competitive cell death. (**G–I**) Clones of cells depleted eIF5A that also express RNAi for Xrp1 are shown in green. Xrp1-depletion did not reduce eIF2α phosphorylation or restore translation levels, but it reduced levels of competitive cell death (compare panel I and panel B in Figure 7—figure supplement 1). (**J–L**) Clones depleted for Xrp1 as well as TAF1B expressed phospho-eIF2α at near to control levels and completely restored overall translation rate. Genotypes: A-C: {hs:FLP}/+; UAS- RNAi^Xrp1^/+; act> CD2> Gal4, UAS-GFP / UAS – RNAi^w^, D-F: {hs:FLP}/+; UAS – RNAi^eEF1α1^ / UAS-RNAi^Xrp1^; act> CD2> Gal4, UAS-GFP /+ (line: v104502), G-I: {hs:FLP}/+; UAS – RNAi^eIF5A^ / UAS- RNAi^Xrp1^; act> CD2> Gal4, UAS-GFP /+ (line: v101513), J-K: {hs:FLP}/+; UAS – RNAi^TAF1B^ / UAS- RNAi^Xrp1^; act> CD2> Gal4, UAS-GFP /+ (line: v105873), L: {hs:FLP}/+; UAS – RNAi^TAF1B^ / +; act> CD2> Gal4, UAS-GFP /+ (line: v105873).

**Figure 8—figure supplement 4.**
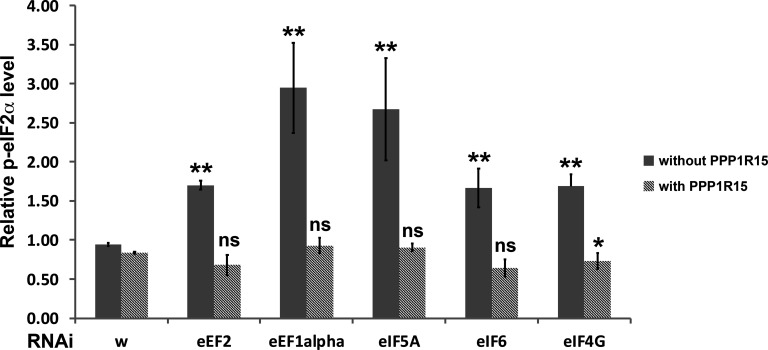
Quantification of p-eIF2α levels. Relative p-eIF2α levels within RNAi-expressing clones from Figures 6 and 7 (and supplements) were measured as average pixel intensities of single confocal planes using Fiji. Results for clones in each wing disc were pooled and normalized to the p-eIF2α level of wild type regions of the same disc. Mean and standard deviation from multiple discs are shown. Statistical significance is shown for each genotype compared to the w RNAi control or w RNAi+ PPP1 R15 over-expression control, respectively (** - p < 0.01; * - p < 0.05; ns – p ≥ 0.05). In addition, every genotype differed significantly when PPP1R15 was over-expressed. Exact p values, compared to wRNAi: eEF2, p = 3.19 × 10^–6^; eEF1α, p = 0.00263; eIF5A, p = 0.00588; eIF6, p = 0.00204; eIF4G, p = 0.00104. Exact p values, compared to wRNAi+ PPP1 R15 over-expression: eEF2+ PPP1 R15, p = 0.175; eEF1α, p = 0.068; eIF5A, p = 0.071; eIF6, p = 0.0954; eIF4G, p = 0.0227. Exact p values, comparing the same genotypes with and without PPP1R15 over-expression: w, p = 0.00231; eEF2, p = 1.95 × 10^–5^;eEF1α, p = 0.00255; eIF5A, p = 0.00584; eIF6, p = 0.00104; eIF4G, p = 1.32 × 10^–7^. Statistics: pairs of genotypes were compared by t test and the Benjamini-Hochberg procedure for multiple testing used to adjust FDR to 0.05.

These results unexpectedly show that translation factor or polI depletion triggers similar effects to depletion of ribosome components in *Rp* mutants, in which Xrp1 expression leads to eIF2α phosphorylation and to cell competition. The results separate eIF2α phosphorylation from cell competition, however, because Xrp1-dependent competitive cell death continued even when eIF2α phosphorylation levels was restored to normal by PPP1R15 overexpression, and because remaining eIF2α phosphorylation in eIF5A Xrp1-depleted and eEF1α1 Xrp1-depleted cells did not lead to cell competition. The results also separate cell competition from differences in translation levels, because no competitive cell death was observed in eIF4G Xrp1-depleted, eIF5A Xrp1-depleted, and eEF2 Xrp1-depleted cells, even though their translation was lower than the nearby wild type cells. Indeed, depletion for eIF6 or TAF1B induced Xrp1 and cell competition, even though without Xrp1 these cells seemed to translate at similar or higher rates to their neighbors. These results focus attention on Xrp1 as the key effector of cell competition, irrespective of eIF2α phosphorylation and overall translation rate.

**Figure 9.**
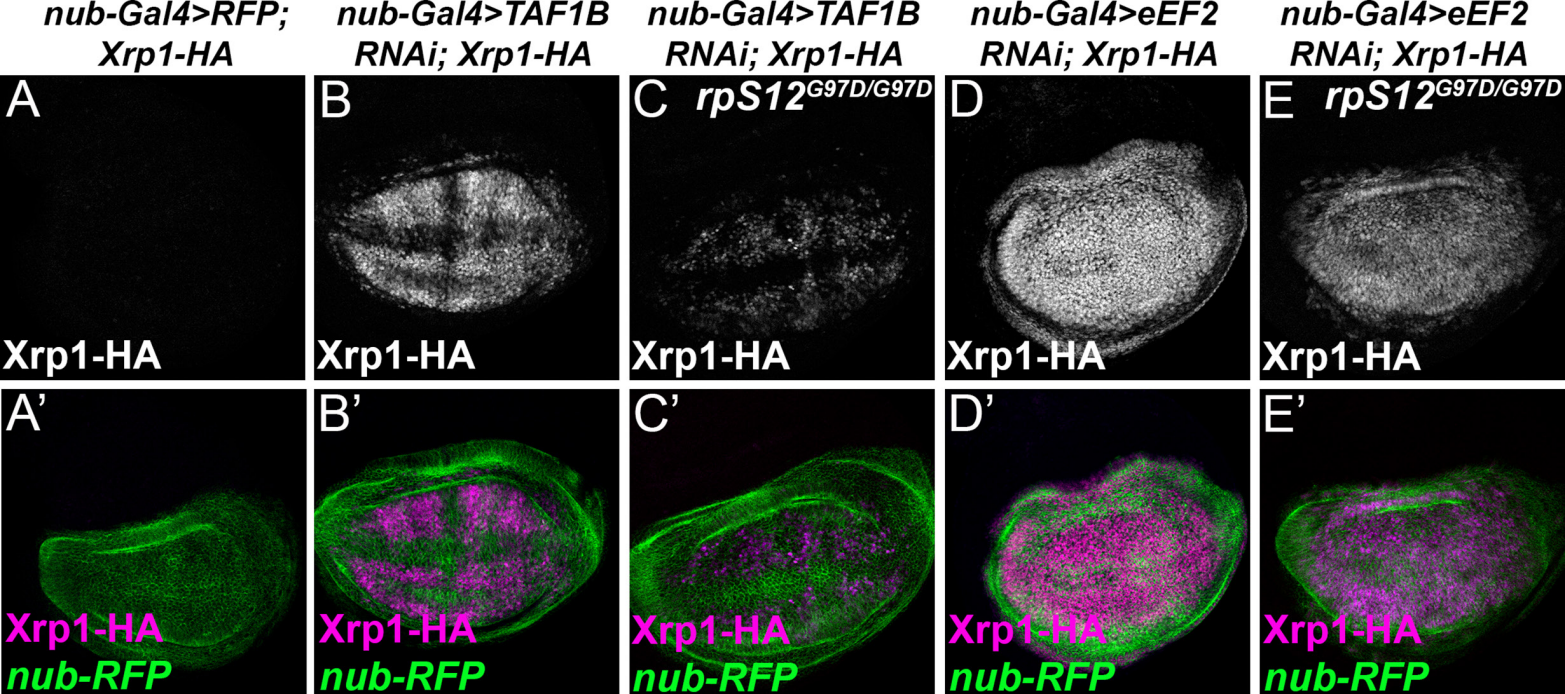
RpS12-dependence of Xrp1 expression. Figures show projections of Xrp1-HA expression from the wing discs of indicated genotypes. (**A**) Neglegible Xrp1-HA (magenta in A’) was expressed in control discs where *nub-Gal4* drove only reporter RFP expression in the wing pouch (green in A’-E’). (**B**) TAF1B knockdown resulted in Xrp1-HA expression (magenta in B’). (**C**) Xrp1-HA expression was greatly reduced when TAF-1B was knocked-down in the *rpS12^G97D^* background (see also magenta in C’). (**D**) eEF2 knockdown resulted in strong Xrp1-HA expression (magenta in D’). (**E**) Xrp1-HA expression was only moderately reduced when eEF2 was knocked-down in the *rpS12^G97D^* background (see also magenta in E’). Genotypes: A: nubGal4, UAS-RFP/+; Xrp1-HA/Xrp1-HA,B: nubGal4, UAS-RFP/ UAS – RNAi^TAF1B^;Xrp1-HA/ Xrp1-HA (line: v105873), C: nubGal4, UAS-RFP/ UAS –RNAi^TAF1B^; *Rps12^G97D^*, Xrp1-HA/ *Rps12^G97D^*, Xrp1-HA, D: nubGal4, UAS-RFP/ UAS – RNAi^eEF2^;Xrp1-HA/ Xrp1-HA, E: nubGal4, UAS-RFP/ UAS –RNAi^eEF2^; *Rps12^G97D^*, Xrp1-HA/ *Rps12^G97D^,* Xrp1-HA.

These results also raise the question of whether *Rp* haploinsufficiency, rRNA depletion, eIF2α phosphorylation, and translation factor depletion all activate Xrp1 through a common pathway. In *Rp*^+/-^ genotypes, Xrp1 expression depends on a specific ribosomal protein, RpS12, and is almost completely prevented by *rpS12^G97D^*, a mis-sense allele that specifically affects this aspect of RpS12 function (Lee et al., 2018; Ji et al., 2019). We found that *rpS12^G97D^* homozygosity also reduced Xrp1 induction when TAF1B was depleted (Figure 9A–C), but had much less effect when eEF2 was depleted (Figure 9D–E). Thus, the mechanism of Xrp1 activation may resemble that in *Rp*^+/-^ cells when rRNA synthesis is affected, but appears distinct when translation factors are inhibited.

### Xrp1 is a transcription factor that regulates cell competition

Xrp1 is a key mediator of multiple defects in ribosome biogenesis or function. Xrp1 is a sequence-specific DNA-binding protein implicated in genome maintenance, and binds directly to sequences of the P element whose transposition it promotes (Akdemir et al., 2007; Francis et al., 2016). Xrp1 also controls expression of many genes at the mRNA level (Lee et al., 2018), and other similar bZip proteins are transcription factors (Tsukada et al., 2011).

**Figure 10.**
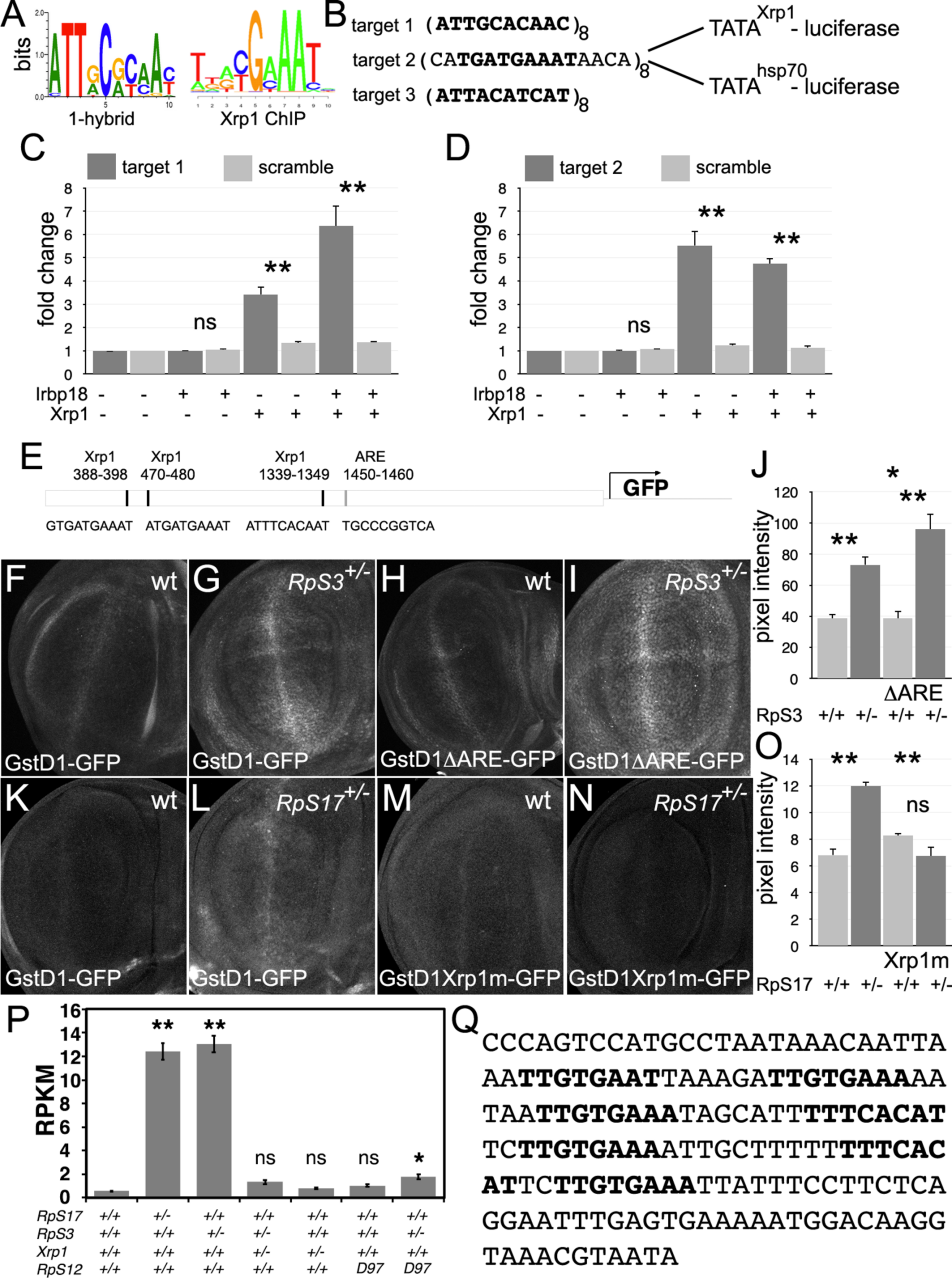
Transcriptional regulation by Xrp1. (**A**) Xrp1/Irbp18 binding consensus defined by bacterial 1-hybrid studies (Zhu et al., 2011) and by Xrp1 ChIP from *Drosophila* eye imaginal discs overexpressing an Xrp1-HA protein (Baillon et al., 2018). (**B**) Xrp1 binding motif sequences multimerized in luciferase reporter plasmids upstream of transcription start sites from the *Xrp1* gene or from the *hsp70* gene. Targets 1 and 3 were based on the 1-hybrid consensus, target 2 is the P element sequence footprinted by Xrp1-Irbp18 (Francis et al., 2016). The match to the consensus sites is shown in bold type. (**C**) Luciferase assays following transfection of reporters and protein expression plasmids into S2 cells. The target 1-TATA^Xrp1^ reporter showed sequence-specific activation by transfected Xrp1. Transfected Irbp18 alone had no effect, but synergized with Xrp1. p-Values for comparisons between target one reporters and scrambled reporters were: Padj = 1, Padj = 0.00827, Padj = 3.47 × 10^–7^, respectively. (**D**) Luciferase assays following transfection of reporters and protein expression plasmids into S2 cells. The target 2-TATA^Xrp1^ reporter showed sequence-specific activation by transfected Xrp1. Transfected Irbp18 alone had no effect. p-Values for comparisons between target two reporters and scrambled reporters were: Padj = 1, Padj = 2.00 × 10^–8^, Padj = 1.96 × 10^–7^ respectively. (**E**) Potential regulatory sequences in the 2.7 kb upstream intergenic fragment used in the GstD1-GFP reporter (Sykiotis and Bohmann, 2008; Brown et al., 2021).3 Xrp1-binding motifs and the antioxidant response element (ARE) are indicated. (**F–I**) and (**K-N**) show projections from the central disc-proper regions of wing discs expressing reporter transgenes in the indicated genetic backgrounds. (**F**) Baseline GstD1-GFP expression in the wild-type wing disc. (**G**) Elevated GstD1-GFP expression in the *RpS3^+/-^* wing disc. (**H**) Baseline GstD1ΔARE-GFP expression in the wild-type wing disc. (**I**) Elevated GstD1ΔARE-GFP expression in the *RpS3^+/-^* wing disc. (**J**) Quantification of these results. Average pixel intensity from wing pouch regions was measured. Mean± SEM from multiple samples is shown. N = 5 for each genotype. Exact p values were: for GstD1-GFP in *RpS3^+/-^* compared to *RpS3^+/+^*, Padj = 0.00257; for GstD1ΔARE-GFP in *RpS3^+/-^* compared to *RpS3^+/+^*, Padj = 2.55 × 10^–5^; for GstD1-GFP in *RpS3^+/+^* compared to GstD1ΔARE-GFP in *RpS3^+/+^*, Padj = 0.993; for GstD1-GFP in *RpS3^+/-^* compared to GstD1ΔARE-GFP in *RpS3^+/-^*, Padj = 0.0313. (**K**) baseline GstD1-GFP expression in the wild type wing disc. (**L**) Elevated GstD1-GFP expression in the *RpS17^+/-^* wing disc. (M) baseline expression of GstD1-GFP with all 3 Xrp1-binding motifs mutated in the wild type wing disc. (**N**) Expression of GstD1-GFP with all 3 Xrp1-binding motifs mutated was similar in the *RpS17^+/-^* wing disc to the wild type control. (**O**) Quantification of these results. Average pixel intensity from wing pouch regions was measured. Mean± SEM from multiple samples is shown. N = 5,6,5,6 for respective samples. Exact p values were: for GstD1-GFP in *RpS3^+/-^* compared to *RpS3^+/+^*, Padj = 2.34 × 10^–6^; for GstD1mXrp1-GFP in *RpS3^+/-^* compared to *RpS3^+/+^*, Padj = 0.116; for GstD1-GFP in *RpS3^+/+^* compared to GstD1mXrp1-GFP in *RpS3^+/+^*, Padj = 0.112; for GstD1-GFP in *RpS3^+/-^* compared to GstD1mXrp1-GFP in *RpS3^+/-^*, Padj = 1.19 × 10^–6^. (**P**) Pooled copia transcript levels for indicated genotypes determined from mRNA-seq data. Mean± standard deviation is shown. Values for individual copia insertions are shown in Figure 10—figure supplement 2. Asterisks indicate statistical significance of the difference from the wild type control: **, p < 0.01; *, p < 0.05; ns, p ≥ 0.05. Exact p values were: for RpS17^+/-^ compared to wild type, p = 4 × 10^–14^; for RpS3^+/-^ compared to wild type, p = 2.33 × 10^–14^; for RpS3^+/-^ Xrp1^+/-^ compared to wild type, p = 0.262; for RpS3^+/-^ Xrp1^+/-^ compared to wild type, p = 0.262;for Xrp1^+/-^ compared to wild type, p = 0.494; for RpS3^+/-^ Xrp1^+/-^ compared to wild type, p = 0.262; for rpS12^D97/D97^ compared to wild type, p = 0.858; for RpS3^+/-^ rpS12^D97/D97^ compared to wild type, p = 0.0201; for RpS3^+/-^ rpS12^D97/D97^ compared to wild type, p = 0.0201; for RpS3^+/-^ Xrp1^+/-^ compared to RpS3^+/-^, p = 4.91 × 10^–14^; for RpS3^+/-^ Xrp1^+/-^ compared to Xrp1^+/-^, p = 0.635; for RpS3^+/-^ rpS12^D97/D97^ compared to rpS12^D97/D97^, p = 0.251. Statistics:1-way ANOVA with Bonferroni-Holm correction for multiple testing was performed for the data shown in panels C,D,J,O,P. Data in panels C,D were based on triplicate measurements from each of three biological replicates for each transfection. Data in panel P were based on three biological replicates for each genotype. Genotypes: F: GstD1-GFP/+, G: GstD1-GFP/+; FRT82 *RpS3* p{arm:LacZ}/+, H: GstD1ΔARE-GFP/+, I: GstD1ΔARE-GFP/+; FRT82 *RpS3* p{arm:LacZ}/+, K: GstD1-GFP/+, L: GstD1-GFP; *RpS17* p{arm:LacZ} FRT80B/+, M: GstD1 Xrp1m –GFP, N: GstD1Xrp1m-GFP; *RpS17* p{arm:LacZ} FRT80B/+. Genotypes of P graph per column: 1^st^: w^11-18^; FRT82B/+, 2^nd^: w^11-18^; w p{hs:FLP}; *RpS17* p{ubi:GFP} FRT80B/+,3^rd^: w^11-18^;w p{hs:FLP}; FRT82 *RpS3* p{arm:LacZ/+, 4^th^: w^11-18^;w p{hs:FLP}; FRT82 *RpS3* p{arm:LacZ/FRT82B FRT82B *Xrp1^M2^*^–73^, 5^th^: w^11-18^; FRT82B *Xrp1^M2^*^–73^ / +, 6^th^: w^11-18^; *rpS12^D97^* FRT80B / *rpS12^D97^* FRT80B, 7^th^: w^11-18^; *rpS12^D97^* FRT80B / *rpS12^D97^* FRT80B *RpS3*. Figure 10—source data 1.Luciferase data relevant to panels C,D. Figure 10—source data 2.GFP data relevant to panel J. Figure 10—source data 3.GFP data relevant to panel O. Figure 10—source data 4.mRNA-Seq data relevant to panel P, and also to Figure 10—figure supplement 3.

To test whether Xrp1 is a transcription factor, we used a dual-luciferase reporter system in transfected S2 cells (Figure 10A–D; Figure 10—figure supplement 1). Luciferase reporter plasmids were either based on the widely-used core promoter of the *Drosophila* hsp70 gene, or on a 400 bp genomic sequence spanning the transcription start site of the *Xrp1* gene itself (Figure 10—figure supplement 2). We cloned 8 x repeats of either of two different matches to the 10 bp Xrp1/Irbp18 consensus binding site in vitro (Zhu et al., 2011), which is similar to that recently deduced from ChIP-Seq following Xrp1 overexpression in vivo (Baillon et al., 2018) (Target 1 and Target 3) or of the sequence footprinted by Xrp1/Irbp18 on the P element terminal repeat (Francis et al., 2016) (target 2), which also contains a consensus match (Figure 10A and B). When Xrp1 expression was induced in transfected S2 cells, each of these Xrp1-binding sequences conferred 3x-8x activation of luciferase expression, whereas scrambled sequences were inactive (Figure 10C, D, Figure 10—figure supplement 1A, B). In the case of target 1, several-fold further induction was achieved by co-transfection and induction of Irbp18 expression, culminating in 23 x stimulation of luciferase expression by repeats of the Target 1 sequence in conjunction with the hsp70 basal promoter (Figure 10—figure supplement 1A). Irbp18 alone was inactive in the absence of transfected Xrp1 (Figure 10C and D; Figure 10—figure supplement 1A, B). Thus, the Xrp1/Irbp18 heterodimer stimulated transcription through its cognate binding sequences.

**Figure 10—figure supplement 1.**
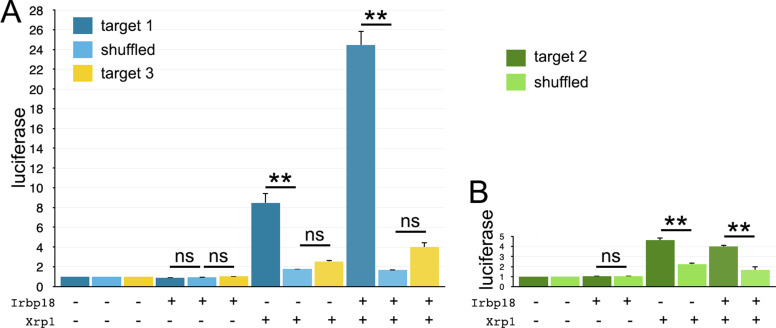
Luciferase assays with *hsp70*-based reporter plasmids. Fold change in the luciferase/renilla signal is shown for the remaining reporter constructs shown in Figure 10B. (**A**) Target one conferred sequence-specific activation by transfected Xrp1 protein. Transfected Irbp18 alone had no effect, but synergized with Xrp1. Lesser activation by Target three was rendered not significant by correction for multiple testing, but the increase in both Xrp1 and Xrp1+ Irbp18 samples suggests that it may nevertheless be real. (**B**) Target two conferred sequence-specific activation by transfected Xrp1 protein. Transfected Irbp18 alone had no effect. It may be significant that Targets 1 and 3 are better matches to the in vivo-derived ChIP-Seq consensus than Target 2 (see Figure 10A). Statistics:1-way ANOVA with Bonferroni-Holm correction for multiple testing was performed for the data shown in each of panels A,B. ns, p > 0.05.**, p < 0.01. Data were based on triplicate measurements from each of three biological replicates for each transfection. Exact p-values for comparisons between target one reporters and scrambled reporters (panel A) were: Padj = 1, Padj = 1.80 × 10^–6^, Padj = 0 for Irbp18, Xrp1, and Irpb18+ Xrp1 transfected cells respectively. Exact p-values for comparisons between target two reporters and scrambled reporters (panel A) were: Padj = 1, Padj = 1, Padj = 0.225 for Irbp18, Xrp1, and Irpb18+ Xrp1 transfected cells respectively. Exact p-values for comparisons between target three reporters and scrambled reporters (panel B) were: Padj = 0.983, Padj = 6.70 × 10^–6^, Padj = 8.03 × 10^–6^,for Irbp18, Xrp1, and Irpb18+ Xrp1 transfected cells, respectively. Figure 10—figure supplement 1—source data 1.Luciferase measurements relevant to Figure 10—figure supplement 1.

**Figure 10—figure supplement 2.**
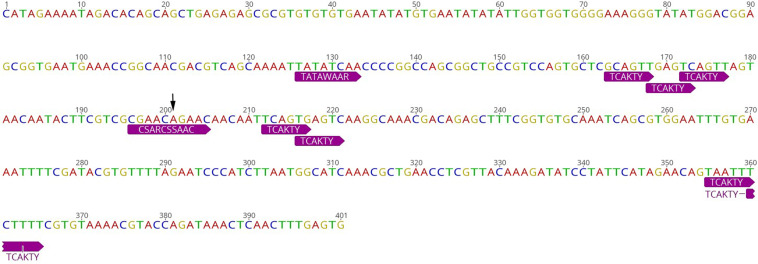
*Xrp1* promoter proximal sequences. The 400 bp *Xrp1* core promoter sequence is shown. Transcription start site indicated by arrow. A variety of conserved promoter element sequences are indicated (Ohler et al., 2002; Juven-Gershon and Kadonaga, 2010).

**Figure 10—figure supplement 3.**
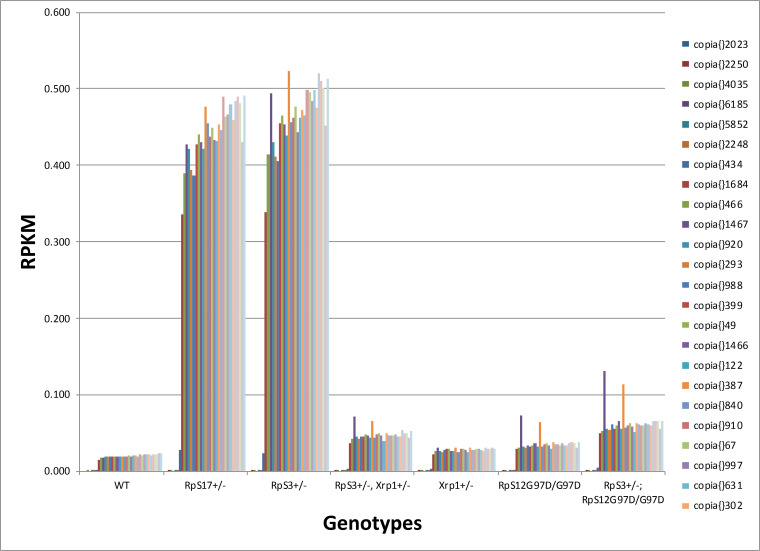
Transcription of individual Copia elements. mRNA abundances for 24 distinct copia insertions reveal generally similar regulation by RpS12 and Xrp1 in RpS17^+/-^ and RpS3^+/-^ genotypes. Genotypes of graph per column: 1^st^: w^11-18^; FRT82B/+, 2^nd^: w^11-18^; w p{hs:FLP}; *RpS17* p{ubi:GFP} FRT80B/+,3^rd^: w^11-18^;w p{hs:FLP}; FRT82 *RpS3* p{arm:LacZ/+, 4^th^: w^11-18^;w p{hs:FLP}; FRT82 *RpS3* p{arm:LacZ/FRT82B FRT82B *Xrp1^M2^*^–73^, 5^th^: w^11-18^; FRT82B *Xrp1^M2^*^–73^ / +, 6^th^: w^11-18^; *rpS12^D97^* FRT80B / *rpS12^D97^* FRT80B, 7^th^: w^11-18^; *rpS12^D97^* FRT80B / *rpS12^D97^* FRT80B *Rp.*

It has been suggested that an oxidative stress response in *Rp*^+/-^ cells leads to competition with wild type cells (Kucinski et al., 2017; Baumgartner et al., 2021). *Rp*^+/-^ cells express GstD1 reporters, whose transcription is activated by Nrf2, the master regulator of oxidative stress responses (Kucinski et al., 2017). Because the genes expressed in *Rp*^+/-^ cells are also enriched for Xrp1 binding motifs, some of these genes might be activated directly by Xrp1, including GstD1 (Ji et al., 2019 Figure 6—figure supplement 2). The GstD1-GFP reporter used to report oxidative stress in *Rp*^+/-^ cells contains a 2.7 kb genomic fragment that contains an Antioxidant Response Element (ARE) bound by the Nrf2/MafS dimer at position 1450–1460 (Figure 10E). Deletion of this motif abolishes GstD1-GFP induction in response to oxidative stress (Sykiotis and Bohmann, 2008). Recently, Brown et al identified Xrp1 binding motifs within the same GstD1-GFP reporter, and showed that these sequences are required for Xrp1-dependent induction in response to ER stress (Brown et al., 2021). We therefore compared induction of GstD1-GFP reporters in *Rp*^+/-^ wing discs where the reporter sequences were either wild type, deleted for the Nrf2 binding motif, or mutated at the Xrp1-binding motifs (Figure 10E). We found that the Nrf2 binding motif was dispensable for GstD1-GFP induction in *Rp*^+/-^ wing discs, whereas the Xrp1 sites were required, consistent with induction of GstD1-GFP and perhaps other genes as direct transcriptional targets of Xrp1, not Nrf2 (Figure 10F–O).

In addition to single copy genes, repetitive elements can also be regulated by Xrp1, as is revealed by re-analysis of previously published mRNA-seq data (Lee et al., 2018; Ji et al., 2019, Supplementary file 1). Transcription of the retrotransposon copia, for example, was elevated in RpS17 and RpS3 in an Xrp1-dependent (and RpS12-dependent) manner (Figure 10P, Figure 10—figure supplement 3). Accordingly, the regulatory, untranslated leader region of copia contains 7 copies of a motif closely matching the Xrp1 binding consensus, including 2 in a 28 bp region of dyad symmetry that is deleted from variants with reduced expression (Figure 10Q; Mount and Rubin, 1985; Sneddon and Flavell, 1989; Matyunina et al., 1996; McDonald et al., 1997; Wilson et al., 1998).

## Discussion

**Figure 11.**
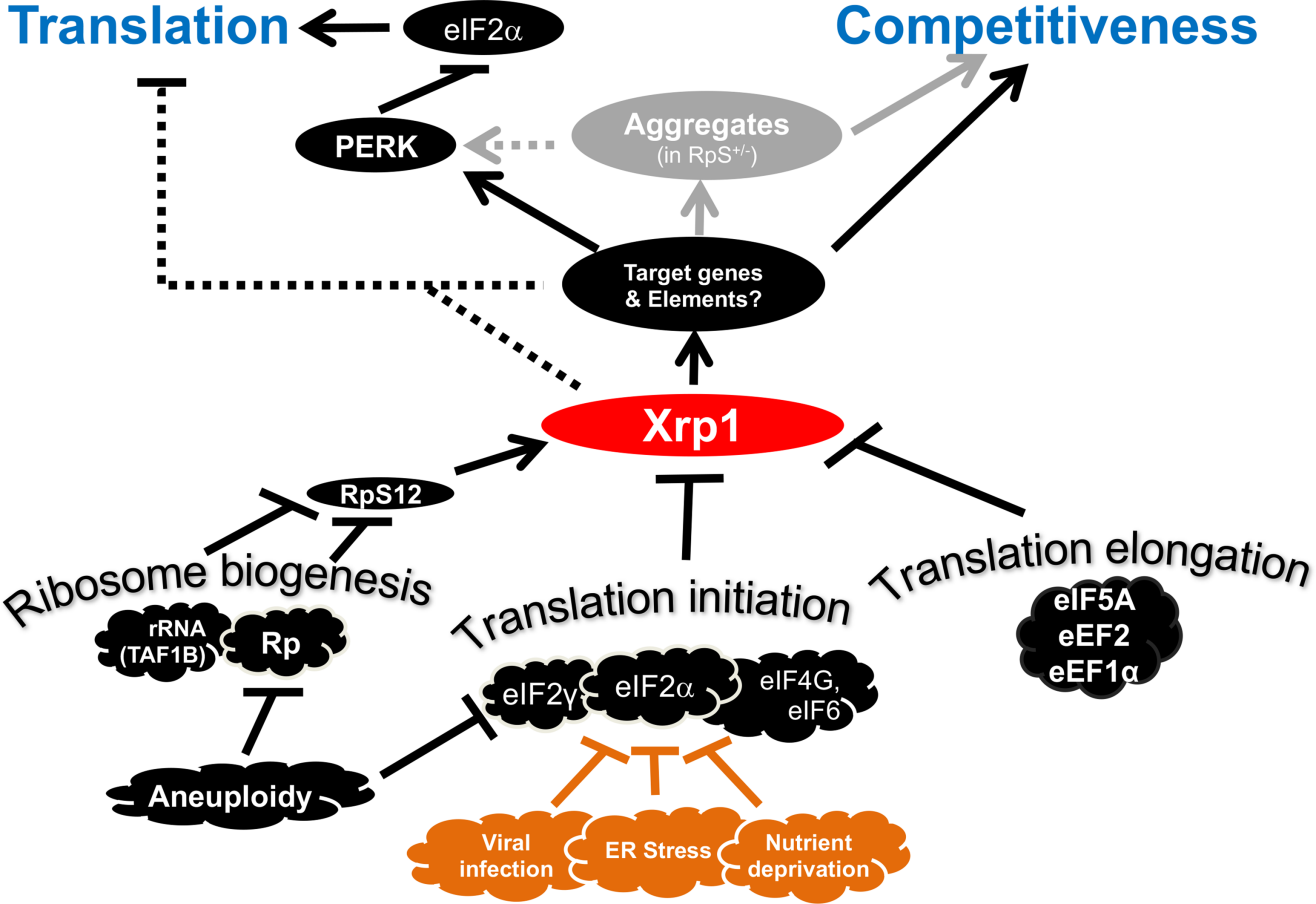
Transcriptional responses to Ribosome defects. Multiple consequences of defects in ribosome biogenesis, translation initiation, and translation elongation, depend on the transcription factor Xrp1 in the epithelial imaginal disc cells. Xrp1 is responsible for, or contributes to, reduced translation in response to these defects, through the PERK-dependent phosphorylation of eIF2α, a global regulator of CAP-dependent translation initiation. There is also evidence for some PERK-independent regulation of translation in genotypes such as TAF1B knock-down. Translation inhibition independently of Xrp1, which occurs after depletion of some translation factors, is not shown for simplicity. Xrp1 protein expression marks imaginal disc cells for elimination in competition with wild type cells. Differences in translation rate, including those caused by eIF2α phosphorylation or eIF2γ haploinsufficiency, are not sufficient to trigger cell competition without Xrp1. We speculate that other cellular stresses that phosphorylate eIF2α, including ER stress, nutrient deprivation, or (in mammals) infection with certain viruses might mark cells for competition, or interfere with cell competition that recognizes aneuploid cells on the basis of *Rp* or eIF2γ gene haploinsufficiency. It is notable that defective Tor signaling, which also reduces global translation rate, does not cause cell competition, (Baumgartner et al., 2021). Several pathways have been shown to induce Xrp1, including RpS12-dependent induction in *Rp^+/- ^*cells and TAF1B-depleted cells (Akdemir et al., 2007; Chapin et al., 2014; Lee et al., 2018; Ji et al., 2019).

We explored the mechanisms by which *Rp* mutations affect *Drosophila* imaginal disc cells, causing reduced translation and elimination by competition with wild-type cells in mosaics. Our findings reinforced the key role played by the AT-hook bZip protein Xrp1, which we showed is a sequence-specific transcription factor responsible for multiple aspects of not only the *Rp* phenotype, but also other ribosomal stresses (Figure 11). It was Xrp1, rather than the reduced levels of ribosomal subunits, that affected overall translation rate, primarily through PERK-dependent phosphorylation of eIF2α. Phosphorylation of eIF2α, as well as other disruptions to ribosome biogenesis and function such as reduction in rRNA synthesis or depletion of translation factors, were all sufficient to cause cell competition with nearby wild type cells, but this occurred because all these perturbations activated Xrp1, not because differences in translation levels between cells were sufficient to cause cell competition directly. In fact, our data show that differences in translation are neither sufficient nor necessary to trigger cell competition, which therefore depends on other Xrp1-dependent processes. Protein aggregation and activation of ‘oxidative stress response’ genes were also downstream effects of Xrp1 activity. While this paper was in preparation, other groups have also reported relationships between eIF2α phosphorylation, cell competition, and Xrp1 (Baumgartner et al., 2021; Brown et al., 2021; Langton et al., 2021; Ochi et al., 2021; Recasens-Alvarez et al., 2021),but none have reached the same overall conclusions as this study.

Our findings lead to a picture of Xrp1 as the key instigator of cell competition in response to multiple genetic triggers. Failure to appreciate the role of Xrp1 may have led to questionable conclusions in some previous studies. Our findings confirm the central importance of the transcriptional response to *Rp* mutations, and to other disruptions of ribosome biogenesis and function. They suggest therapeutic approaches to ribosomopathies, and have implications for the surveillance of aneuploid cells.

### Xrp1 activation by *Rp* gene haploinsufficiency

Rp gene haploinsufficiency has been proposed to affect ribosome concentrations, and hence translation, lead to the accumulation of ribosome components and assembly intermediates, and cause proteotoxic stress. Any of these could have been responsible for activating Xrp1 in *Rp^+/-^* cells.

Our data show that in fact ribosome subunit concentration is only moderately affected by *Rp* haploinsufficiency. We have seen 15–20% reduction in LSU concentrations in several *RpL* mutants, and 20–25% reduction in SSU concentrations in several *RpS* mutants. *RpL14^+/-^* also reduced SSU ~ 25%. Ribosomal subunit levels were unaffected by *Xrp1*. Broadly similar results have been reported in yeast (Cheng et al., 2019), and by mass spec quantification of ribosomal proteins in *RpS3^+/-^* and *RpS23^+/-^ Drosophila* wing discs (Baumgartner et al., 2021; Recasens-Alvarez et al., 2021).

Multiple explanations for the modest effects on ribosome subunit number are possible. We particularly point out that, even if expression of a particular Rp is reduced in proportion to a 50% reduction in mRNA level, the respective protein concentration (i.e. number of molecules/cell volume) is unlikely to fall to 50%, because ribosomes are required for cellular growth, so that an *Rp* mutation affects the denominator in the concentration equation, as well as the numerator. It is even possible that a 50% reduction in its rate of Rp synthesis could leave steady state ribosome subunit concentration unaffected, if cellular growth rate was slowed by the same amount.

Modest changes in SSU and LSU levels could still affect ribosome function, which may depend more on the concentrations of free subunits than on total subunits. The data suggests, however, that cellular and animal models of DBA that have generally sought to achieve a 50% reduction in Rp protein expression (Heijnen et al., 2014; Khajuria et al., 2018) could be significantly more severe than occurs in DBA patients, and that actual ribosome subunit concentrations should be measured in DBA patient cells to guide future models.

We confirmed that ribosome assembly intermediates accumulate in *Drosophila* wing discs following *Rp* haploinsufficiency. In yeast, aggregates of unused Rp rapidly trigger transcriptional changes (Albert et al., 2019; Tye et al., 2019). It has been suggested proteotoxic stress might lead to eIF2α phosphorylation in *Drosophila* (Baumgartner et al., 2021; Recasens-Alvarez et al., 2021), with Xrp1 amplifying this effect (Langton et al., 2021), but we found that while Perk was responsible for eIf2α phosphorylation, it was not required for Xrp1 expression in *Rp* mutants, placing Perk and eIF2α phosphorylation downstream. Consistent with this, we show that the protein aggregates reported in *Rp^+/-^* cells (Baumgartner et al., 2021; Recasens-Alvarez et al., 2021) were only seen in some Rp mutants, all affecting the SSU, and were also a downstream consequence of Xrp1 activity, as also now seen by others (Langton et al., 2021). It remains plausible that unused ribosomal components are the initial trigger for cellular responses in *Drosophila* as in yeast, but in *Drosophila* the species involved have not yet been identified. Because Xrp1 expression depends particularly on RpS12, an RpS12-containing signaling species is one possibility (Kale et al., 2018; Lee et al., 2018; Boulan et al., 2019; Ji et al., 2019).

### *Rp* mutants affect global translation rate through eIF2α

PERK-dependent phosphorylation of eIF2α was the mechanism by which Xrp1 suppresses global translation in *Rp^+/-^* mutants.

It is interesting that Xrp1 protein levels increase under conditions of reduced global translation. Perhaps Xrp1 is one of the few genes whose translation is enhanced when eIF2α is phosphorylated (Wek, 2018; Brown et al., 2021). Although PERK is known to be activated by ER stress, the IRE/Xbp1 branch of the UPR was not unequivocally detected in *Rp^+/-^* mutants. We suspect that the UPR might be suppressed in *Rp^+/-^* mutants by Xrp1-dependent changes in transcription of Perk, BiP, and other UPR genes (Figure 11). Perhaps in proliferative tissues it is preferable to replace stressed cells than to repair them.

It will be interesting to determine whether eIF2α phosphorylation occurs in human ribosomopathies. Notably, knock-out of CReP, one of the two mouse PPP1R15 homologs, causes anemia, similar to DBA (Harding et al., 2009; Da Costa et al., 2018), and PERK-dependent eIF2α phosphorylation occurs in RpL22-deficient mouse αβ T-cells and activates p53 there (Solanki et al., 2016). Thus, inhibitors of eIF2α phosphorylation could be explored as potential DBA drugs. TAF1B depletion, which also acted through Xrp1 and eIF2α phosphorylation in *Drosophila*, is a model of Treacher Collins Syndrome (Trainor et al., 2008), and failure to release eIF6, leading to defective LSU maturation and 80 S ribosome formation, causes Schwachman Diamond syndrome (Warren, 2018), two other ribosomopathies where potential contributions of eIF2α phosphorylation are possible.

### Xrp1, not differential translation, causes competition between cells

Because eIF2α phosphorylation alone was sufficient to target cells for competitive elimination, at first it seemed that eIF2α phosphorylation was the mechanism by which Xrp1 caused cell competition, which often correlates with differences in cellular translation levels (Nagata et al., 2019). One group has suggested this (Ochi et al., 2021). Another group concluded that eIF2α phosphorylation in *Rp^+/-^* cells did not lead to cell competition (Baumgartner et al., 2021), but the opposite conclusion is corroborated by the independent finding that haploinsufficiency for the γ subunit of eIF2 also causes cell competition (Ji et al., 2021). Our conclusion is that eIF2α phosphorylation can cause cell competition but not directly. Instead, phosphorylation of eIF2α is itself sufficient to activate Xrp1 expression, as found by us and by several other groups (Brown et al., 2021; Langton et al., 2021; Ochi et al., 2021). Crucially, *Perk* inactivation restored eIF2α phosphorylation and global translation to normal in *Rp^+/-^* cells (Figure 3H, I, Figure 3—figure supplement 1G-L), without preventing cell competition, which must therefore depend on other Xrp1 targets (Figure 5—figure supplement 3). Elimination of *eIF2γ* haploinsufficient cells is also Xrp1-dependent, as expected if Xrp1 is downstream of eIF2 activity in cell competition (Ji et al., 2021).

Knock-down of factors directly involved in the translation mechanism further distinguished cell competition from differential translation levels. Different factors affected translation in diverse ways. In *Rp^+/-^* mutants, PERK-dependent phosphorylation of eIF2α suppressed global translation, which was normalized by Perk or Xrp1 depletion. PERK-dependent phosphorylation of eIF2α also contributed to the translation deficits of cells depleted for TAF1B, eIF6, and possibly eEF1α1, which were all partially restored by eIF2α dephosphorylation and fully by Xrp1 depletion, suggesting that Xrp1 can also affect translation by additional mechanisms. By contrast, translation deficits caused by eIF4G, eIF5A, or eEF2 depletion were restored little by eIF2α dephosphorylation or Xrp1 depletion, indicating Xrp1-independent effects of these factors on translation.

Several conclusions follow from studies of these factors. As noted above, reduced translation cannot be required for cell competition, because *perk^-/-^ Rp^+/-^* mutant cells are eliminated by *perk^+/-^ Rp^+/+^* cells (Figure 5—figure supplement 3). Secondly, lower translation is not sufficient for competitive elimination, because no competitive cell death was observed in eIF4G Xrp1-depleted, eIF5A Xrp1-depleted, and eEF2 Xrp1-depleted cells, even though their translation was lower than the nearby wild type cells. Another group also concluded that lower translation alone was not sufficient for cell competition, based on different data (Baumgartner et al., 2021).

Our findings focus attention on Xrp1 activity as the key factor marking cells for competition, distinct from its effects on global translation, which only trigger cell competition when Xrp1 is induced (Figure 11).

### Transcriptional regulation of cell competition

We confirm that Xrp1 is a sequence-specific transcriptional activator, and propose that direct transcriptional targets of Xrp1 predispose *Rp^+/-^* cells, and other genotypes, to elimination by wild-type cells (Figure 11). Expression of several hundred single copy genes is regulated by Xrp1 in Rp mutant cells, and we report here that expression of some transposable elements is affected in addition, whose potential contribution to cell competition might also be interesting (Figure 10P, Figure 10—figure supplement 3, Supplementary file 1). One or more of these transcriptional targets may lead to competitive interactions with wild-type cells.

These Xrp1 targets include genes that also contribute to oxidative stress responses, such as GstD genes, which has previously led to the suggestion that an oxidative stress response is responsible for cell competition (Kucinski et al., 2017; Baumgartner et al., 2021; Recasens-Alvarez et al., 2021). Because the oxidative stress reporter used in previous studies is probably activated in *Rp^+/-^* cells by direct Xrp1-binding, and not by the Nrf2-dependent ARE site, it is not now certain whether *Rp^+/-^* cells experience oxidative stress or Nrf2 activity (Figure 10). An alternative explanation of cell competition in response to Nrf2 over-expression (Kucinski et al., 2017) could be induction of Xrp1 expression by Nrf2 (Langton et al., 2021).

### Xrp1 as a central orchestrator of cell competition

Our results reveal the central importance of Xrp1 as the driver of cell competition (Figure 11). Far from being expressed specifically in *Rp* mutants, we now find that Xrp1 is induced by multiple challenges, not only to ribosome biogenesis, such as by depletion of the polI cofactor TAF1B or LSU maturation factor eIF6, but also challenges to ribosome function, both at the levels of initiation or elongation, all leading to cell competition and to Xrp1-dependent eIF2α phosphorylation (Figure 11).

Had we not evaluated Xrp1 expression and function in PPP1R15-depleted cells, we would have concluded that eIF2α phosphorylation was the likely downstream effector of competition in *Rp* mutant cells, rather than an example of another upstream stress that induces Xrp1 (Figure 11). It is becoming apparent that other triggers of cell competition, including depletion for Helicase at 25E (Hel25E), a helicase that plays roles in mRNA splicing and in mRNA nuclear export, over-expression of Nrf2, the transcriptional master regulator of the oxidative stress response, and loss of mahjong, a ubiquitin ligase implicated in planar cell polarity, all lead to Xrp1 expression (Langton et al., 2021; Ochi et al., 2021; Kumar and Baker,unpublished). Earlier models regarding these cell competition mechanisms, in which the role of Xrp1 was not recognized, may be questionable. It would be important now to check for possible activation of Xrp1 in cells with other defects affecting translation, including mutations of an eIF5A-modifying enzyme (Patel et al., 2009) and mutations of a pre-rRNA processing enzyme (Zielke et al., 2020). It would not be surprising if other conditions that lead to eIF2α phosphorylation, such as ER stress, nutrient deprivation, or viral infection (Ron and Walter, 2007; Hetz, 2012), also activate Xrp1 and are thereby marked for elimination by more normal neighbors (Figure 11). It will be particularly interesting to determine whether any of these environmental perturbations could interfere with surveillance and removal of aneuploid cells, given the potential importance for tumor surveillance (Ji et al., 2021).

## Materials and methods

**Key resources table:** 

Reagent type (species) or resource	Designation	Source or reference	Identifiers	Additional information
Gene (*Drosophila melanogaster*)	Xrp1	GenBank	FLYBASE:FBgn0261113	
Gene (*Drosophila melanogaster*)	RpS12	GenBank	FLYBASE: FBgn0286213	
Gene (*Drosophila melanogaster*)	RpS18	GenBank	FLYBASE:FBgn0010411	
Gene (*Drosophila melanogaster*)	RpL27A	GenBank	FLYBASE:FBgn0285948	
Gene (*Drosophila melanogaster*)	RpS3	GenBank	FLYBASE:FBgn0002622	
Gene (*Drosophila melanogaster*)	RpS17	GenBank	FLYBASE:FBgn0005533	
Gene (*Drosophila melanogaster*)	RpL14	GenBank	FLYBASE:FBgn0017579	
Gene (*Drosophila melanogaster*)	RpL19	GenBank	FLYBASE:FBgn0285950	
Gene (*Drosophila melanogaster*)	RpL36	GenBank	FLYBASE:FBgn0002579	
Gene (*Drosophila melanogaster*)	TAF1B	GenBank	FLYBASE:FBgn0037792	
Gene (*Drosophila melanogaster*)	PPP1R15	GenBank	FLYBASE:FBgn0034948	
Gene (*Drosophila melanogaster*)	PERK	GenBank	FLYBASE:FBgn0037327	
Gene (*Drosophila melanogaster*)	Gcn2	GenBank	FLYBASE:FBgn0019990	
Gene (*Drosophila melanogaster*)	Irbp18	GenBank	FLYBASE:FBgn0036126	
Gene (*Drosophila melanogaster*)	eIF4G	GenBank	FLYBASE:FBgn0023213	
Gene (*Drosophila melanogaster*)	eEF2	GenBank	FLYBASE:FBgn0000559	
Gene (*Drosophila melanogaster*)	eIF6	GenBank	FLYBASE:FBgn0034915	
Gene (*Drosophila melanogaster*)	copia	GenBank	FLYBASE:FBgn0013437	
Genetic reagent (*D. melanogaster*)	eEF1α1	GenBank	FLYBASE:FBgn0284245	
Genetic reagent (*D. melanogaster*)	eIF5A	GenBank	FLYBASE:FBgn0285952	
Genetic reagent (*D. melanogaster*)	Xrp1^HA^	Blanco et al., 2020		Strain maintained in Dr. Nicholas Baker’s lab.
Genetic reagent (*D. melanogaster*)	Xrp1^M2-73^ allele	Lee et al., 2018	FLYBASE:RRID:BDSC_81270	Bloomington *Drosophila* Stock Center#81,270
Genetic reagent (*D. melanogaster*)	RpS12 ^G97D^ allele	Tyler et al., 2007	FLYBASE:FBal0193403	Strain maintained in Dr. Nicholas Baker’s lab.
Genetic reagent (*D. melanogaster*)	UAS-dsRNA^Xrp1^	Perkins et al., 2015	FLYBASE:RRID:BDSC_34521	Bloomington *Drosophila* Stock Center#34,521
Genetic reagent (*D. melanogaster*)	UAS-dsRNA^Xrp1^	Dietzl et al., 2007	FLYBASE:FBti0118620	Vienna *Drosophila* Resource Center#v 107,860
Genetic reagent (*D. melanogaster*)	UAS-dsRNA^irbp18^	Perkins et al., 2015	FLYBASE:RRID:BDSC_33652	Bloomington *Drosophila* Stock Center#33,652
Genetic reagent (*D. melanogaster*)	UAS-dsRNA^w^	Perkins et al., 2015	FLYBASE:RRID:BDSC_33623	Bloomington *Drosophila* Stock Center#33,623
Genetic reagent (*D. melanogaster*)	arm-LacZ	Vincent et al., 1994	FLYBASE:FBal0040819	
Genetic reagent (*D. melanogaster*)	Ubi-GFP	Davis et al., 1995	FLYBASE:FBal0047085	
Genetic reagent (*D. melanogaster*)	M(2)56 F(mutating RpS18)	Laboratory of Y. Hiromi	FLYBASE:FBal0011916	
Genetic reagent (*D. melanogaster*)	Df(1)R194 (deleting RpL36)	Duffy et al., 1996	FLYBASE:FBab0024817	
Genetic reagent (*D. melanogaster*)	P{RpL36+}	Tyler et al., 2007	FLYBASE:FBal0193398	
Genetic reagent (*D. melanogaster*)	M{RpL19+}	Baillon et al., 2018		
Genetic reagent (*D. melanogaster*)	Df(2 R)M60E (deleting RpL19)	Baillon et al., 2018	FLYBASE:FBab0001997	
Genetic reagent (*D. melanogaster*)	hs-FLP	Struhl and Basler, 1993	FLYBASE:FBtp0001101	
Genetic reagent (*D. melanogaster*)	P{GAL4-Act5C(FRT.CD2).P}S	Pignoni and Zipursky, 1997	FLYBASE:FBti0012408	Bloomington *Drosophila* Stock Center#51,308
Genetic reagent (*D. melanogaster*)	P{neoFRT}42D	Xu and Rubin, 1993	FLYBASE:FBti0141188	Bloomington *Drosophila* Stock Center#1,802
Genetic reagent (*D. melanogaster*)	P{neoFRT}80B	Xu and Rubin, 1993	FLYBASE:FBti0002073	Bloomington *Drosophila* Stock Center#1988
Genetic reagent (*D. melanogaster*)	P{neoFRT}82B	This study	FLYBASE:FBti0002074	Viable line derived from Bloomington Drosophila Stock Center lines BL5188 and BL30555
Genetic reagent (*D. melanogaster*)	UAS-dsRNA^TAF1B^	Perkins et al., 2015	RRID:BDSC_61957	Bloomington *Drosophila* Stock Center#61,957
Genetic reagent (*D. melanogaster*)	UAS-dsRNA^TAF1B^	Dietzl et al., 2007	FLYBASE:FBti0118760	Vienna *Drosophila* Resource Center#v105873
Genetic reagent (*D. melanogaster*)	UAS-dsRNA^eIF6^	Dietzl et al., 2007	FLYBASE:FBti0116845	Vienna *Drosophila* Resource Center#v108094
Genetic reagent (*D. melanogaster*)	UAS-dsRNA^eIF4G^	Dietzl et al., 2007	FLYBASE:FBti0095456	Vienna *Drosophila* Resource Center#v17002
Genetic reagent (*D. melanogaster*)	UAS-dsRNA^eIF5A^	Dietzl et al., 2007	FLYBASE:FBti0121478	Vienna *Drosophila* Resource Center#v101513
Genetic reagent (*D. melanogaster*)	UAS-dsRNA^eEF2^	Dietzl et al., 2007	FLYBASE:FBti0117284	Vienna *Drosophila* Resource Center#v107268
Genetic reagent (*D. melanogaster*)	UAS-dsRNA^eEF1α1^	Dietzl et al., 2007	FLYBASE:FBti0121842	Vienna *Drosophila* Resource Center#v104502
Genetic reagent (*D. melanogaster*)	UAS-dsRNA^PERK^	Dietzl et al., 2007	FLYBASE:FBti0141304	Vienna *Drosophila* Resource Center# v110278
Genetic reagent (*D. melanogaster*)	UAS-dsRNA^PERK^	Dietzl et al., 2007	FLYBASE:FBti0093363	Vienna *Drosophila* Resource Center#v 16,427
Genetic reagent (*D. melanogaster*)	UAS-dsRNA^Gcn2^	Dietzl et al., 2007	FLYBASE:FBti0118018	Vienna *Drosophila* Resource Center#v103976
Genetic reagent (*D. melanogaster*)	*RpS18* mutation *M*(*2* R)*56* f	Laboratory of Y. Hiromi	FLYBASE:FBal0284387	
Genetic reagent (*D. melanogaster*)	RpS3	Burke and Basler, 1996	Flybase: FBgn0002622	
Genetic reagent (*D. melanogaster*)	*RpL27A* Df(2 L)M24F11	Marygold et al., 2007	Flybase: FBab0001492	
Genetic reagent (*D. melanogaster*)	RpS17 mutation M(3 L)67 C^4^	Morata and Ripoll, 1975	Flybase: FBal0011935	
Genetic reagent (*D. melanogaster*)	en-Gal4	Neufeld et al., 1998	RRID:BDSC_6356	
Genetic reagent (*D. melanogaster*)	UAS-S65T-GFP	FBrf0086268	FBtp0001403	
Genetic reagent (*D. melanogaster*)	P[GAL4-Act5C(FRT.CD2).P]S	FBrf0221941	FBti0012408	
Genetic reagent (*D. melanogaster*)	P[UAS-His-RFP]3	FBrf0221941	FBti0152909	
Antibody	anti-active-Dcp1 (rabbit polyclonal)	Cell Signalling Technology	Cat #9,578RRID:AB_2721060	(1:100)
Antibody	anti-XRP1(short)(rabbit polyclonal)	Francis et al., 2016		(1:200)
Antibody	antiphospho-RpS6(rabbit polyclonal)	Romero-Pozuelo et al., 2017		(1:200)
Antibody	anti-p62 (rabbit polyclonal)	Pircs et al., 2012		(1:300)
Antibody	anti-phospho-eIF2α (rabbit polyclonal)	Thermo Fisher Scientific	Cat #44–728 GRRID:AB_2533736	(1:200)
Antibody	anti-phospho-eIF2α (D9G8)(rabbit monoclonal)	Cell Signaling Technology	Cat #D9G8#3398 RRID:AB_10829234	(1:200)
Antibody	anti-HATag (mouse monoclonal)	Cell Signalling Technology	Cat #2,367RRID:AB_10691311	(1:100)
Antibody	anti-beta galactosidase (mAb40-1a) (mouse monoclonal)	DSHB	RRID: AB_2314509	(1:100)
Antibody	anti-Mouse IgG, Cy2(goat monoclonal)	Jackson Immunoreseach	Cat #115-225-166 RRID:AB_2338746	(1:200)
Antibody	anti-Mouse IgG, Alexa Fluor 555(goat polyclonal)	Thermo Fischer Scientific	Cat #A28180 RRID:AB_2536164	(1:200)
Antibody	anti-Mouse IgG, Alexa Fluor 647(Goat polyclonal)	Thermo Fischer Scientific	Cat #A-21235 RRID:AB_2535804	(1:200)
Antibody	anti-Mouse IgG, Alexa Fluor 488(Goat polyclonal)	Thermo Fischer Scientific	Cat #A-11001RRID:AB_2534069	(1:400)
Antibody	anti-Rabbit Cy3,(Goat polyclonal)	Thermo Fischer Scientific	Cat #A-21244 RRID:AB_2535812	(1:200)
Antibody	anti-Rabbit IgG, Alexa Fluor 555(Goat polyclonal)	Thermo Fischer Scientific	Cat #A21429 RRID:AB_2535850	(1:300)
Antibody	anti-Rabbit IgG, Alexa Fluor 647(Goat polyclonal)	Thermo Fischer Scientific	Cat #A-21244 RRID:AB_2535812	(1:200)
Antibody	anti-Guinea Pig Cy5(Donkey polyclonal)	Jackson Immunoresearch	Cat #706-175-148RRID:AB_2340462	(1:200)
Antibody	Anti-rRNA (mouse monoclonal Y10b)	Thermo Fisher ScientificLerner et al., 1981	Cat #MA1-13017RRID:AB_10979967	(1:100)
Antibody	anti-dRpS12 (guinea-pig polyclonal)	Kale et al., 2018		(1:100)
Antibody	rabbit anti-hRpL10Ab(rabbit polyclonal)	Sigma-Aldrich	Cat #SAB1101199; RRID: AB_10620774	(1:200)
Antibody	anti-Rack1(rabbit monoclonal)	Cell Signalling Technology	Cat #D59D5RRID:AB_10705522	(1:100)
Antibody	anti-RpS9(rabbit monoclonal)	Abcam	Cat #ab117861 RRID:AB_10933850	(1:100)
Antibody	Anti-RpL9(rabbit monoclonal)	Abcam	Cat #ab50384RRID:AB_882391	(1:100)
commercial assay, kit	Maxiscript T7 Transcription kit	Ambion	Cat #AM1312	
other	ULTRAhyb-Oligo buffer	Ambion	Cat #AM8663	
commercial assay, kit	Click-iT Plus OPP Alexa Fluor 594 or 488 Protein Synthesis Assay Kit	Thermo Fisher Scientific	Cat #C10457	
Chemical compound, drug	Biotin-16-UTP	Roche	Cat #11388908910	
Chemical compound, drug	RNA Sample Loading Buffer	Sigma-Aldrich	Cat #R4268-5VL	
Chemical compound, drug	Heat inactivated Fetal Bovine Serum	Gibco	Cat #10082139	
Chemical compound, drug	Schneider’s *Drosophila* Medium	Gibco	Cat #21720024	
Chemical compound, drug	Trizol	Ambion	Cat #15596–026	
Chemical compound, drug	Odyssey Blocking buffer (PBS)	Li-COR	Cat #927–40003	
sequence-based reagent	18 S probe_Forward	Lee et al., 2018	Invitrogen	GGTGCTGAAGCTTATGTAGC
sequence-based reagent	18 S probe_Reverse	Lee et al., 2018	Invitrogen	TAATACGACTCACTATAGGGAGACAAAGGGCA GGGACG
sequence-based reagent	5.8 S probe_Forward	Lee et al., 2018	Invitrogen	GCTTATATGAAACTAAGACATTTCG
sequence-based reagent	5.8 S probe_Reverse	Lee et al., 2018	Invitrogen	TAATACGACTCACTATAGGGTACATAAC AGCAT GGACTGC
sequence-based reagent	ITS2 probe_Forward	This study	Invitrogen	5’- CTTTAATTAATTTTATAGTGCTGCTTGG-3’
sequence-based reagent	ITS2 probe_reverse	This study	Invitrogen	5’- TAATACGACTCACTATAGGGTTGT ATATAACTTTATCTTG-3’
sequence-based reagent	28 S probe_Forward	This study	Invitrogen	5’-GCAGAGAGATATGGTAGATGGGC –3’
sequence-based reagent	28 S probe_reverse	This study	Invitrogen	5’- TAATACGACTCACTATAGGGTTCCAC AATTGGCTACGTAACT-3’
sequence-based reagent	ITS1 probe_Forward	This study	Invitrogen	5’- GGAAGGATCATTATTGTATAATATC-3’
sequence-based reagent	ITS1 probe_Reverse	This study	Invitrogen	5’- TAATACGACTCACTATAGGGATG ATTACCACACATTCG-3’
sequence-based reagent	7SL probe_Forward	This study	Invitrogen	5’- TCGACTGGAAGGTTGGCAGCTTCTG-3’
sequence-based reagent	7SL probe_Reverse	This study	Invitrogen	5’- TAATACGACTCACTATAGGGATTGTGG TCCAACCATATCG-3’
Other	VECTASHIELD antifade mounting medium	Vector Laboratories	Cat #H-1000	
Other	Nuclear Mask reagent	Thermo Fisher Scientific	Cat #H10325	
Cell line	S2-DGRC	*Drosophila* Genomics Resource Center (NIH Grant 2P40OD010949)	FLYBASE: FBtc0000006 RRID:CVCL_TZ72	Stock #6(*D. melanogaster* embryonic cell line)
Other	Dual-Luciferase Reporter Assay System	Promega	Cat #E1910	
Other	TransIT-2020 Transfection Reagent	Mirus Bio	Cat #MIR 5404	
sequence-based reagent	Xrp target 1+ strand	This study		TCGAGATTGCACAACGCTCATTGCAC AACGTTCATTGCACAACGGCAATTGCACAACG
sequence-based reagent	Xrp target 1 - strand	This study		TCGACGTTGTGCAATTGCCGTTGTGCAATG AACGTTGTGCAATGAGCGTTGTGCAATC
sequence-based reagent	Xrp target 2+ strand	This study		TCGAGCATGATGAAATAACATGCTCCATGA TGAAATAACATGTTCCATGATGAAATAACA TGGCACATGATGAAATAACATG
sequence-based reagent	Xrp target 2 - strand	This study		TCGACATGTTATTTCATCATGTGCCATGTTA TTTCATCATGGAACATGTTATTTCATCATG GAGCATGTTATTTCATCATGC
sequence-based reagent	Xrp target 3+ strand	This study		TCGAGATTACATCATGCTCATTACATC ATGTTCATTACATCATGGCAATTACATCATG
sequence-based reagent	Xrp target 3 - strand	This study		TCGACATGATGTAATTGCCATGATGTAATG AACATGATGTAATGAGCATGATGTAATC
sequence-based reagent	Xrp trgt 1+ strand shuffled	This study		TCGAGTGACAACTCAGCTCTGACAACTCAGT TCTGACAACTCAGGCATGACAACTCAG
sequence-based reagent	Xrp trgt 1 - strand shuffled	This study		TCGACTGAGTTGTCATGCCTGAGTTGTCAGAA CTGAGTTGTCAGAGCTGAGTTGTCAC
sequence-based reagent	Xrp trgt 2+ strand shuffled	This study		TCGAGTTCAAATCAATAGGAAGCTCTTCAAATCA ATAGGAAGTTCTTCAAATCAATAGGAAGGCATTC AAATCAATAGGAAG
sequence-based reagent	Xrp trgt 2 - strand shuffled	This study		TCGACTTCCTATTGATTTGAATGCCTTCCTATT GATTTGAAGAACTTCCTATTGATTTGAAGAGC TTCCTATTGATTTGAAC
recombinant DNA reagent	pGL3-Promoter Vector	Promega	Cat #E1761	
recombinant DNA reagent	pAct5.1/V5-His C vector	Thermo Fischer Scientific	Cat #V411020	
recombinant DNA reagent	pIS1 plasmid	Addgene	Cat #12,179	
recombinant DNA reagent	pUAST vector	Brand and Perrimon, 1993	FLYBASE:FBmc0000383	*Drosophila* Genomics Resource Center#1,000
recombinant DNA reagent	pGL3-Rluc	This study		See Materials and Methods; Dr. Nicholas Baker’s lab
recombinant DNA reagent	p-GL3-H-T1	This study		See Materials and Methods; Dr. Nicholas Baker’s lab
recombinant DNA reagent	p-GL3-H-T2	This study		See Materials and Methods; Dr. Nicholas Baker’s lab
recombinant DNA reagent	p-GL3-H-T3	This study		See Materials and Methods; Dr. Nicholas Baker’s lab
recombinant DNA reagent	p-GL3-H-T1S	This study		See Materials and Methods; Dr. Nicholas Baker’s lab
recombinant DNA reagent	p-GL3-H-T2S	This study		See Materials and Methods; Dr. Nicholas Baker’s lab
recombinant DNA reagent	pGL3-X-T1	This study		See Materials and Methods; Dr. Nicholas Baker’s lab
recombinant DNA reagent	pGL3-X-T2	This study		See Materials and Methods; Dr. Nicholas Baker’s lab
recombinant DNA reagent	pGL3-X-T3	This study		See Materials and Methods; Dr. Nicholas Baker’s lab
recombinant DNA reagent	pGL3-X-T1S	This study		See Materials and Methods; Dr. Nicholas Baker’s lab
recombinant DNA reagent	pGL3-X-T2S	This study		See Materials and Methods; Dr. Nicholas Baker’s lab

### Experimental animals

Fly strains were generally maintained at 25 °C on yeast cornmeal agar. Yeast-glucose medium was generally used for mosaic experiments (Sullivan et al., 2000). Sex of larvae dissected for most imaginal disc studies was not differentiated.

### Clonal analysis

Genetic mosaics were generated using the FLP/FRT system using inducible heat-shock FLP (hsFLP) transgenic strains. For making clones through mitotic recombination using inducible heat-shock FLP (hsFLP), larvae of *Rp ^±^*genotypes were subjected to 10–20 min heat shock at 37 °C, 60 ± 12 hours after egg laying (AEL) and dissected 72 hr later. For making clones by excision of a FRT cassette, larvae were subjected to 10–30 min heat shock at 37 °C (details in Supplementary file 2), 36 ± 12 AEL for wild type background or 60 ± 12 hr AEL for *Rp ^±^*background, and dissected 72 hr later.

### *Drosophila* stocks

Full genotypes for all the experiments are listed in Supplementary file 2. The following genetic strains were used: UAS-PPP1R15 (BL76250), UAS-PERK-RNAi (v110278 and v16427), UAS-Gcn2-RNAi (v103976), TRE-dsRED, P[GAL4-Act5C(FRT.CD2). P]S, P[UAS-His-RFP]3 (isolated from BL51308), UAS-TAF1B-RNAi (BL61957 and v105783), UAS-PPP1R15-RNAi (v107545 and BL 33011), UAS-w-RNAi (BL33623), UAS-CG6272-RNAi (BL33652), UAS-Xbp1-EGFP (BL60731), UAS-eIF4G-RNAi (v17002), UAS-eEF2-RNAi (v107268), UAS-eEF1α1-RNAi (v104502), UAS-eIF5Α-RNAi (v101513), UAS-eIF6-RNAi (v108094), UAS-Bsk^DN^ (BL9311). Other stocks are described in Lee et al., 2018.

### Immunohistochemistry and antibody labeling

For most antibody labeling, imaginal discs were dissected from late 3rd instar larvae in 1xPBS buffer and fixed in 4% formaldehyde in 1 x PEM buffer (1xPEM:100 mM Pipes, 1 mM EGTA, 1 mM MgCl2, pH 6.9). For p-eIF2α and p-RpS6 detection, larvae were dissected in *Drosophila* S2 medium one by one and transferred immediately to fixative. Fixed imaginal discs were 3 x washed in PT (0.2% Triton X-100, 1xPBS) and blocked for 1 hr in PBT buffer (0.2% Triton X-100, 0.5% BSA, 1 x PBS). Discs were incubated in primary antibody in PBT overnight at 4 °C, washed three times with PT for 5–10 min each and incubated in secondary antibody in PBT for 3–4 hr at room temperature, and washed three times with PT for 5–10 min. After washes, samples were rinsed in 1 x PBS and the samples were incubated with the NuclearMask reagent (Thermoﬁsher, H10325) for 10–15 min at room temperature. After washing 2 x with 1 x PBS the imaginal discs were mounted in VECTASHIELD antifade mounting medium (Vector Laboratories, H-1000). In experiments that we wanted to parallel process control samples on the same tube (e.g. Figure 5C vs 5 J), we used male parents that had the genotypes hsFLP; TRE-dsRed/(PPP1R15 or Xrp1RNAi or PERKRNAi); act>> Gal4, UAS-GFP and cross them with females from the RNAi of interest. The genotypes in the same tube were discriminated using dsRed before the addition of the secondary antibody. We used the following antibodies for staining: rabbit anti-phospho-RpS6 at 1:200 (1:200) (Romero-Pozuelo et al., 2017), rabbit anti-p62 (Pircs et al., 2012), rabbit anti-phospho-eIF2α at 1:100 (Thermoﬁsher, 44–728 G, and Cell Signaling Technologies), rabbit anti-Xrp1 at 1:200 (Francis et al., 2016), mouse anti-b-Galactosidase (J1e7, DSHB), rabbit anti-GFP, rabbit anti-active-Dcp1 (Cell Signaling Techonology Cat#9578, 1:100), Y10b(1:100)(Thermoﬁsher, MA1-13017), RpL9(1:100)(Abcam, ab50384),rabbit-anti-Rack1 (1:100) (Cell Signalling, D59D5), rabbit anti-hRpL10Ab (1:100) (Sigma, Cat# SAB1101199). Secondary Antibodies were Cy2- and Cy5- conjugates (Jackson Immunoresearch) or Alexa Fluor conjugates (Thermofisher). Previous experiments established that significant results could be obtained from five replicates, although many more were imaged in most cases. No calculations regarding sample sizes were performed. No outliers or divergent results were excluded from analysis.

### Image acquisition and processing

Confocal laser scanning images were acquired with a Leica Laser scanning microscope SP8 using 20 x and 40 x objectives. Images were processed using Image J1.44j and Adobe Photoshop CS5 Extended. Thoracic bristle images were recorded using Leica M205 FA and Leica Application Suite X.

### Measurement of in vivo translation

Translation was detected by the Click-iT Plus OPP Alexa Fluor 594 or 488 Protein Synthesis Assay Kit (Thermoﬁsher, C10457) as described earlier (Lee et al., 2018). Larvae were inverted in Schneider’s *Drosophila* medium (containing 10% heat inactivated Fetal Bovine Serum, Gibco) and transferred in fresh medium containing 1:1000 (20 µM) of Click-iT OPP reagent. Samples were incubated at room temperature for 15 min and rinsed once with PBS. The samples were fixed in 4% formaldehyde in 1 x PEM buffer (100 mM Pipes, 1 mM EGTA, 1 mM MgCl2) for 20 min, washed once with 1 x PBS and subsequently washed with 0.5% Triton in 1 x PBS for 10 min and then incubated for 10 min with 3% BSA in 1 x PBS. The Click reaction took place in the dark at room temperature for 30 min. Samples were washed once with the rinse buffer of the Click reaction kit, 2 min with 3% BSA in 1 x PBS, incubated for 1 hr at room temperature with PBT (1 x PBS, 0.2% Triton, 0.5% BSA) and after that incubated overnight with the primary antibodies at 4°C. Samples were washed 3 x with PT buffer (1 x PBS, 0.2% Triton) and the secondary antibody was added for 2 hr in room temperature. After 3 x washes with PT and 1 x with 1 x PBS, the samples were incubated with the Nuclear Mask reagent (1:2000) of the Click-iT kit for 30 min. After washing 2 x with 1 x PBS the imaginal discs were mounted in Vectashield. Confocal laser scanning images were acquired with a Leica Laser scanning microscope SP8.

### Northern analysis

RNA extraction, northern blotting procedures, and 18 S, 5.8 S, tubulin and actin probeswere as described (Lee et al., 2018). Previous studies established that significant results could be obtained from three biological replicates. A biological replicate represents an independent RNA isolation, gel, and blot experiment.

The following primers were used to amplify the new probes in this paper:

ITS2 probe:

5’- CTTTAATTAATTTTATAGTGCTGCTTGG-3’
5’- TAATACGACTCACTATAGGGTTGTATATAACTTTATCTTG-3’
28 S probe:
5’-GCAGAGAGATATGGTAGATGGGC -3’
5’- TAATACGACTCACTATAGGGTTCCACAATTGGCTACGTAACT-3’

ITS1 probe:

5’- GGAAGGATCATTATTGTATAATATC-3’
5’- TAATACGACTCACTATAGGGATGATTACCACACATTCG-3’
7SL probe:
5’- TCGACTGGAAGGTTGGCAGCTTCTG-3’
5’- TAATACGACTCACTATAGGGATTGTGGTCCAACCATATCG-3’

### Plasmid cloning

All the new plasmids described below were confirmed by DNA sequencing.

Control *Renilla* luciferase plasmid: The pGL3-Promoter Vector (Promega) was modified by replacement of the SV40 promoter by the *Drosophila* actin promoter from the pAct5.1/V5-His C vector (Thermo Scientific), and the firefly luciferase coding sequence by the Renilla luciferase (RLuc) coding sequence from the pIS1 plasmid (Addgene), yielding the pGL3-Rluc plasmid.

*Firefly* luciferase plasmids: The SV40 core promoter of the pGL3-Promoter Vector was by hsp70 and Xrp1core promoters, amplified from the pUAST vector (*Drosophila* Genomics Resource Center) and from wild-type *Drosophila* genomic DNA respectively, using primers with XhoI and HindIII restriction sites. The resulting pGL3-H and pGL3-X plasmids were digested with Xho1 for insertion of annealed complementary oligonucleotides containing multiple copies of Target 1, Target 2, Target 3, or shuffled Target one or Target two sequences, resulting in the p-GL3-H-T1, p-GL3-H-T2, p-GL3-H-T3, p-GL3-H-T1S, p-GL3-H-T2S, pGL3-X-T1, pGL3-X-T2, pGL3-X-T3, pGL3-X-T1S, and pGL3-X-T2S plasmids.

Inducible expression plasmids: The Xrp1 (with and without its 3’UTR sequence) and Irbp18 (CG6272) coding regions were amplified from pUAST-Xrp1-HA and pUAST-CG6272 (Blanco et al., 2020), and inserted into pMT/V5-His A (Thermo Scientific) usingXhoI and SpeI target sites, resulting in three inducible protein plasmids: pMT-Xrp1^HA^Δ3’UTR, pMT-Xrp1^HA^ and pMT-Irbp18^V5/His^. pMT-Xrp1^HA^ was not used further as it did not express Xrp1 protein in S2 cells.

### S2 cell culture and luciferase assays

*Drosophila* S2 cells from the *Drosophila* Genomics Resource Center (DGRC - stock#6) were cultured in Schneider’s medium (Thermo Scientific) supplemented with 10% Heat-Inactivated Fetal Bovine Serum (Thermo Scientific) at 25 °C following the *General procedures for maintenance of Drosophila cell lines* from the DGRC. For luciferase assays, S2 cells were plated in 24-well plates, 5 × 10^5^ cells per well. After 24 hr cells were transfected with the indicated combination of control Rluc (0.15 ng/well), protein expression (15 ng/well) and target (4.5 ng/well) plasmids using TransIT-2020 Transfection Reagent (Mirus) following the manufacturer’s instructions. After 24 hr, copper sulfate was added to a final concentration of 0.35 mM. After a further 24 hr cells were lysed and *Renilla* and *Firefly* luciferases’ activity measured with a luminometer, following the instructions from the Dual-Luciferase Reporter Assay System (Promega). *Firefly* signal was normalized to the internal *Renilla* control. Each transfection was performed in triplicate, and experiments performed independently at least three times.

### mRNA-Seq Analysis

In order to interrogate the RNA-Seq data (GSE112864 and GSE124924)(Lee et al., 2018; Ji et al., 2019) for the presence and abundance of transposons, we firstly retrieved a list of the known *Drosophila melanogaster* transposons from FlyBase (https://flybase.org/) as well as the related FASTA sequences (version r6.41) for which a dedicated Bowtie2 index was constructed. Subsequently, we realigned the RNA-Seq FASTQ files to the transposons using Bowtie2 with default parameters, while restricting the output of unaligned reads (--no-unal option) for faster later quantification. After the alignment, a raw transposon read counts table was constructed using samtools. Final quantification was obtained with RPKM transformation using the RNA-Seq sample library sizes and the lengths of each transposon.

## Data Availability

mRNA-Seq data were analyzed from datasets available from GEO with accession numbers GSE112864 and GSE124924. All other data generated or analysed during this study are included in the manuscript and supporting files. Source data files have been provided for Figure 1, Figure 2, Figure 2-figure supplement 1, Figure 8-figure supplement 4, Figure 10 and Figure 10-figure supplement 1. The following previously published datasets were used: KiparakiM BlancoJ FolgadoV JiZ KumarA RimessoG BakerN 2018RNA-seq analysis to assess transcriptional effects of Rp mutations in wing imaginal discs and their dependence on Xrp1NCBI Gene Expression OmnibusGSE112864 BakerN KiparakiM BlancoJ FolgadoV JiZ KumarA RimessoG 2019mRNA Seq analysis of *Drosophila* wing imaginal discs from Rp mutants and controls in the presence and absence of RpS12 mutations RpS12NCBI Gene Expression OmnibusGSE124924

## Additional files

Supplementary file 1. mRNA-seq data for transposable elements.

Supplementary file 2. Genotypes, numbers, and heat-shock parameters for figures.

Transparent reporting form
