# Supplementary material for: The transcription factor Xrp1 orchestrates both reduced translation and cell competition upon defective ribosome assembly or function
Source: eLife. 2022 Feb 18;11:e71705. doi: 10.7554/eLife.71705 (PMC8933008; doi:10.7554/eLife.71705)

Figure 1 source data 1

unedited northern, 7SL probe

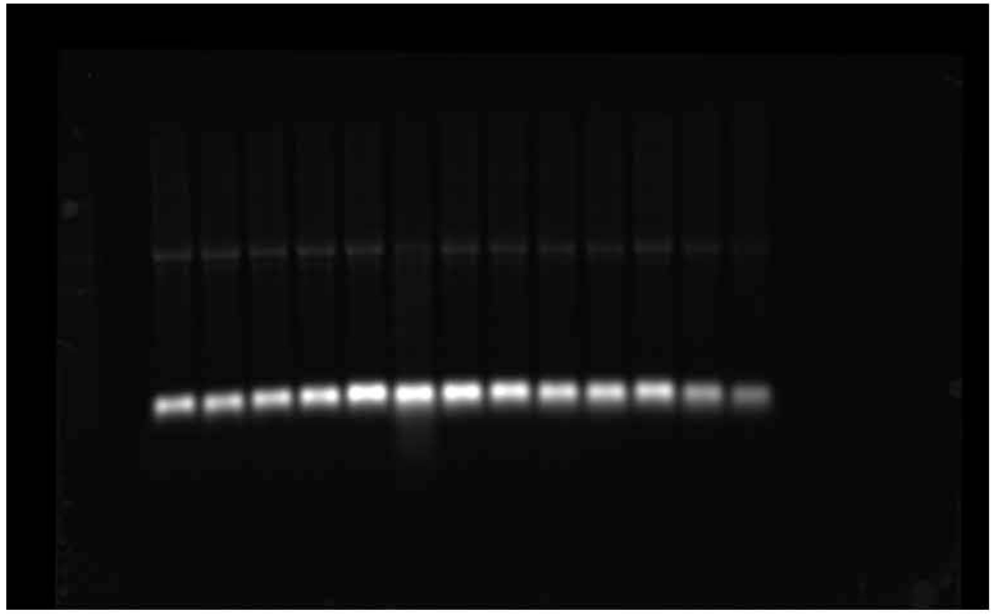

unedited northern, 5.8S probe

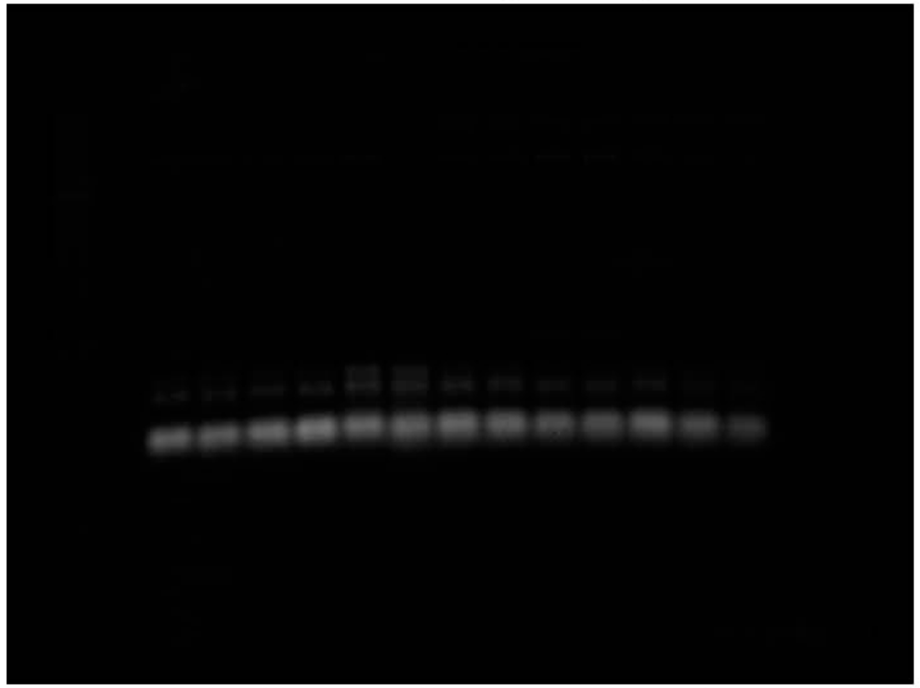

unedited northern, 18S probe

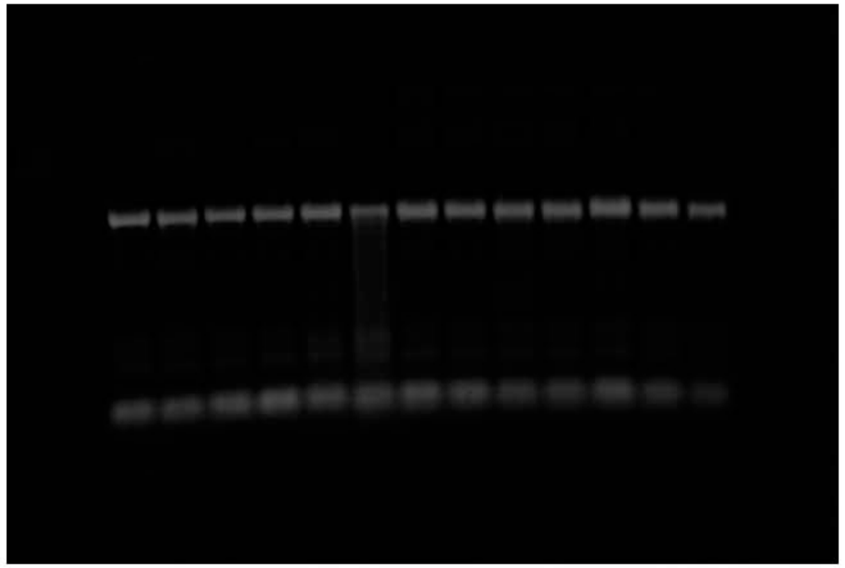

labelled northern, 7SL probe

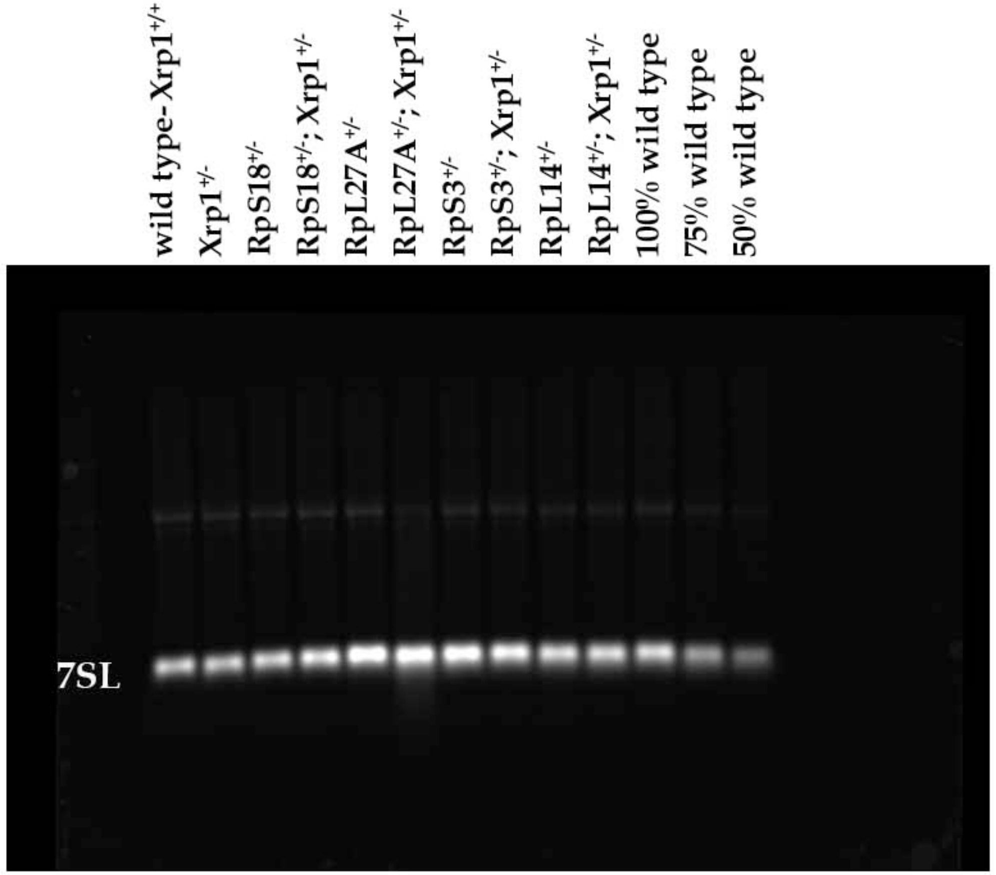

labelled northern, 5.8 probe

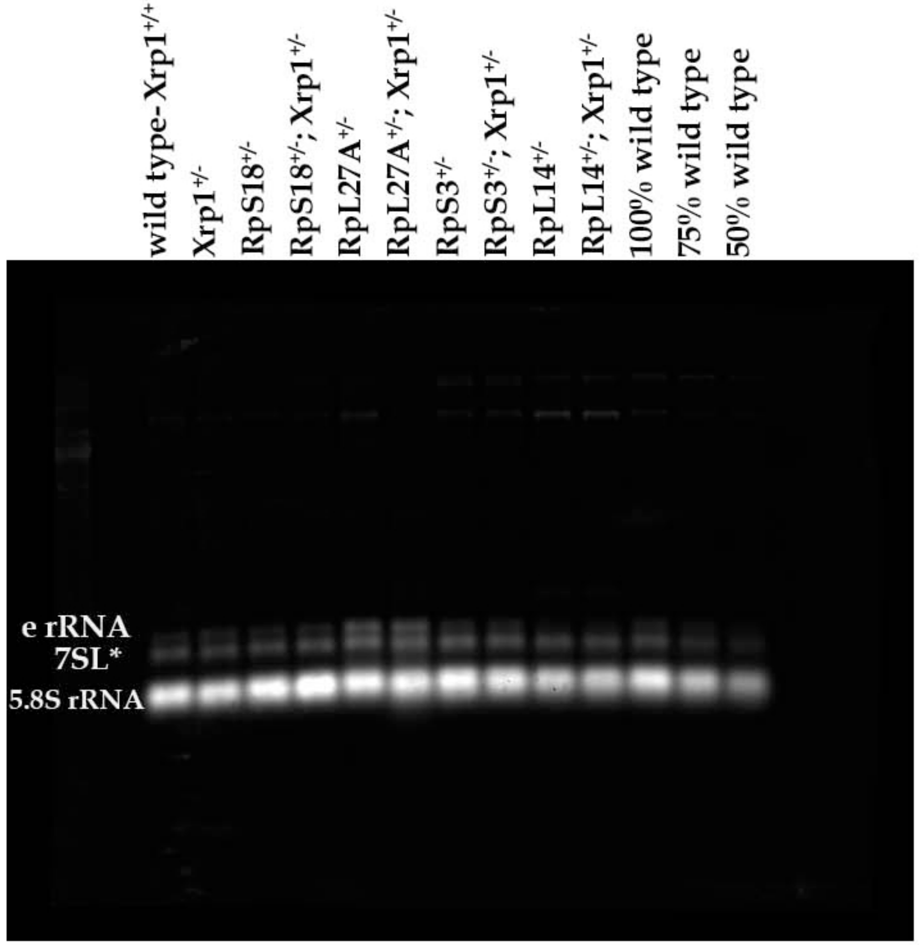

labelled northern, 18S probe

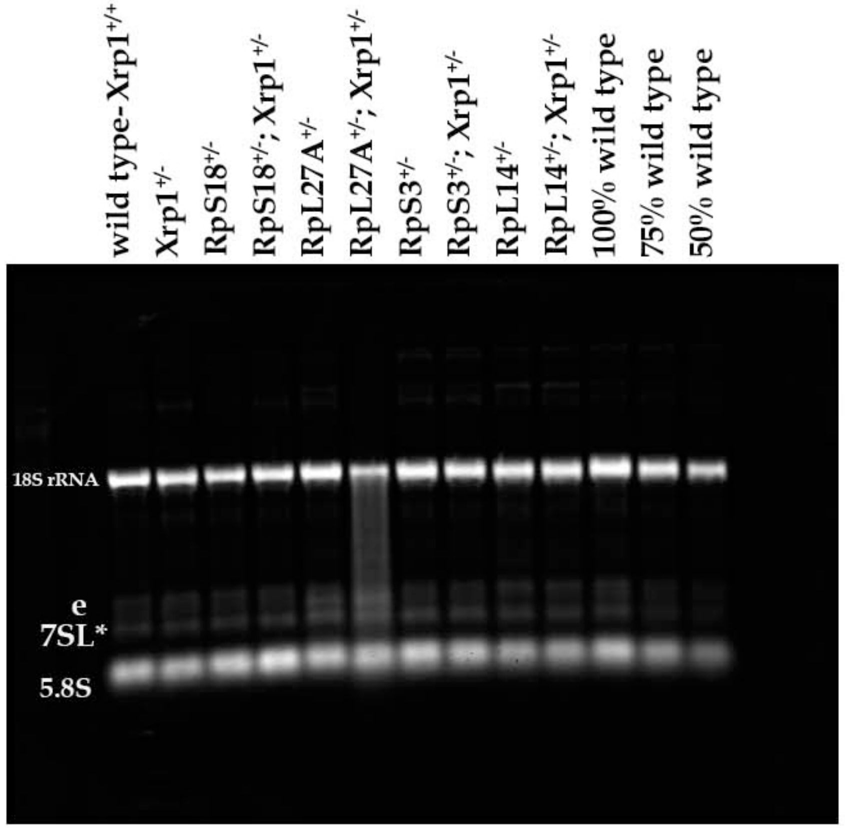

Supplement: Figure 1—source data 1. [file elife-71705-fig1-data1.pdf]
