# Supplementary material for: The transcription factor Xrp1 orchestrates both reduced translation and cell competition upon defective ribosome assembly or function
Source: eLife. 2022 Feb 18;11:e71705. doi: 10.7554/eLife.71705 (PMC8933008; doi:10.7554/eLife.71705)

Figure 2 source data file 1

unedited northern, actin probe

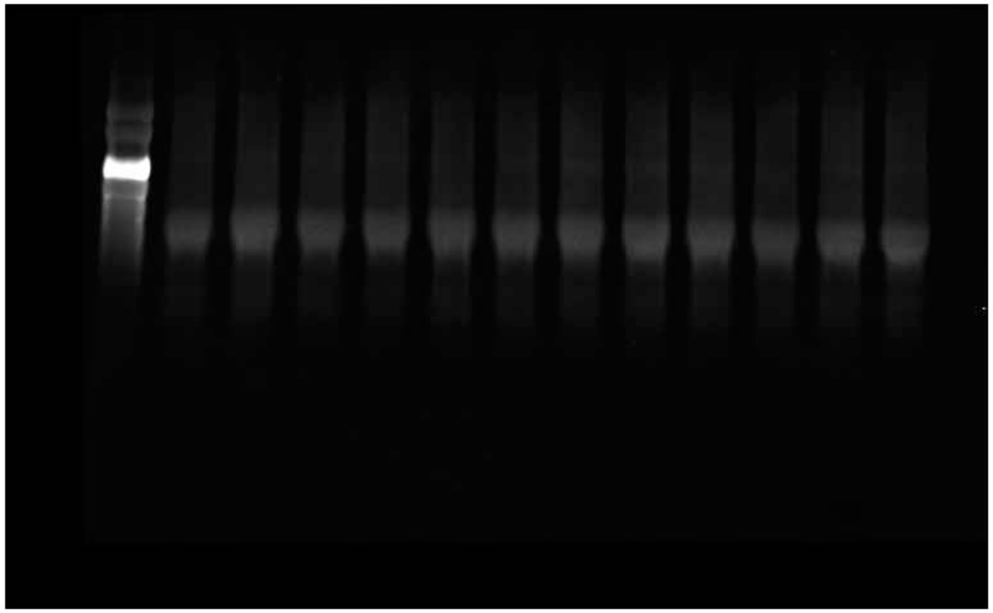

unedited northern, ITS1 probe

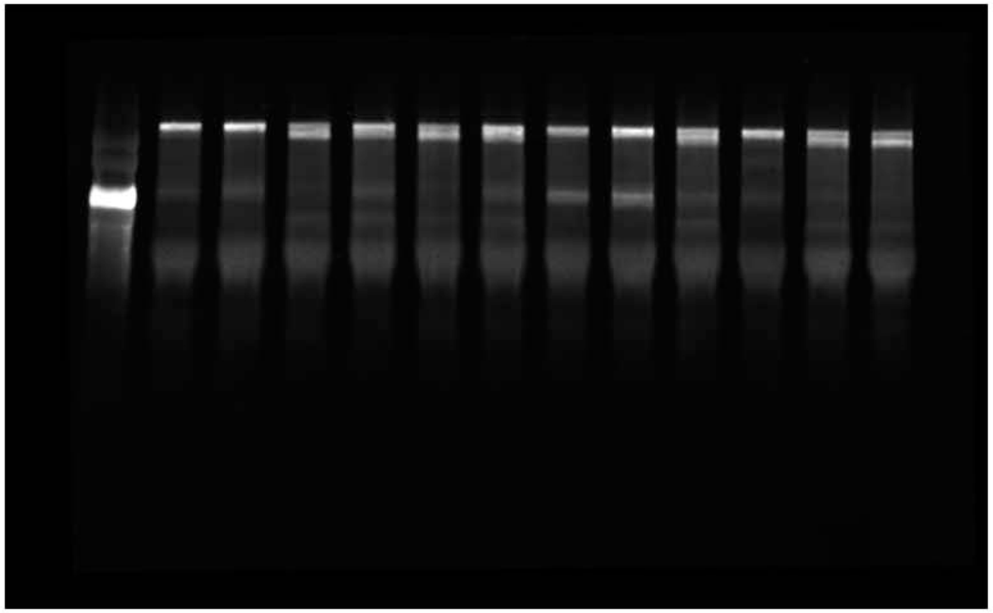

labelled northern, actin probe

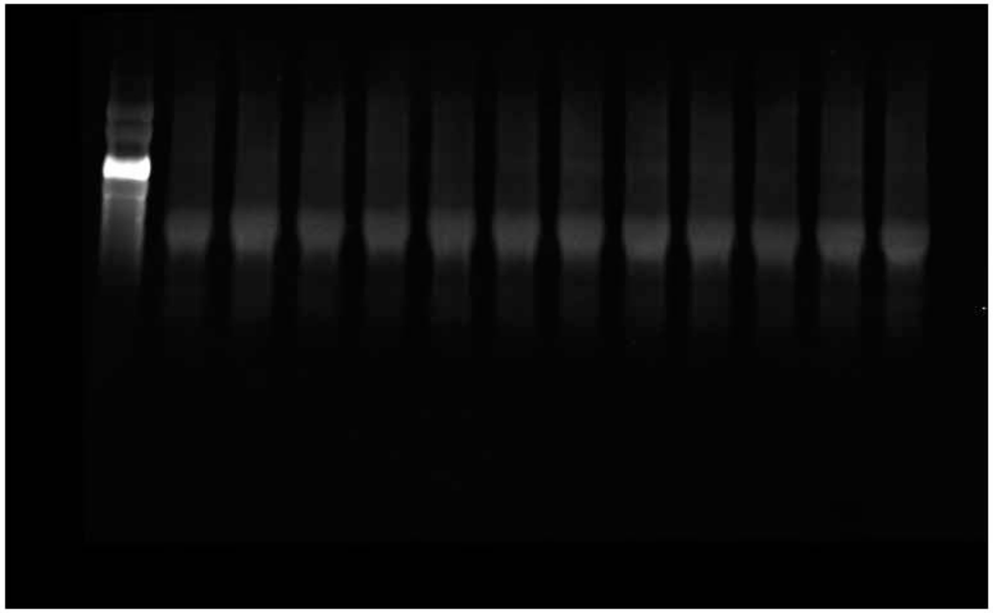

labelled northern, ITS1 probe

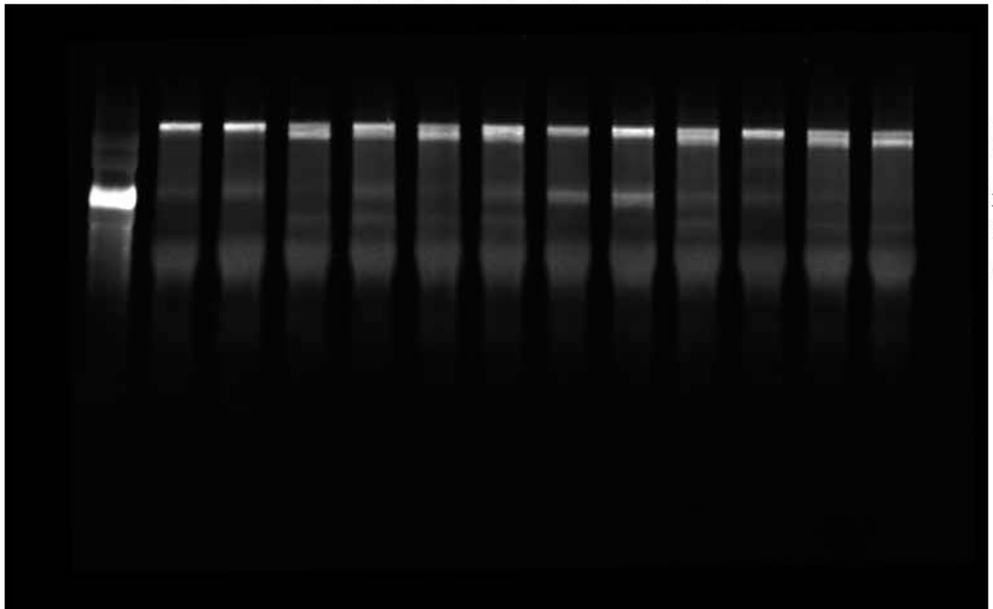

Supplement: Figure 2—source data 1. [file elife-71705-fig2-data1.pdf]
