# Supplementary material for: The transcription factor Xrp1 orchestrates both reduced translation and cell competition upon defective ribosome assembly or function
Source: eLife. 2022 Feb 18;11:e71705. doi: 10.7554/eLife.71705 (PMC8933008; doi:10.7554/eLife.71705)

Figure 2 source data file 2

unedited northern, tubulin probe

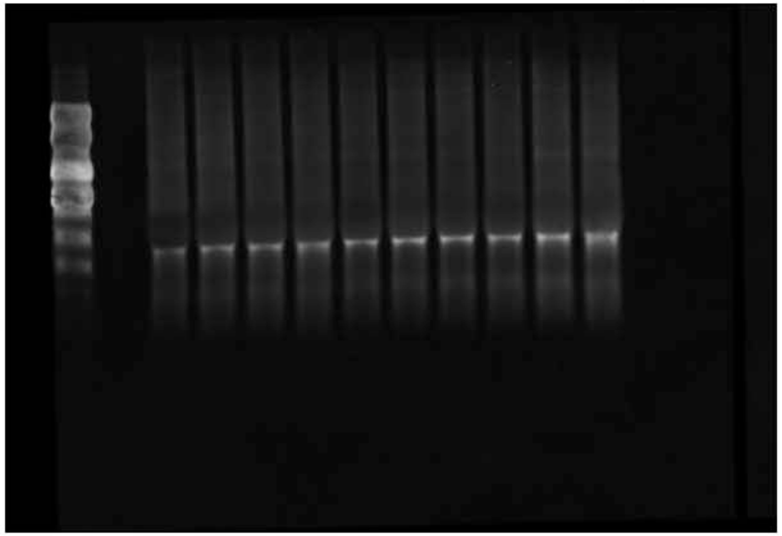

unedited northern, ITS2 probe

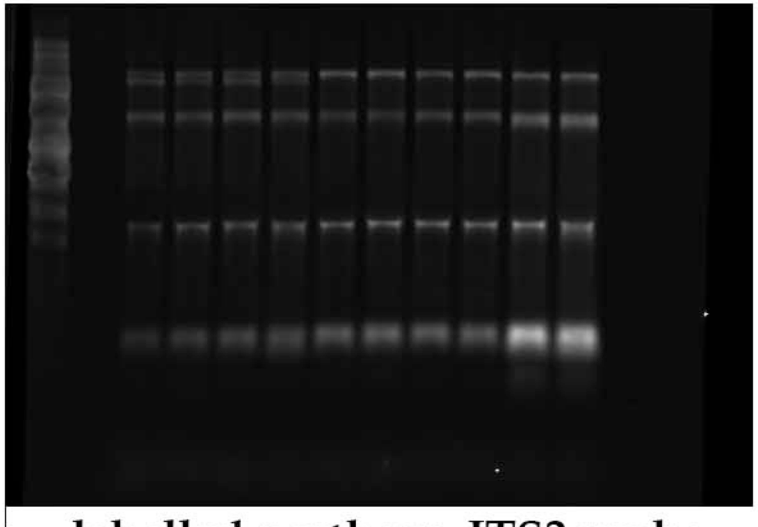

unedited northern, 18S probe

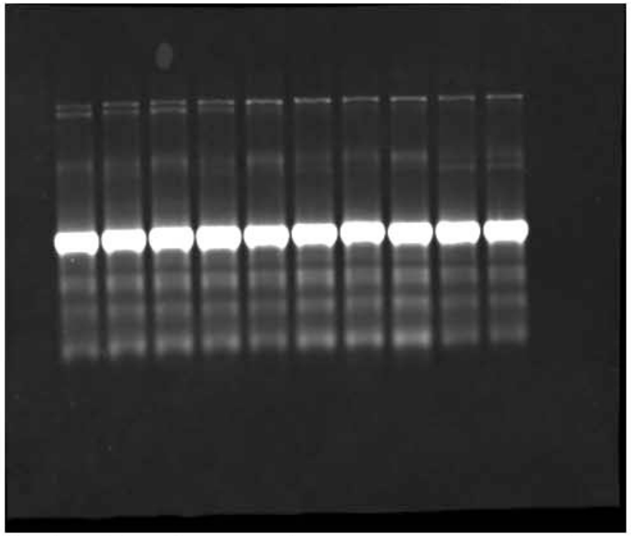

labelled northern, tubulin probe

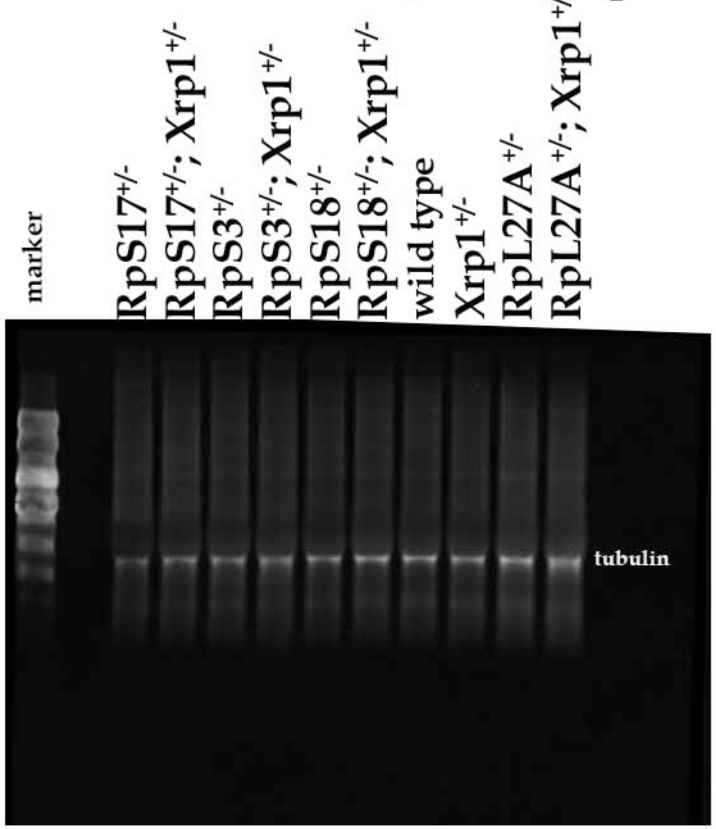

labelled northern, ITS2 probe

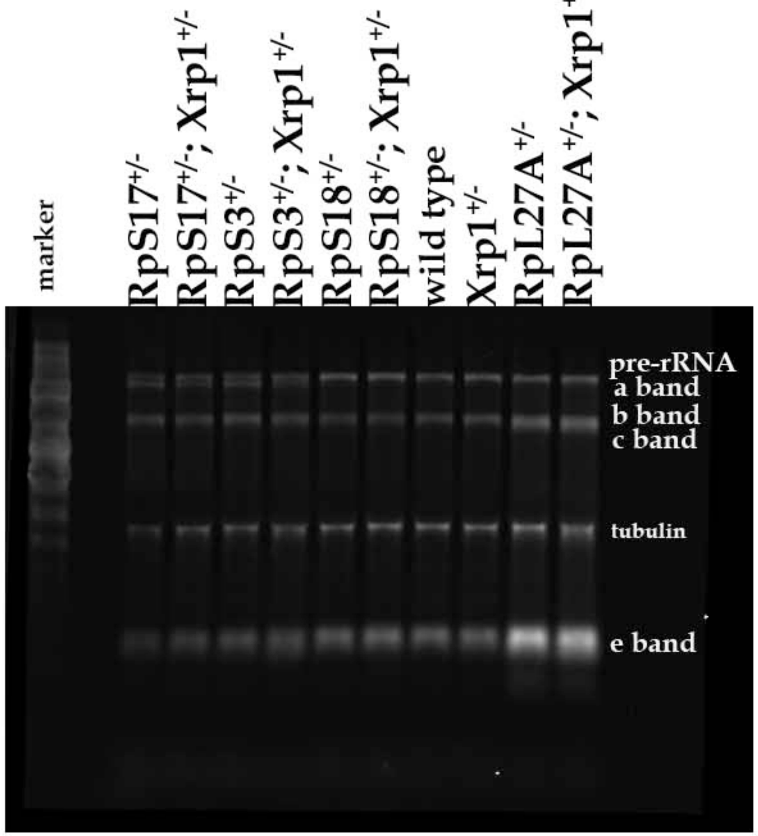

labelled northern, 18S probe

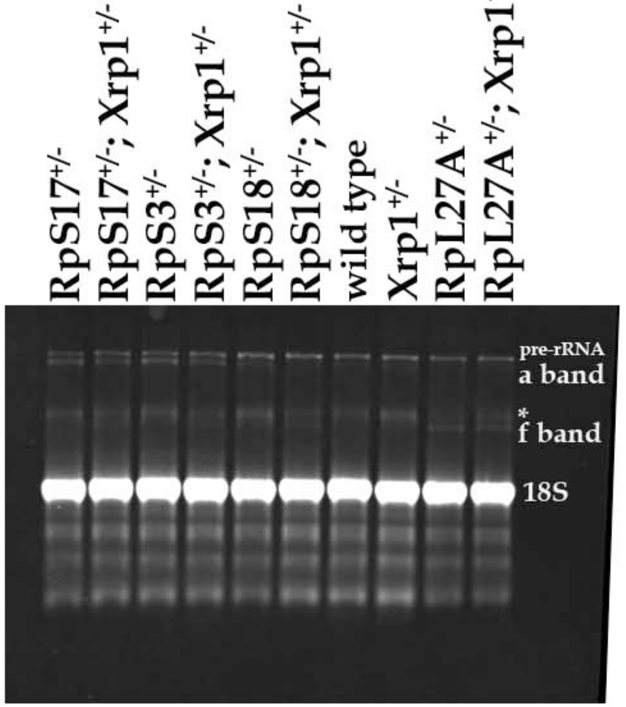

Supplement: Figure 2—source data 2. [file elife-71705-fig2-data2.pdf]
