# Supplementary material for: The transcription factor Xrp1 orchestrates both reduced translation and cell competition upon defective ribosome assembly or function
Source: eLife. 2022 Feb 18;11:e71705. doi: 10.7554/eLife.71705 (PMC8933008; doi:10.7554/eLife.71705)

Figure 2 source data file 3

unedited northern
28S probe

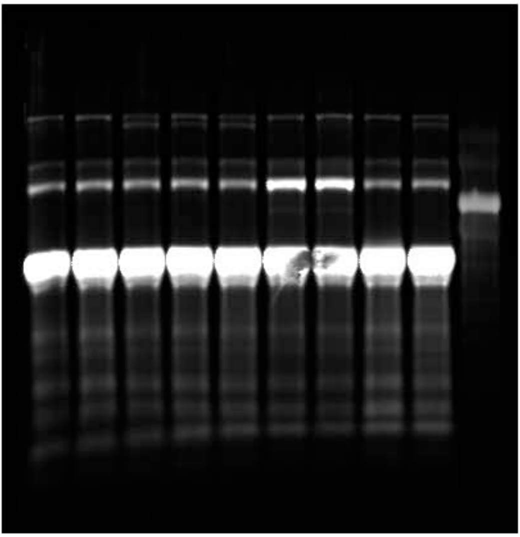

labelled northern
28S probe

wild type
$Xrp1^{+/-}$
$RpS3^{+/-}$
$RpS3^{+/-}$; $Xrp1^{+/-}$
$RpS3^{+/-}$
$RpL27A^{+/-}$
$RpL27A^{+/-}$; $Xrp1^{+/-}$

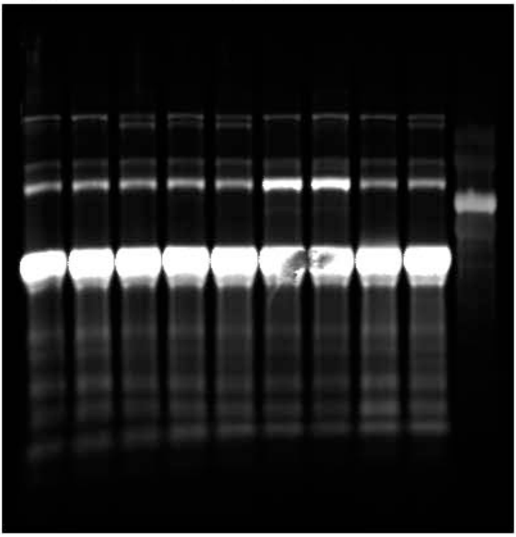

Supplement: Figure 2—source data 3. [file elife-71705-fig2-data3.pdf]
