# Supplementary material for: The transcription factor Xrp1 orchestrates both reduced translation and cell competition upon defective ribosome assembly or function
Source: eLife. 2022 Feb 18;11:e71705. doi: 10.7554/eLife.71705 (PMC8933008; doi:10.7554/eLife.71705)
Supplement: Supplementary file 2. [file elife-71705-supp2.docx]

**Supplementary Table 2:** Genotypes, heat shock times and N numbers of Figures

|  | | Heat shock time (min) | N number |  |
| --- | --- | --- | --- | --- |
| Figure 1, 2 and Figure 2-Figure supplement 1  For Northerns:  wt genotype: p{hs:FLP}/w118; p{arm:LacZ} FRT80B/+  Xrp1/+ genotype: p{hs:FLP}/w118; FRT82B *Xrp1^M2^*^-73^/+  RpS3/+ genotype: p{hs:FLP}/ p{hs:FLP}; FRT42/+; FRT82 *RpS3* p{arm:LacZ} /+  RpS3/+; Xrp/+ genotype: p{hs:FLP}/ p{hs:FLP}; FRT82 *RpS3* p{arm:LacZ} /FRT82B *Xrp1^M2^*^-73^  RpS17/+ genotype: p{hs:FLP}/ p{hs:FLP}; FRT42/+; FRT80 *RpS17* p{arm:LacZ} /+  RpS17/+; Xrp/+ genotype: p{hs:FLP}/ p{hs:FLP}; FRT80 *RpS17* p{arm:LacZ} /FRT82B *Xrp1^M2^*^-73^  *RpL27A*/+ genotype: p{hs:FLP}/ p{hs:FLP}; *RpL27A*- p{arm:LacZ} FRT40/+; FRT80B/+  *RpL­27A*/+; *Xrp*/+ genotype: p{hs:FLP}/ p{hs:FLP}; *RpL27A*- p{arm:LacZ} FRT40/+; FRT82B *Xrp1^M2^*^-73^/+  *RpS18*/+ genotype: p{hs:FLP}/ p{hs:FLP}; FRT42 *RpS18* p{ubi:GFP} /+; FRT80B/+  *RpS18*/+; Xrp/+ genotype: p{hs:FLP}/ p{hs:FLP}; FRT42 *RpS18* p{ubi:GFP} /+; FRT82B *Xrp1^M2^*^-73^/+  *L14*^+/-^ genotype: p{hs:FLP}/ p{hs:FLP}; FRT42/+; *RpL14*^1^ /+,  *L­14*^+/-^; *Xrp1*^+/-^ genotype: p{hs:FLP}/ p{hs:FLP}; FRT42/+; *RpL14^1^* / FRT82B *Xrp1^M2^*^-73^ | | No hs | 3-4 biological replicates |  |
| Figure 1F, H, Figure 1-Figure supplement 1A, Figure 1-Figure supplement 2A: p{hs:FLP}/ p{hs:FLP}; RpL27A^-^ p{arm:LacZ} FRT40/FRT40 | 20 | Figure 1F, Figure supplement 1A: Y10b: N=4  Figure 1H RpL9: N=6 (reduced always)  Figure 1H’’: RpS12 (N=2 reduced and N=no effect)  Figure supplement 2A: RpL10Ab: N=5 (always reduced) | | |
| Figure 1G, Figure 1-Figure supplement 1B,C: p{hs:FLP}/ p{hs:FLP}; FRT82 *RpS3* p{arm:LacZ} /FRT82B | 20 | Figure 1G, Figure supplement 1B,C: Y10b: N=3 | | |
| Figure 1I, Figure 1-Figure supplement 2B : p{hs:FLP}/ p{hs:FLP}; FRT42 *RpS18* p{Ubi:GFP}/FRT42 | 20 | 1I: RpS12. N=5, RpL19: N=4  Suppl 2B: RpL10Ab. N=3 | | |
| Figure 1-Figure supplement 1D, Figure 1-Figure supplement 2D, E: p{hs:FLP}/+; *RpS17* p{arm:LacZ} FRT80B/FRT80B | 20 | Figure supplement 1D: Y10b: N=6  Figure supplement 2D: Rack1: N=5  Figure supplement 2E: RpS12: N=6  Figure supplement 2E: RpL9: N=7 | | |
| Figure 1-Figure supplement 2C: p{hs:FLP}/ p{hs:FLP}; FRT42 *RpS18* p{Ubi:GFP}/FRT42; FRT82B Xrp1^M2-73^/+ | 20 | RpL10Ab. N=5 | | |
| Figure 2E, F: p{hs:FLP}/+; UAS- RNAi^TAF1B^ /+ ;*RpS17*, act>CD2>Gal4 , UAS-GFP /+  (line: v105873) | 25±5 | 2E. Xrp1-HA: N= 12  2F. Dcp1: N=7 | | |
| Figure 2G: p{hs:FLP}/+; UAS- RNAi^TAF1B^ /+ ;act>CD2>Gal4 , UAS- GFP /+ (line: Bl 61957) | 25±5 | Xrp1: N=16 | | |
| Figure 2H: p{hs:FLP}/+; UAS-RNAi^TAF1B^/+ ;act>CD2>Gal4 , UAS- GFP /+ (line: Bl 61957) | 20 | Dcp1. N=15 (for both 61957 and v105873 lines) | | |
| Figure 2I: p{hs:FLP}/+; UAS- RNAi^TAF1B^ /UAS-RNAi^Xrp1^ ;act>CD2>Gal4 , UAS- GFP /+ (line: Bl 61957) | 20 | Dcp1.= 6  OPP. N=2 | | |
| Figure 2J: p{hs:FLP}/+; UAS- RNAi^TAF1B^ /TRE-dsRED ;act>CD2>Gal4 , UAS- GFP /+ (line: Bl 61957) (processed in parallel with 2I) | 20 | Dcp1. N=15 (for both lines) | | |
| Figure 2 Supplement 2A: : p{hs:FLP}/ p{hs:FLP}; FRT42/FRT42; Xrp1-HA/Xrp1-HA | n/a | N=4 | | |
| Figure 2 Supplement 2B: p{hs:FLP}/ p{hs:FLP}; FRT42 *RpS18* p{Ubi:GFP}/FRT42 | 30 | N=4 | | |
| Figure 2 Supplement 2C-D: p{hs:FLP}/+; *RpS17* p{Ubi:GFP} FRT80B/FRT80B | 60 | N=3 | | |
| Figure 3A: p{hs:FLP}/+; *RpS17* p{arm:LacZ} FRT80B/FRT80B | 20 | peIF2a: N=9 | | |
| Figure 3B: p{hs:FLP}/ p{hs:FLP}; RpL27A^-^ p{arm:LacZ} FRT40/FRT40 | 20 | peIF2a: N=5 | | |
| Figure 3C, D: p{hs:FLP}/+; *RpS17* , act>CD2>Gal4 , UAS-GFP /UAS- RNAi^Xrp1^ | 10 | C. peIF2a: N=10 (for both lines)  D. OPP: N= 16 (for both lines) | | |
| Figure 3E,F: p{hs:FLP}/+; UAS-*PPP1R15*/+ ; *RpS17* , act>CD2>Gal4 , UAS-GFP /+ | 10 | E. peIF2a: N=4  F. OPP: N= 4 | | |
| Figure 3G: p{hs:FLP}/+; UAS- RNAi^PERK^ /+ ;act>CD2>Gal4 , UAS-GFP /+ | 15 | peIF2a: N=5 | | |
| Figure 3H, I: p{hs:FLP}/+; UAS- RNAi^PERK^ /+ ; *RpS17* , act>CD2>Gal4 , UAS-GFP /+ | 15 | H. peIF2a: N= 8  I. OPP: N= 4 | | |
| Figure 3J: p{hs:FLP}/+; UAS- RNAi^Gcn2^/+ ; *RpS17* , act>CD2>Gal4 , UAS-GFP /+ | 15 | peIF2a: N= 3 | | |
| Figure 3 - Figure supplement 1A: p{hs:FLP}/ p{hs:FLP}; FRT82 *RpS3* p{arm:LacZ} /FRT82B | 20 | peIF2a: N= 9 | | |
| Figure 3 - Figure supplement 1B: p{hs:FLP}/ p{hs:FLP}; FRT42 *RpS18* p{Ubi:GFP}/FRT42 | 20 | peIF2a: N= 6 | | |
| Figure 3 - Figure supplement 1C: p{hs:FLP}/ p{hs:FLP}; FRT82 *RpS3* p{arm:LacZ} /FRT82B Xrp1^M2-73^ | 20 | peIF2a: N= 6 | | |
| Figure 3 - Figure supplement 1D: p{hs:FLP}/ p{hs:FLP}; *RpS17* FRT80B/p{arm:LacZ} FRT80B | 20 | peIF2a: N=4 | | |
| Figure 3 - Figure supplement 1E: p{hs:FLP}/ p{hs:FLP}; *RpS17* FRT80B/p{arm:LacZ} FRT80B *Xrp1^M2^*^-73^ | 20 | peIF2a: N= 3 | | |
| Figure 3 - Figure supplement 1F,G: p{hs:FLP}/+; *RpS17* , act>CD2>Gal4 , UAS-GFP /UAS- RNAi*^Irbp18^* | 10 | F. peIF2a: N=7  G. OPP: N=5 | | |
| Figure 3 - Figure supplement 1H: en-GAL4, UAS-GFP /UAS-PPP1R15; FRT82 *RpS3*/+ | No hs | peIF2a: N=2 | | |
| Figure 3 - Figure supplement 1I: *RpS18*^-^ ,en-GAL4, UAS-GFP /UAS-PPP1R15 | No hs | peIF2a: N=3 | | |
| Figure 3 - Figure supplement 1J: en-GAL4, UAS-GFP / UAS- RNAi^PERK^; FRT82 *RpS3*/+ | No hs | peIF2a: N=3 | | |
| Figure 3 - Figure supplement 1K: *RpS18*^-^ ,en-GAL4, UAS-GFP /UAS- RNAi^PERK^ | No hs | peIF2a: N=1 | | |
| Figure 3 - Figure supplement 1L: p{hs:FLP}/+; UAS- RNAi^PERK^ /*RpS18*^-^ ; act>CD2>Gal4, UAS-His-RFP/+ | 10 | OPP. N=2 | | |
| Figure 3 - Figure supplement 1M, N: p{hs:FLP}/ p{hs:FLP}; FRT80B/p{arm:LacZ} FRT80B | 30 | M. peIF2a: N=4  N. OPP: N=7 | | |
| Figure 4A: p{hs:FLP}/ p{hs:FLP}; FRT42 *RpS18* p{Ubi:GFP}/FRT42 | 20 | p62: N=3 | | |
| Figure 4B: p{hs:FLP}/ p{hs:FLP}; FRT82 *RpS3* p{arm:LacZ} /FRT82B | 20 | p62: N=7 | | |
| Figure 4C: p{hs:FLP}/ p{hs:FLP}; RpL27A^-^ p{arm:LacZ} FRT40/FRT40 | 20 | p62: N=4 | | |
| Figure 4D: p{hs:FLP}/+; UAS- RNAi^Xrp1^ / GstD-lacZ, *RpS18*^-^ ; act>CD2>Gal4, UAS-GFP /+ | 15 | p62: N=7 | | |
| Figure 4E: p{hs:FLP}/ p{hs:FLP}; FRT82 *RpS3* p{arm:LacZ} /FRT82B Xrp1^M2-73^ | 20 | p62: N=5 | | |
| Figure 4F: p{hs:FLP}/+; UAS- RNAi^PERK^ / GstD-lacZ, *RpS18*^-^ ; act>CD2>Gal4, UAS-GFP /+ | 15 | p62: N=8 | | |
| Figure 4G, H: ***wt genotype***: w ^1-18^ /+; FRT82B/+, |  | N=3 | | |
| Figure 4G, H: ***RpS17/+ genotype***: w 1-18 /y w p{hs:FLP}; *RpS17* p{ubi:GFP} FRT80B/+ | No hs | N=3 | | |
| Figure 4G, H: ***RpS3/+ genotype***: w 1-18 /y w p{hs:FLP}; FRT82 *RpS3* p{arm:LacZ/+ | No hs | N=3 | | |
| Figure 4G, H: ***RpS3/+, Xrp1[M2-73]/+ genotype***: w 1-18 /y w p{hs:FLP}; FRT82 *RpS3* p{arm:LacZ}/ FRT82B Xrp1 ^M2-73^ | No hs | N=3 | | |
| Figure 4 - Figure supplement 1A: Xbp1-EGFP/nubGal4; +/+ | No hs | N=7 (all weak) | | |
| Figure 4 - Figure supplement 1B: Xbp1-EGFP/nubGal4; *RpS17* p{arm:LacZ} FRT80B /+ | No hs | N=7 (4 weak and 3 strong) | | |
| Figure 4 - Figure supplement 1C: Xbp1-EGFP/nubGal4; FRT82 *RpS3* p{arm:LacZ} /+ | No hs | N=5 (all weak) | | |
| Figure 4 - Figure supplement 1D: p{hs:FLP}/ p{hs:FLP}; FRT82 *RpS3* p{arm:LacZ} /FRT82B | 20 | N=5 | | |
| Figure 5A: {hs:FLP}/+; act>CD2>Gal4 , UAS-GFP / UAS – RNAi^w^ | 7 | N=5 | | |
| Figure 5B: {hs:FLP}/+; act>CD2>Gal4 , UAS-GFP / UAS – RNAi^PPP1R15^ (line: BL 33011) (5A and 5B samples were processed on the same day) | 7 | N=6 | | |
| Figure 5C: {hs:FLP}/+; UAS – RNAi^PPP1R15^ /TRE-dsRED ; act>CD2>Gal4 , UAS-GFP /+ (line: v107545) (processed in parallel with 5J) | 10-15 | p-eIF2a: N=10 (v107545)  peIF2a: N=12 (33011) | | |
| Figure 5D: {hs:FLP}/+; act>CD2>Gal4 , UAS-GFP / UAS – RNAi^PPP1R15^ (line: BL 33011) | 25±5 | OPP. N=10 | | |
| Figure 5E: {hs:FLP}/+; UAS – RNAi^PPP1R15^ /+ ; act>CD2>Gal4 , UAS-GFP /+ (line: v107545) | 25±5 | Dcp1. N=12 (v107545)  Dcp1: N=13 (33011) | | |
| Figure 5F: nubGal4, UAS-RFP/+; Xrp1-HA/UAS-RNAi ^w^ | No hs | N=6 | | |
| Figure 5G: nubGal4, UAS-RFP/ UAS – RNAi^PPP1R15^; Xrp1-HA/+ (line: v107545) | No hs | Xrp1-HA: N=6 (line v107545) | | |
| Figure 5H, J, K: {hs:FLP}/+; UAS – RNAi^PPP1R15^ / UAS-RNAi^Xrp1^; act>CD2>Gal4 , UAS-GFP /+ (line RNAi^PPP1R15^: v107545 and line RNAi^Xrp1^: v107860) (5H processed in parallel with 5I and 5J processed in parallel with 5I. Also, 5K processed in parallel with Figure 5 in Supplemental 1B) | 10-15 | H.Dcp1: N=8 (completely rescued)+1 (partially rescued)  J. peIF2a:3 (partially rescued)  K. OPP:4 | | |
| Figure 5I : {hs:FLP}/+; UAS – RNAi^PPP1R15^ /TRE-dsRED; act>CD2>Gal4 , UAS-GFP /+ (line RNAi^PPP1R15^: v107545) | 10-15 | I. Dcp1: N=12 (v107545) | | |
| Figure 5 - Figure supplement 1A: {hs:FLP}/+; act>CD2>Gal4 , UAS-GFP / UAS – RNAi^PPP1R15^ (line: BL 33011) | 25±5 | p-eIF2a: N=10 (v107545)  peIF2a: N=12 (33011) | | |
| Figure 5 - Figure supplement 1B: {hs:FLP}/+; UAS – RNAi^PPP1R15^ /+ ; act>CD2>Gal4 , UAS-GFP /+ (line: v107545) (processed in parallel with Figure 5K) | 10-15 | OPP. N=10 | | |
| Figure 5 - Figure supplement 1C: {hs:FLP}/+; UAS – RNAi^PPP1R15^ /+ ; act>CD2>Gal4 , UAS-GFP /+ (line: v107545) (basal side of the same disc in Figure 5E) | 25±5 | Dcp1. N=12 (v107545)  Dcp1: N=13 (33011) | | |
| Figure 5 - Figure supplement 1D: nubGal4, UAS-RFP/ UAS – RNAi^PPP1R15^; Xrp1-HA/+ (line: Bl 33011) | No hs | Xrp1-HA: N=6 (line 33011) | | |
| Figure 5 - Figure supplement 1E: {hs:FLP}/+; UAS – RNAi^PPP1R15^ / UAS-RNAi^Xrp1^; act>CD2>Gal4 , UAS-GFP /+  (line RNAi^PPP1R15^: v107545 and line RNAi^Xrp1^: v107860) (basal side of the same disc in Figure 5H) | 10-15 | Dcp1: N=8 (completely rescued)+1 (partially rescued) | | |
| Figure 5 - Figure supplement 1F: {hs:FLP}/+; UAS – RNAi^PPP1R15^ /+ ; act>CD2>Gal4 , UAS-GFP /+ (line RNAi^PPP1R15^: v107545)  (basal side of the same disc in Figure 5I) | 10-15 | Dcp1: N=12 (v107545) | | |
| Figure 5 - Figure supplement 2: p{hs:FLP}/+; UAS- RNAi ^PPP1R15^/+ ;act>CD2>Gal4 , UAS- GFP /+ (line: Bl 33011) | 30 | N=2 | | |
| Figure 5 - Figure supplement 3A, A’: y w, hs-FLP; Act5C > GAL4-w, UAS-mCherry-CAAX, Df(2R)M60E; FRT82B, ubi-GFP-nls, tubP-GAL80, M{RpL19 genomic}ZH-86Fb/FRT82B | 20 | N=4 | | |
| Figure 5 - Figure supplement 3B, B’: y w, hs-FLP; Act5C > GAL4-w, UAS-mCherry-CAAX, Df(2R)M60E; FRT82B, ubi-GFP-nls, tubP-GAL80, M{RpL19 genomic}ZH-86Fb/FRT82B Perk^null^ | 20 | N=4 | | |
| Figure 5 - Figure supplement 3C: FM7/hs-FLP; FRT82B p{RpL36^+^} p{arm:LacZ}/FRT82B | 20 | N=1 | | |
| Figure 5 - Figure supplement 3D: Df(1)R194/hs-FLP; FRT82B p{RpL36^+^} p{arm:LacZ}/FRT82B | 20 | N=3 | | |
| Figure 5 - Figure supplement 3E: Df(1)R194/hs-FLP; FRT82B p{RpL36^+^} p{arm:LacZ}/FRT82B PERK^null^ | 20 | N=3 | | |
| Figure 5 - Figure supplement 3F: FM7/hs-FLP; FRT82B p{RpL36^+^} p{arm:LacZ}/FRT82B PERK^null^ | 20 | N=1 | | |
| Figure 6A: *RpS18*^-^ ,en-GAL4, UAS-GFP / UAS- RNAi^PERK^; Xrp1-HA /+ | No hs | HA. N=7 | | |
| Figure 6B: *RpS18*^-^ ,en-GAL4, UAS-GFP / UAS-PPP1R15; Xrp1-HA /+ | No hs | HA. N=4 | | |
| Figure 6C: *RpS18*^-^ ,en-GAL4, UAS-GFP /+; Xrp1-HA /UAS- RNAi*^Irbp18^* | No hs | HA. N=4 | | |
| Figure 6D: *RpS18*^-^ ,en-GAL4, UAS-GFP /+; Xrp1-HA /UAS- RNAi*^w^* | No hs | HA. N=6 | | |
| Figure 6 Supplement 1A: p{hs:FLP}/+; TRE-dsRED/+; *RpS17* , act>CD2>Gal4 , UAS-GFP /UAS- RNAi*^w^* | 15 | TRE: N= 6 | | |
| Figure 6 Supplement 1B: p{hs:FLP}/+; TRE-dsRED/+; *RpS17* , act>CD2>Gal4 , UAS-GFP /UAS- Bsk^DN^ | 15 | TRE: N= 4 | | |
| Figure 6 Supplement 1C: p{hs:FLP}/+; TRE-dsRED/+; *RpS17* , act>CD2>Gal4 , UAS-GFP /UAS- RNAi*^Irbp18^* | 15 | TRE: N= 5 | | |
| Figure 6 Supplement 1D: p{hs:FLP}/+; TRE-dsRED/ UAS- RNAi*^PERK^*; *RpS17* , act>CD2>Gal4 , UAS-GFP /+ | 15 | TRE: N= 7 | | |
| Figure 6 Supplement 2A: p{hs:FLP}/+; GstD1-lacZ, *RpS18*^-^ /+ ; act>CD2>Gal4, UAS-GFP /UAS-RNAi^w^ | 15 | GstD: N=4 | | |
| Figure 6 Supplement 2B: p{hs:FLP}/+; UAS- RNAi^Xrp1^ / GstD1-lacZ, *RpS18*^-^ ; act>CD2>Gal4, UAS-GFP /+ | 15 | GstD: N=3 | | |
| Figure 6 Supplement 2C: p{hs:FLP}/+; UAS- RNAi^PERK^ / GstD1-lacZ, *RpS18*^-^ ; act>CD2>Gal4, UAS-GFP /+ | 15 | GstD: N=6 | | |
| Figure 7A, B, D: {hs:FLP}/+; UAS – RNAi^eIF4G^ /+ ; act>CD2>Gal4 , UAS-GFP /+ (line: v17002) | 25±5 | A. OPP: N=9  B. Dcp1:N=17  D. peIF2a:N=13 | | |
| Figure 7C: {hs:FLP}/+; UAS – RNAi^eIF4G^ /+ ; act>CD2>Gal4 , UAS-GFP /Xrp1-HA (line: v17002) | 25±5 | C. HA: N=6 | | |
| Figure 7E, F, H: {hs:FLP}/+; UAS – RNAi^eEF2^ /+ ; act>CD2>Gal4 , UAS-GFP /+ (line: v107268) | 25±5 | E. OPP: N=11  F. Dcp1:N=10  H. peIF2a:N=9 | | |
| Figure 7G: {hs:FLP}/+; UAS – RNAi^eEF2^ /+ ; act>CD2>Gal4 , UAS-GFP / Xrp1-HA (line: v107268) | 25±5 | G. HA: N=7 | | |
| Figure 7I, J, L: {hs:FLP}/+; UAS – RNAi^eIF6^ /+ ; act>CD2>Gal4 , UAS-GFP /+ (line: v108094) | 25±5 | I. OPP: N=11  J. Dcp1:N=12  L. peIF2a:N= 8 | | |
| Figure 7K: {hs:FLP}/+; UAS – RNAi^eIF6^ /+ ; act>CD2>Gal4 , UAS-GFP / Xrp1-HA (line: v108094) | 25±5 | K. HA: N=10 | | |
| Figure 7M, N: p{hs:FLP}/+; UAS-RNAi^TAF1B^/+ ;act>CD2>Gal4 , UAS- GFP /+ (line: Bl 61957) | 25±5 | M. OPP: N=17  N. pRpS6: N=3 | | |
| Figure 7 - Figure supplement 1A, B, D: {hs:FLP}/+; UAS – RNAi^eIF5A^ /+ ; act>CD2>Gal4 , UAS-GFP / + (line: v101513) | 25±5 | A. OPP: N=17  B. Dcp1:N=8  D. peIF2a:N=11 | | |
| Figure 7 - Figure supplement 1C: {hs:FLP}/+; UAS – RNAi ^eIF5A^ /+ ; act>CD2>Gal4 , UAS-GFP / Xrp1-HA (line: v101513) | 25±5 | C. HA: N=8 | | |
| Figure 7 - Figure supplement 1E, F, H: {hs:FLP}/+; UAS – RNAi ^eEF1α^ /TRE-dsRED; act>CD2>Gal4 , UAS-GFP / + (line: v104502) | 25±5 | E. OPP: N=5 (increased OPP), N=8 (decreased OPP)  F. Dcp1:N=10  H. peIF2a:N=12 | | |
| Figure 7 - Figure supplement 1G: {hs:FLP}/+; UAS – RNAi ^eEF1α^ /+ ; act>CD2>Gal4 , UAS-GFP / Xrp1-HA (line: v104502) | 25±5 | G. HA: N=7 | | |
| Figure 7 - Figure supplement 1I: p{hs:FLP}/+; UAS- RNAi^TAF1B^ /+ ;act>CD2>Gal4 , UAS- GFP / Xrp1-HA (line: v105873) | 25±5 | HA: N=13 | | |
| Figure 7 - Figure supplement 1J: p{hs:FLP}/+; UAS- RNAi^TAF1B^ /+ ;act>CD2>Gal4 , UAS- GFP /+ (line: Bl 61957) | 25±5 | peIF2a: N=8 (for both lines) | | |
| Figure 7 - Figure supplement 3A: nubGal4, UAS-RFP/+ ; Xrp1-HA/RNAi^w^ | No hs | N=9 | | |
| Figure 7 - Figure supplement 3B: nubGal4, UAS-RFP/ UAS – RNAi^eIF4G^ ; Xrp1-HA/+ | No hs | N=6 | | |
| Figure 7 - Figure supplement 3C: nubGal4, UAS-RFP/ UAS –RNAi^eEF2^ ; Xrp1-HA/+ | No hs | N=6 | | |
| Figure 7 - Figure supplement 3D: nubGal4, UAS-RFP/ UAS – RNAi^eEF1α1^ ; Xrp1-HA/+ | No hs | N=6 | | |
| Figure 7 - Figure supplement 3E: nubGal4, UAS-RFP/ UAS – RNAi ^eIF5A^ ; Xrp1-HA/+ | No hs | N=6 | | |
| Figure 7 - Figure supplement 3F: nubGal4, UAS-RFP/ UAS – RNAi ^eIF6^ ; Xrp1-HA/+ | No hs | N=10 | | |
| Figure 7 - Figure supplement 3G: nubGal4, UAS-RFP/ RNAi^TAF1B^ ; Xrp1-HA/+ (v105873) | No hs | N=6 | | |
| Figure 7 - Figure supplement 3H: nubGal4, UAS-RFP/ RNAi^TAF1B^ ; Xrp1-HA/+ (line: Bl 61957) | No hs | N=8 | | |
| Figure 8A-C: {hs:FLP}/+; UAS – RNAi^eEF2^ /UAS-PPP1R15 ; act>CD2>Gal4 , UAS-GFP / + | 25±5 | A.peIF2a: N=3  B.OPP: N=3  C.Dcp1: N=7 | | |
| Figure 8D-F: {hs:FLP}/+; UAS – RNAi^eIF4G^ /UAS-PPP1R15 ; act>CD2>Gal4 , UAS-GFP / + | 25±5 | D.peIF2a: N=11  E.OPP: N=4  F.Dcp1: N=8 | | |
| Figure 8G-I: {hs:FLP}/+; UAS – RNAi^eIF6^ /UAS-PPP1R15 ; act>CD2>Gal4 , UAS-GFP / + | 25±5 | G.peIF2a: N=3  H.OPP: N=9  I.Dcp1: N=6 | | |
| Figure 8J: {hs:FLP}/+; UAS – RNAi^eEF2^ /UAS-PPP1R15 ; act>CD2>Gal4 , UAS-GFP / Xrp1-HA | 25±5 | J. HA: N=7 | | |
| Figure 8K: {hs:FLP}/+; UAS – RNAi^eIF4G^ /UAS-PPP1R15 ; act>CD2>Gal4 , UAS-GFP / Xrp1-HA | 25±5 | K. HA: N=4 | | |
| Figure 8L: {hs:FLP}/+; UAS – RNAi^eIF6^ /UAS-PPP1R15 ; act>CD2>Gal4 , UAS-GFP / Xrp1-HA | 25±5 | L. HA: N=5 | | |
| Figure 8M-O: {hs:FLP}/+; UAS – RNAi^eEF2^ /UAS- RNAi^Xrp1^ ; act>CD2>Gal4 , UAS-GFP /+ | 25±5 | M.peIF2a: N=6  N.OPP: N=6  O.Dcp1: N=10 | | |
| Figure 8P-R: {hs:FLP}/+; UAS – RNAi^eIF4G^ /UAS- RNAi^Xrp1^ ; act>CD2>Gal4 , UAS-GFP / + | 25±5 | P.peIF2a: N=6  Q.OPP: N=7  R.Dcp1: N=10 | | |
| Figure 8S-U: {hs:FLP}/+; UAS – RNAi^eIF6^ /UAS- RNAi^Xrp1^ ; act>CD2>Gal4 , UAS-GFP / + | 25±5 | S.peIF2a: N=8  T.OPP: N= 6 (same as WT)+ 3 (higher than WT)  U.Dcp1: N=11 | | |
| Figure 8 - Figure supplement 1A-C: {hs:FLP}/+; act>CD2>Gal4 , UAS-GFP / UAS – RNAi^w^, | 25±5 | A.peIF2a: N=3  B.OPP: N=14  C.Dcp1: N=15 | | |
| Figure 8 - Figure supplement 1D-F: {hs:FLP}/+; UAS-PPP1R15/+; act>CD2>Gal4 , UAS-GFP / UAS – RNAi^w^, | 25±5 | D.peIF2a: N=3  E.OPP: N=5  F.Dcp1: N=11 | | |
| Figure 8 - Figure supplement 1G-I: {hs:FLP}/+; UAS – RNAi^eEF1α1^ / UAS-PPP1R15 ; act>CD2>Gal4 , UAS-GFP /+ | 25±5 | G.peIF2a: N=4  H.OPP: N=2 (still decreased OPP) + 3 (increased OPP, clones are tiny)  I.Dcp1: N=3 | | |
| Figure 8 - Figure supplement 1J-L: {hs:FLP}/+; UAS – RNAi ^eIF5A^ / UAS-PPP1R15 ; act>CD2>Gal4 , UAS-GFP /+ | 25±5 | J.peIF2a: N=6  K.OPP: N=9  L.Dcp1: N=4 | | |
| Figure 8 - Figure supplement 1M-O: {hs:FLP}/+; UAS – RNAi ^TAF1B^ / UAS-PPP1R15 ; act>CD2>Gal4 , UAS-GFP /+ (v105873) | 25±5 | M.peIF2a: N=18  N.OPP: N=9  O.Dcp1: N=10 | | |
| Figure 8 - Figure supplement 1P: {hs:FLP}/+; UAS – RNAi^eEF1α1^ / UAS-PPP1R15 ; act>CD2>Gal4 , UAS-GFP /Xrp1-HA | 25±5 | HA: N=3 | | |
| Figure 8 - Figure supplement 1Q: {hs:FLP}/+; UAS – RNAi^eIF5A^ / UAS-PPP1R15 ; act>CD2>Gal4 , UAS-GFP /Xrp1-HA | 25±5 | HA: N=4 | | |
| Figure 8 - Figure supplement 1R: {hs:FLP}/+; UAS – RNAi^TAF1B^ / UAS-PPP1R15 ; act>CD2>Gal4 , UAS-GFP /Xrp1-HA (v105873) | 25±5 | HA: N=6 | | |
| Figure 8 - Figure supplement 2A: {hs:FLP}/+; UAS – RNAi^eEF2^ /UAS-RNAi^PERK^ ; act>CD2>Gal4 , UAS-GFP / Xrp1-HA | 25±5 | HA: N=4 | | |
| Figure 8 - Figure supplement 2B: {hs:FLP}/+; UAS – RNAi^eEF2^ /UAS-RNAi^PERK^ ; act>CD2>Gal4 , UAS-GFP /+ | 25±5 | Dcp1: N=4 | | |
| Figure 8 - Figure supplement 2C: {hs:FLP}/+; UAS – RNAi^eIF4G^ /UAS-RNAi^PERK^ ; act>CD2>Gal4 , UAS-GFP / Xrp1-HA | 25±5 | HA: N=4 | | |
| Figure 8 - Figure supplement 2D: {hs:FLP}/+; UAS – RNAi^eIF4G^ /UAS-RNAi^PERK^ ; act>CD2>Gal4 , UAS-GFP /+ | 25±5 | Dcp1: N=4 | | |
| Figure 8 - Figure supplement 2E: {hs:FLP}/+; UAS – RNAi^eIF6^/UAS-RNAi^PERK^ ; act>CD2>Gal4 , UAS-GFP / Xrp1-HA | 25±5 | HA: N=6 | | |
| Figure 8 - Figure supplement 2F: {hs:FLP}/+; UAS – RNAi^eIF6^ /UAS-RNAi^PERK^ ; act>CD2>Gal4 , UAS-GFP /+ | 25±5 | Dcp1: N=6 | | |
| Figure 8 - Figure supplement 2G: {hs:FLP}/+; UAS – RNAi ^eEF1α1^/UAS-RNAi^PERK^ ; act>CD2>Gal4 , UAS-GFP / Xrp1-HA | 25±5 | HA: N=5 | | |
| Figure 8 - Figure supplement 2H: {hs:FLP}/+; UAS – RNAi^eEF1α1^ /UAS-RNAi^PERK^ ; act>CD2>Gal4 , UAS-GFP /+ | 25±5 | Dcp1: N=5 | | |
| Figure 8 - Figure supplement 2I: {hs:FLP}/+; UAS – RNAi^eIF5A^/UAS-RNAi^PERK^ ; act>CD2>Gal4 , UAS-GFP / Xrp1-HA | 25±5 | HA: N=6 | | |
| Figure 8 - Figure supplement 2J: {hs:FLP}/+; UAS – RNAi^eIF5A^ /UAS-RNAi^PERK^ ; act>CD2>Gal4 , UAS-GFP /+ | 25±5 | Dcp1: N=6 | | |
| Figure 8 - Figure supplement 2K: {hs:FLP}/+; UAS – RNAi^TAF1B^/UAS-RNAi^PERK^ ; act>CD2>Gal4 , UAS-GFP / Xrp1-HA (v105873) | 25±5 | Xrp1-HA=7 | | |
| Figure 8 - Figure supplement 2L: {hs:FLP}/+; UAS – RNAi^TAF1B^ /UAS-RNAi^PERK^ ; act>CD2>Gal4 , UAS-GFP /+ (v105873) | 25±5 | Dcp1=7 | | |
| Figure 8 - Figure supplement 3A-C: {hs:FLP}/+; UAS-RNAi^Xrp1^/+ ; act>CD2>Gal4 , UAS-GFP /UAS-RNAi^w^ | 25±5 | A.peIF2a: N=4  B.OPP: N=7  C.Dcp1: N=9 | | |
| Figure 8 - Figure supplement 3D-F: {hs:FLP}/+; UAS – RNAi^eEF1α^ / UAS-RNAi^Xrp1^ ; act>CD2>Gal4 , UAS-GFP /+ | 25±5 | D.peIF2a: N=7 (partly rescued)  E.OPP: N=4 (all increase)  F.Dcp1: N=7 | | |
| Figure 8 - Figure supplement 3G-I: {hs:FLP}/+; UAS – RNAi^eIF5A^ / UAS-RNAi^Xrp1^ ; act>CD2>Gal4 , UAS-GFP /+ | 25±5 | E.peIF2a: N=5 (little bit reduced, not completely rescued by Xrp1-RNAi)  F.OPP: N=1  G.Dcp1: N=9 (boundary cell is reduced significantly but cell death was seen inside the clones) | | |
| Figure 8 - Figure supplement 3J, K: {hs:FLP}/+; UAS – RNAi^TAF1B^ / UAS-RNAi^Xrp1^ ; act>CD2>Gal4 , UAS-GFP /+ (v105873) | 25±5 | J. peIF2a=1  K.OPP=2 | | |
| Figure 8 - Figure supplement 3L: {hs:FLP}/+; UAS – RNAi^TAF1B^ / + ; act>CD2>Gal4 , UAS-GFP /+ (v105873) | 25±5 | OPP=17 | | |
| Figure 9A: nubGal4, UAS-RFP/+; Xrp1-HA/Xrp1-HA | No hs | N=6 | | |
| Figure 9B: nubGal4, UAS-RFP/ UAS – RNAi^TAF1B^;Xrp1-HA/ Xrp1-HA (line: v105873) | No hs | N=6 | | |
| Figure 9C: nubGal4, UAS-RFP/ UAS –RNAi^TAF1B^; *Rps12^G97D^*, Xrp1-HA/ *Rps12^G97D^*, Xrp1-HA (line: v105873) | No hs | N=6 | | |
| Figure 9D: nubGal4, UAS-RFP/ UAS – RNAi^eEF2^;Xrp1-HA/ Xrp1-HA | No hs | N=6 | | |
| Figure 9E: nubGal4, UAS-RFP/ UAS –RNAi^eEF2^; *Rps12^G97D^*, Xrp1-HA/ *Rps12^G97D^,* Xrp1-HA | No hs | N=9 | | |
| Figure 10F: GstD1-GFP; | No hs | N=5 | | |
| Figure 10G: GstD1-GFP/+; FRT82 *RpS3* p{arm:LacZ}/+ | No hs | N=5 | | |
| Figure 10H: GstD1ΔARE-GFP/+; +/+ | No hs | N=5 | | |
| Figure 10I: GstD1ΔARE-GFP/+; FRT82 *RpS3* p{arm:LacZ}/+ | No hs | N=5 | | |
| Figure 10K: GstD1-GFP; | No hs | N=5 | | |
| Figure 10L: GstD1-GFP; *RpS17* p{arm:LacZ} FRT80B/+ | No hs | N=6 | | |
| Figure 10M: GstD1 Xrp1m -GFP | No hs | N=5 | | |
| Figure 10N: GstD1Xrp1m-GFP; *RpS17* p{arm:LacZ} FRT80B/+ | No hs | N=6 | | |
| Figure 10P and Figure 10 Supplement 3 genotypes per column:  1^st^ column: w^11-18^ ; FRT82B/+  2^nd^ column: w^11-18^ ; w p{hs:FLP}; *RpS17* p{ubi:GFP} FRT80B/+  3^rd^ column: w^11-18^ ;w p{hs:FLP}; FRT82 *RpS3* p{arm:LacZ/+  4^th^ column: w^11-18^ ;w p{hs:FLP}; FRT82 *RpS3* p{arm:LacZ/FRT82B FRT82B *Xrp1^M2^*^-73^  5^th^ column: w^11-18^; FRT82B *Xrp1^M2^*^-73^ / +  6^th^ column: w^11-18^; *rpS12^D97^* FRT80B / *rpS12^D97^* FRT80B  7^th^ column: w^11-18^; *rpS12^D97^* FRT80B / *rpS12^D97^* FRT80B *RpS3* | No hs | 3 biological replicates for each genotype | | |
